# Rhodium-catalyzed C–H functionalization of heteroarenes using indoleBX hypervalent iodine reagents

**DOI:** 10.3762/bjoc.14.102

**Published:** 2018-05-25

**Authors:** Erwann Grenet, Ashis Das, Paola Caramenti, Jérôme Waser

**Affiliations:** 1 Laboratory of Catalysis and Organic Synthesis, Institute of Chemical Sciences and Engineering, Ecole Polytechnique Fedérale de Lausanne, EPFL SB ISIC LCSO, BCH 4306, 1015 Lausanne, Switzerland

**Keywords:** C–H activation, hypervalent iodine, indoleBX, indoles, pyridinones, rhodium catalysis

## Abstract

The C–H indolation of heteroarenes was realized using the benziodoxolone hypervalent iodine reagents indoleBXs. Functionalization of the C–H bond in bipyridinones and quinoline *N*-oxides catalyzed by a rhodium complex allowed to incorporate indole rings into aza-heteroaromatic compounds. These new transformations displayed complete regioselectivity for the C-6 position of bipyridinones and the C-8 position of quinoline *N*-oxides and tolerated a broad range of functionalities, such as halogens, ethers, or trifluoromethyl groups.

## Introduction

**Figure 1:**
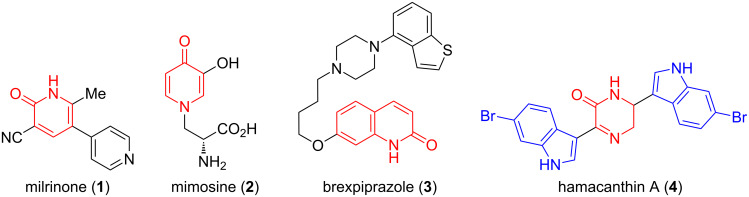
Bioactive compounds with pyridinone, quinolone and indole cores.

Nitrogen-containing heteroaromatic compounds have valuable properties in medicinal chemistry, pharmacology and functional materials. Among those, pyridinone, sometimes called pyridone, is a key structural motif of well-known active compounds and natural products (Figure 1) [1]. For example, the 2-pyridinone ring is present in milrinone (**1**), used to treat heart failure, while a 4-pyridinone is part of mimosine (**2**), an alkaloid isolated from *Mimosa pudica*. A benzene-fused pyridinone – a quinolone – can be found in brexpiprazole (**3**), a drug used against schizophrenia. In addition, the indole core is also omnipresent in bioactive compounds [2]. It can be directly bound to other heterocycles, such as a dihydropyrazidinone in hamacanthine A (**4**) (Figure 1) [3]. Due to their occurrence in biologically active compounds, it is therefore attractive to develop new methods to functionalize pyridinones. The introduction of further heterocyclic rings, such as indoles, is particularly attractive.

Most of the methods for indolylpyridinone synthesis involve a condensation cascade process to generate the pyridinone ring [4–6]. These methods usually require an electron-withdrawing group (nitrile, nitro, carbonyl), which ends up on the pyridinone ring. As alternative, a Suzuki–Miyaura coupling between 3-halogenoindoles and (2-methoxypyridyl)boronic acids followed by a deprotection of the methoxy group [7–8] or transition-metal-catalyzed annulation methods [9] have also been reported.

**Scheme 1:**
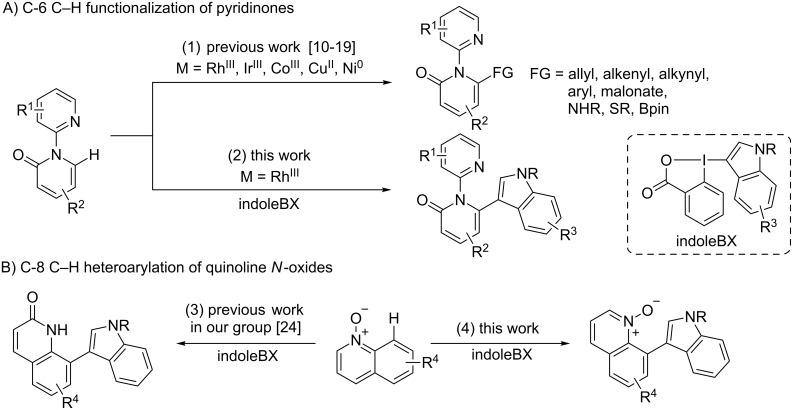
C–H functionalization of pyridinones and quinoline *N*-oxides.

In contrast, several procedures have been described for the modification of pyridinones to introduce other substituents, especially based on highly efficient C–H functionalization methods [10]. Very recently, several research groups have selectively functionalized the C-6 C–H bond by using a 2-pyridyl directing group on the nitrogen and a transition metal catalyst (reaction 1, Scheme 1A) [11–19]. In particular, Li and co-workers have used ethynylbenziodoxolone (EBX) hypervalent iodine reagents to achieve a regiodivergent alkynylation of the pyridinone core employing either a gold(I) or a rhodium(III) catalyst for C-5 and C-6 functionalization, respectively [13]. Hypervalent iodine reagents in general [20], and benziodoxole derivatives in particular [21], have found broad application in synthetic chemistry. Aryl iodonium salts have been used successfully in transition-metal-catalyzed transformations [22], but only one application of indole iodonium salts in copper catalysis by You and co-workers had been reported until 2017 [23]. In this context, indole-based benziodoxole hypervalent iodine reagents, recently introduced by Yoshikai's and our group [24–27], appeared ideal partners to develop a new C–H heteroarylation of pyridinones.

Herein, we report the selective C–H heteroarylation of the C-6 position of bipyridinones by a rhodium-catalyzed reaction with indoleBX (reaction 2, Scheme 1A). In addition, we demonstrate that the mild conditions developed allow the heteroarylation of the C-8 position of quinoline *N*-oxides, whereas formation of the quinolinone had been observed in our previous work (Scheme 1B). The obtained products combine up to three classes of privileged heterocycles in medicinal chemistry in a single compound, and are therefore expected to be highly useful building blocks in the search for new bioactive compounds.

## Results and Discussion

We started the studies on C–H indolation with the optimization of the reactions conditions (Table 1) for the coupling of [1,2'-bipyridin]-2-one (**5a**) with Me-indoleBX **6a**, easily obtained from commercially available 1-methylindole and 2-iodobenzoic acid [24]. While the reaction conditions previously developed in our group for the C–H functionalization of 2-phenylpyridine [24] failed for the coupling of **5a** with **6a** (Table 1, entry 1), we were pleased to see that addition of 0.15 equiv Zn(OTf)_2_ allowed a full conversion to the desired product **7a** in 86% yield (Table 1, entry 2). The Lewis acid is supposed to weaken the O–I bond by coordination of the carboxy group in **6a**. No base was required in this case. The reaction was completely selective for the C-6 position of the pyridinone ring. Control experiments pointed out that both Lewis acid (Table 1, entry 3) and AgSbF_6_ as additive (Table 1, entry 4) were necessary for an efficient reaction. The transformation was tolerant to air (Table 1, entry 5). However, more byproducts were observed. Decreasing the temperature (Table 1, entry 6) or the catalyst loading (Table 1, entry 7) resulted in lower yields. Finally, three control experiments with 1-methylindole (**8**, Table 1, entry 8), 3-iodo-1-methylindole (**9**, Table 1, entry 9) and the poorly stable (1*H*-indol-3-yl)(phenyl)iodonium tetrafluoroborate [23] (**10**, Table 1, entry 10) did not lead to the formation of **7a**, highlighting the unique reactivity of the benziodoxolone hypervalent iodine reagent.

**Table 1:** Optimization of the C–H heteroarylation^a,b^.

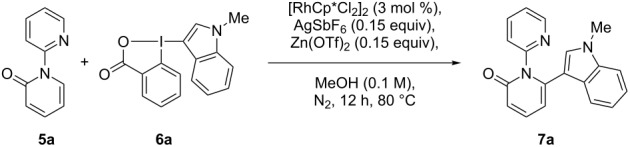		

Entry	Changes from conditions	Yield (%)

1	[RhCp*Cl_2_]_2_ (2.5 mol %), AgSbF_6_ (0.10 equiv), NaOPiv (0.10 equiv), DCE, 12 h, 50 °C	no reaction
2	–	86
3	without Zn(OTf)_2_	no reaction
4	without AgSbF_6_	60
5	under air atmosphere	80
6	60 °C	48
7	1 mol % of [RhCp*Cl_2_]_2_	75
8	1-methylindole (**8**)	no reaction
9	3-iodo-1-methylindole (**9**)	0^c^
10	iodonium salt **10**	0^c^

^a^Reactions conditions: **5** (0.20 mmol), **6** (0.20 mmol), [RhCp*Cl_2_]_2_ (3.7 mg, 6.0 µmol, 3 mol %), AgSbF_6_ (10.3 mg, 30.0 µmol, 0.15 equiv), Zn(OTf)_2_ (10.9 mg, 30.0 µmol, 0.15 equiv), methanol (2.0 mL) at 80 °C for 12 h. ^b^Isolated yield after preparative TLC. ^c^Decomposition.

**Scheme 2:**
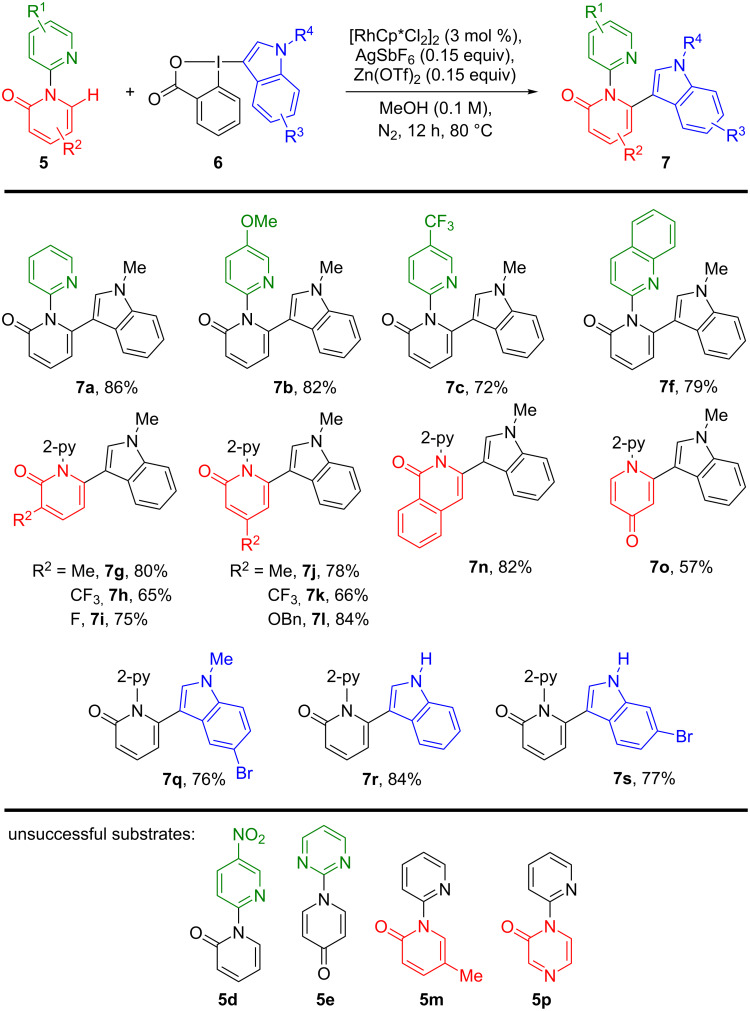
Scope and limitations of the Rh-catalyzed C–H activation of [1,2'-bipyridin]-2-one.

The scope and limitations of the reaction were then studied (Scheme 2). The diversification of the directing group was examined first. The unsubstituted pyridine group led to the formation of product **7a** in 86% yield. The electron-rich 5-methoxypyridine and the electron-poor 5-trifluoromethylpyridine directing groups gave products **7b** and **7c** in 82% and 72% yield, respectively. When a nitro group was present on the pyridine (**5d**), the product was not observed, probably due to a weaker coordination of the nitrogen on the pyridine. Pyrimidine could not be used as directing group (**5e**), confirming what has already been reported by others authors [13]. Quinoline **7f** was obtained in 79% yield. Concerning the pyridinone core, both an electron-donating methyl group and electron-withdrawing trifluoromethyl and fluoro groups (**7g–i**) were well tolerated in the C-3 position. However, the strong electron-withdrawing CF_3_ group resulted in a lower 65% yield (**7h**). This observation is also true for the C-4 position. Indeed, products **7j**–**l** were synthesized in 78% yield for a methyl, 66% yield for a trifluoromethyl and 84% yield for a benzyloxy substituent. As previously reported [13], 5-substituted pyridinone **5m** could not be functionalized. Isoquinolone **7n** was prepared in 82% yield. The methodology could also be applied to 4-pyridone in a moderate 57% yield for product **7o**. Unfortunately, pyrazin-2-one **5p** could not be functionalized. Modification of the hypervalent iodine reagent was then investigated with three selected compounds only. A bromo substituent on the benzene ring was well tolerated (**7q**). The coupling could be also performed with N–H unprotected indoleBX reagents to afford products **7r** and **7s** in 84% and 77% yield, respectively.

**Scheme 3:**
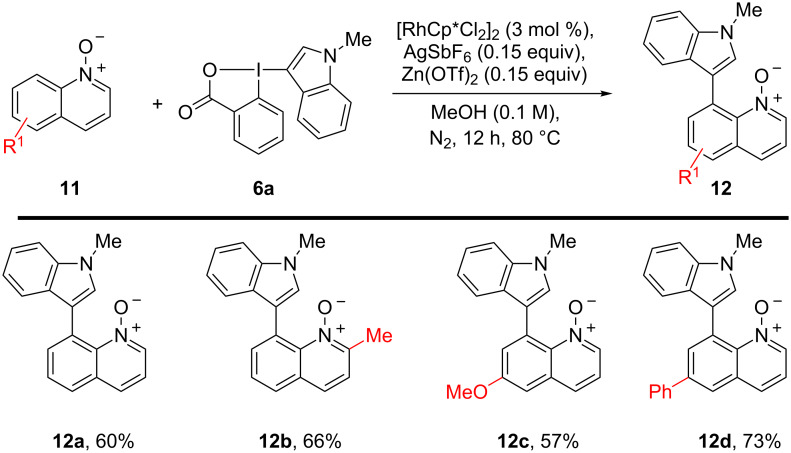
Scope of the Rh-catalyzed *peri* C–H activation of quinoline *N*-oxides.

We also applied these conditions to different quinoline *N*-oxides (Scheme 3). This class of substrates had also been used for C–H alkynylation using EBX reagents [28]. During our previous work, we had attempted the C8-heteroarylation of quinoline *N*-oxide with Me-indoleBX **6a**. However, the transformation required a temperature of 100 °C, leading to the formation of the corresponding isoquinolone in only 38% yield [24]. By employing the milder conditions developed for pyridinones, we were pleased to see that the *N*-oxide group could be preserved and product **12a** was obtained in 60% yield. A methyl substitution in C-2 position gave the product **12b** in 66% yield. In C-6 position both a methoxy and a phenyl group were well tolerated giving 57% and 73% yield of products **12c** and **12d**.

**Scheme 4:**
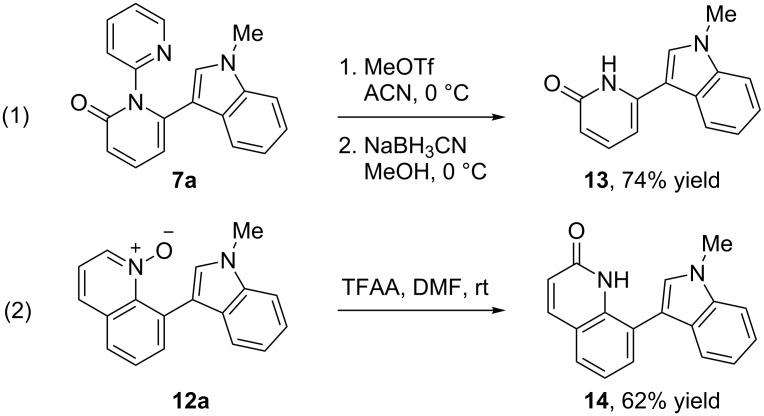
Product modifications.

The pyridine directing group could be cleaved by alkylation of the pyridine nitrogen using methyl triflate followed by reduction with sodium cyanoborohydride to deliver the N–H unprotected pyridinone **13** in 74% yield (Scheme 4) [29]. A rearrangement of the *N*-oxide furnished the corresponding isoquinolone **14** in 62% yield.

## Conclusion

In summary, we have developed the C-6 selective C–H heteroarylation of pyridin-2-ones using indoleBXs as coupling partners, [RhCp*Cl_2_]_2_ as catalyst, AgSbF_6_ as co-catalyst and Zn(OTf)_2_ as Lewis acid. The reaction could also be applied to functionalize one pyridin-4-one in C-6 position, one isoquinolinone in C-3 position and quinoline *N*-oxides in C-8 position. After cleavage of the directing group or rearrangement of the *N*-oxide function, we were able to access 6-(indol-3-yl)pyridinone and 8-(indol-3-yl)quinolone. The developed transformations give access to important heterocyclic building blocks for synthetic and medicinal chemistry and set the stages for the development of other C–H heteroarylation processes based on indoleBX reagents.

## Experimental

### General procedure for C–H heteroarylation

In a sealed tube, [RhCp*Cl_2_]_2_ (3.7 mg, 6.0 µmol, 3 mol %), AgSbF_6_ (10.3 mg, 30.0 µmol, 0.15 equiv), Zn(OTf)_2_ (10.9 mg, 30.0 µmol, 0.15 equiv), the corresponding pyridinone or quinoline *N*-oxide (0.20 mmol, 1.00 equiv) and the corresponding hypervalent iodine reagent (0.20 mmol, 1.00 equiv) were solubilized in dry MeOH (2.0 mL, 0.1 M) under N_2_ atmosphere. The mixture was stirred at 80 °C for 12 h. The mixture was then diluted with DCM (5 mL) and quenched with a saturated aqueous solution of NaHCO_3_ (5 mL). The two layers were separated and the aqueous layer was extracted twice with DCM (5 mL). The organic layers were combined, dried over magnesium sulfate dehydrate, filtered and concentrated under reduced pressure. The crude residue was purified by preparative TLC using DCM/MeOH to afford the pure desired compound.

## Supporting Information

File 1 Detailed experimental procedures, analytical data for all compounds and copies of the NMR spectra of new compounds.
