# Supplementary material for: Rhodium-catalyzed C–H functionalization of heteroarenes using indoleBX hypervalent iodine reagents
Source: Beilstein J Org Chem. 2018 May 25;14:1208–14. doi: 10.3762/bjoc.14.102 (PMC6009173; doi:10.3762/bjoc.14.102)
Supplement: File 1 — Detailed experimental procedures, analytical data for all compounds and copies of the NMR spectra of new compounds. [file Beilstein_J_Org_Chem-14-1208-s001.pdf]

Supporting Information

for

Rhodium-catalyzed C–H functionalization of heteroarenes using indoleBX hypervalent iodine reagents

Erwann Grenet, Ashis Das, Paola Caramenti and Jérôme Waser*

Address: Laboratory of Catalysis and Organic Synthesis, Institute of Chemical Sciences and Engineering, Ecole Polytechnique Fedérale de Lausanne, EPFL SB ISIC LCSO, BCH 4306, 1015 Lausanne, Switzerland

Email: Prof. Jérôme Waser - jerome.waser@epfl.ch

* Corresponding author

Detailed experimental procedures, analytical data for all compounds and copies of the NMR spectra of new compounds

Contents

1. General methods S2
2. Syntheses of starting materials S3
   2. 1. General procedure A for the synthesis of pyridinones S3
   2.2. General procedure B for the synthesis of *N*-alkylindoles S8
   2.3. General procedure C for the synthesis of indoleBX reagents S9
3. General procedure D for the synthesis of 6-(indol-3-yl)-1,2'-bipyridin-2-ones S11
4. Same general procedure D for the synthesis of 8-(indol-3-yl)-quinoline *N*-oxides S17
5. Product modifications S19
6. Spectra of new compounds S20

1. General methods

All reagents and solvents were purchased from commercial sources ABCR, Acros, Sigma-Aldrich, Fluka, VWR, Aplichem or Merck and used as such unless stated otherwise. Chromatographic purification was performed as flash chromatography using Macherey-Nagel silica 40–63, 60 Å. TLC was performed on Merck silica gel 60 F254 TLC glass plates and visualized with UV light, permanganate stain or phosphomolybdic acid stain. Melting points were measured on a calibrated Büchi B-540 melting point apparatus using open glass capillaries. ^1H NMR spectra were recorded on a Bruker DPX-400 400 MHz spectrometer in $CDCl_3$, CD_2Cl_2 or DMSO-d_6; all signals are reported in ppm with the internal chloroform signal at 7.26 ppm, the internal CD_2Cl_2 signal at 5.32 ppm or the internal DMSO signal at 2.50 ppm as standard. The data is being reported as (s = singlet d = doublet t = triplet q = quadruplet quint = quintet m = multiplet or unresolved bs = broad signal, coupling constant(s) in Hz, integration, interpretation). ^{19}F NMR spectra were recorded on a Bruker DPX-400 376 MHz spectrometer in $CDCl_3$. ^{13}C NMR spectra were recorded with ^1H-decoupling on a Bruker DPX-400 101 MHz spectrometer in $CDCl_3$, CD_2Cl_2 or DMSO-d_6; all signals are reported in ppm with the internal chloroform signal at 77.2 ppm, the internal CD_2Cl_2 signal at 53.8 ppm or the internal DMSO signal at 39.5 ppm as standard. Infrared spectra were recorded on a JASCO FT-IR B4100 with an ATR device and a ZnSe prisma and are reported as cm^{-1} (w = weak, m = medium, s = strong, br = broad). High-resolution mass spectrometric measurements were performed by the mass spectrometry service of ISIC at the EPFL on a MICROMASS (ESI) Q-TOF Ultima API.

2. Syntheses of starting materials

2. 1. General procedure A for the synthesis of pyridinones

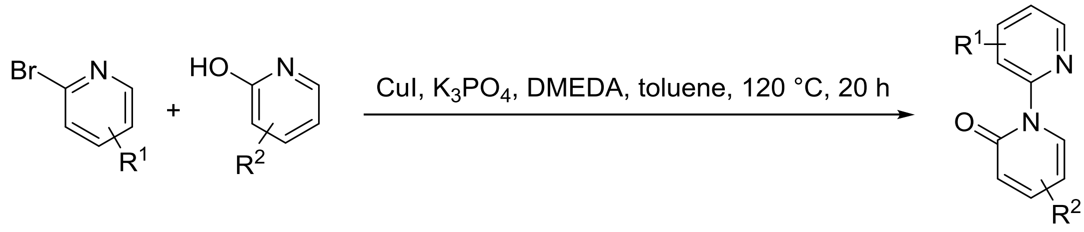

Following a reported procedure,[1] copper iodide (5 mol %), potassium phosphate tribasic (2.00 equiv), the corresponding 2-hydroxypyridine (1.00 equiv) and the corresponding 2-bromopyridine (2.00 equiv) were suspended in toluene [0.4 M] under N_2. *N*,*N*'-Dimethylethylenediamine (0.10 equiv) was added and the resulting mixture was stirred 20 h at 120 °C. The resulting mixture was allowed to cool to rt and then quenched with water. A small amount of *N*,*N*'-dimethylethylenediamine was added to dissolve the residual copper salts into the aqueous phase. The layers were separated and the aqueous layer was extracted three times with EtOAc (20 mL). The organic layers were combined, dried over $MgSO_4$ and concentrated under reduced pressure. A purification by flash chromatography (eluent DCM/EtOAc 1:1 with 4%vv of NEt_3) affordeded the desired product.

2*H*-[1,2'-Bipyridin]-2-one (5a)

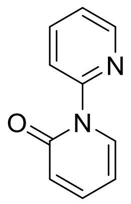

Following general procedure **A** and starting from commercially available 2-hydroxypyridine (1.43 g, 15.0 mmol) and 2-bromopyridine (2.86 mL, 30.0 mmol), **5a** (2.46 g, 14.3 mmol, 95%) (CAS number 3480-65-7) was obtained as a pale yellow solid.
^{1}H NMR (400 MHz, $CDCl_3$) δ 8.56 (d, *J* = 4.7 Hz, 1H, $\underline{H}_{Ar}$), 7.94 (d, *J* = 8.2 Hz, 1H, $\underline{H}_{Ar}$), 7.85 (ddd, *J* = 13.9, 7.4, 4.0 Hz, 2H, $\underline{H}_{Ar}$), 7.38 (ddd, *J* = 8.9, 6.5, 2.0 Hz, 1H, $\underline{H}_{Ar}$), 7.33-7.29 (m, 1H, $\underline{H}_{Ar}$), 6.64 (d, *J* = 9.2 Hz, 1H, $\underline{H}_{Ar}$), 6.29 (t, *J* = 6.8 Hz, 1H, $\underline{H}_{Ar}$).
^{13}C NMR (101 MHz, $CDCl_3$) δ 162.3, 152.0, 149.0, 140.3, 137.9, 136.2, 123.3, 122.2, 121.6, 106.4. Spectra data matched with the values reported in literature.[2]
HRMS calculated for $C_{10}H_8N_2NaO^+$ $[M+Na]^+$ 195.0529; found 195.0535.

5'-Methoxy-2*H*-[1,2'-bipyridin]-2-one (5b)

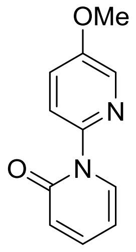

Following general procedure **A** and starting from commercially available 2-hydroxypyridine (143 mg, 1.50 mmol) and 2-bromo-5-methoxypyridine (564 mg, 3.00 mmol), **5b** (231 mg, 1.14 mmol, 76%) (CAS number 10201-69-1) was obtained as a white solid.[3]
^{1}H NMR (400 MHz, $CDCl_3$) δ 8.19 (d, *J* = 3.0 Hz, 1H, $\underline{H}_{Ar}$), 7.80 (d, *J* = 8.9 Hz, 1H, $\underline{H}_{Ar}$), 7.76 (dd, *J* = 7.0, 1.9 Hz, 1H, $\underline{H}_{Ar}$), 7.40-7.29 (m, 2H, $\underline{H}_{Ar}$), 6.61 (d, *J* = 9.2 Hz, 1H, $\underline{H}_{Ar}$), 6.26 (td, *J* = 6.9, 1.2 Hz, 1H, $\underline{H}_{Ar}$), 3.88 (s, 3H, $OC\underline{H}_3$).
^{13}C NMR (101 MHz, $CDCl_3$) δ 162.4, 155.4, 145.0, 140.1, 136.5, 135.8, 122.4, 122.0, 121.9, 106.2, 56.1.
HRMS calculated for $C_{11}H_{11}N_2O_2^+$ $[M+H]^+$ 203.0815; Found 203.0810.

[1] Odani, R.; Hirano, K.; Satoh, T.; Miura, M. *Angew. Chem. Int. Ed.* **2014**, *53*, 10784–10788.
[2] Londregan, A. T.; Jennings, S.; Wei, L. *Org. Lett.* **2011**, *13*, 1840–1843.
[3] Pyridones **5b-d**, **5e-m** and **5p** are known compounds, however no characterization has been reported. See Ref 1.

5'-(Trifluoromethyl)-2*H*-[1,2'-bipyridin]-2-one (5c)

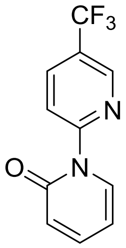

Following general procedure **A** and starting from commercially available 2-hydroxypyridine (143 mg, 1.50 mmol) and 2-bromo-5-(trifluoromethyl)pyridine (678 mg, 3.00 mmol), **5c** (245 mg, 1.02 mmol, 68%) (CAS number 1845694-34-9) was obtained as a white solid.[3]
^{1}H NMR (400 MHz, $CDCl_3$) δ 8.81 (s, 1H, $\underline{H}_{Ar}$), 8.21 (d, *J* = 8.6 Hz, 1H, $\underline{H}_{Ar}$), 8.05 (dd, *J* = 8.6, 2.2 Hz, 1H, $\underline{H}_{Ar}$), 7.96 (dd, *J* = 7.2, 1.5 Hz, 1H, $\underline{H}_{Ar}$), 7.40 (ddd, *J* = 8.7, 6.5, 2.1 Hz, 1H, $\underline{H}_{Ar}$), 6.64 (d, *J* = 9.3 Hz, 1H, $\underline{H}_{Ar}$), 6.32 (td, *J* = 7.2, 1.3 Hz, 1H, $\underline{H}_{Ar}$).
^{19}F NMR (376 MHz, $CDCl_3$) δ -62.3.
^{13}C NMR (101 MHz, $CDCl_3$) δ 162.2, 154.3, 146.0 (q, *J* = 4.2 Hz), 140.7, 135.3, 135.2 (q, *J* = 3.3 Hz), 125.9 (q, *J* = 33.5 Hz), 123.2 (q, *J* = 272.3 Hz), 122.5, 121.2, 106.9.
HRMS calculated for $C_{11}H_8F_3N_2O^+$ $[M+H]^+$ 241.0583; Found 241.0581.

5'-Nitro-2*H*-[1,2'-bipyridin]-2-one (5d)

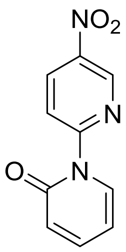

Following general procedure **A** and starting from commercially available 2-hydroxypyridine (143 mg, 1.50 mmol) and 2-bromo-5-nitropyridine (609 mg, 3.00 mmol), **5d** (189 mg, 0.87 mmol, 58%) (CAS number 10201-88-4) was obtained as yellow solid.[3]
^{1}H NMR (400 MHz, $CDCl_3$) δ 9.38 (d, *J* = 2.6 Hz, 1H, $\underline{H}_{Ar}$), 8.60 (dd, *J* = 9.0, 2.7 Hz, 1H, $\underline{H}_{Ar}$), 8.40 (d, *J* = 9.0 Hz, 1H, $\underline{H}_{Ar}$), 8.07 (dd, *J* = 7.3, 1.5 Hz, 1H, $\underline{H}_{Ar}$), 7.43 (ddd, *J* = 8.7, 6.5, 2.0 Hz, 1H, $\underline{H}_{Ar}$), 6.67 (d, *J* = 9.3 Hz, 1H, $\underline{H}_{Ar}$), 6.38-6.34 (m, 1H, $\underline{H}_{Ar}$).
^{13}C NMR (101 MHz, $CDCl_3$) δ 162.1, 155.3, 144.5, 142.9, 140.8, 134.9, 133.1, 122.6, 121.2, 107.2.
HRMS calculated for $C_{10}H_8N_3O_3^+$ $[M+H]^+$ 218.0560; Found 218.0559.

1-(Pyrimidin-2-yl)pyridin-4(1*H*)-one (5e)

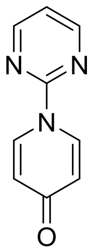

2-chloropyrimidine (172 mg, 1.50 mmol), 4-hydroxypyridine (285 mg, 3.00 mmol), K_2CO_3 (415 mg, 3.00 mmol) were solubilized in water (2 mL) and heated at 90 °C for 30 min. After cooling at rt, the precipitate was filtered off and dried under vacuum to give **5e** (159 mg, 0.918 mmol, 61%) (CAS number 29049-26-1) as a white solid.
^{1}H NMR (400 MHz, CD_2Cl_2) δ 8.83 (d, *J* = 8.2 Hz, 2H), 8.73 (d, *J* = 4.8 Hz, 2H), 7.26 (t, *J* = 4.8 Hz, 1H), 6.36 (d, *J* = 8.2 Hz, 2H).
^{13}C NMR (101 MHz, CD_2Cl_2) δ 180.5, 159.2, 156.0, 134.9, 119.1, 118.7.
HRMS calculated for $C_9H_8N_3O^+$ $[M+H]^+$ 174.0662; Found 174.0661.

1-(Quinolin-2-yl)pyridin-2(1*H*)-one (5f)

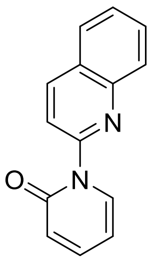

Following general procedure **A** and starting from commercially available 2-hydroxypyridine (152 mg, 1.60 mmol) and 2-bromoquinoline (666 mg, 3.20 mmol), **5f** (342 mg, 1.54 mmol, 96%) (CAS number 10168-48-6) was obtained as a white solid.[3]
^{1}H NMR (400 MHz, $CDCl_3$) δ 8.27 (d, *J* = 8.7 Hz, 1H, $\underline{H}_{Ar}$), 8.08 (d, *J* = 8.4 Hz, 1H, $\underline{H}_{Ar}$), 8.01 (t, *J* = 8.1 Hz, 2H, $\underline{H}_{Ar}$), 7.89 (d, *J* = 8.1 Hz, 1H, $\underline{H}_{Ar}$), 7.76 (t, *J* = 7.7 Hz, 1H, $\underline{H}_{Ar}$), 7.61 (t, *J* = 7.5 Hz, 1H, $\underline{H}_{Ar}$), 7.44 (ddd, *J* = 8.9, 6.5, 1.9 Hz, 1H, $\underline{H}_{Ar}$), 6.69 (d, *J* = 9.3 Hz, 1H, $\underline{H}_{Ar}$), 6.36 (t, *J* = 6.8 Hz, 1H, $\underline{H}_{Ar}$).
^{13}C NMR (101 MHz, $CDCl_3$) δ 162.7, 152.0, 147.3, 140.6, 137.9, 136.5, 130.2, 129.1, 127.7, 127.7, 127.4, 122.4, 119.6, 106.7.
HRMS calculated for $C_{14}H_{11}N_2O^+$ $[M+H]^+$ 223.0866; found 223.0872.

3-Methyl-2*H*-[1,2'-bipyridin]-2-one (5g)

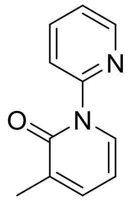

Following general procedure **A** and starting from commercially available 2-hydroxy-3-methylpyridine (458 mg, 4.20 mmol) and 2-bromopyridine (0.80 mL, 8.4 mmol), **5g** (678 mg, 3.64 mmol, 87%) (CAS number 1644063-32-0) was obtained as a colorless oil.[3]

^{1}H NMR (400 MHz, $CDCl_3$) δ 8.59-8.54 (m, 1H, $\underline{H}_{Ar}$), 7.95-7.91 (m, 1H, $\underline{H}_{Ar}$), 7.85-7.79 (m, 1H, $\underline{H}_{Ar}$), 7.73 (d, *J* = 7.1 Hz, 1H, $\underline{H}_{Ar}$), 7.33-7.26 (m, 2H, $\underline{H}_{Ar}$), 6.22 (t, *J* = 6.9 Hz, 1H, $\underline{H}_{Ar}$), 2.19 (s, 3H, $C\underline{H}_3$).

^{13}C NMR (101 MHz, $CDCl_3$) δ 162.7, 152.4, 148.9, 137.7, 137.3, 133.6, 130.9, 123.1, 121.7, 106.1, 17.4.

HRMS calculated for $C_{11}H_{11}N_2O^+$ $[M+H]^+$ 187.0866; found 187.0866.

3-(Trifluoromethyl)-2*H*-[1,2'-bipyridin]-2-one (5h)

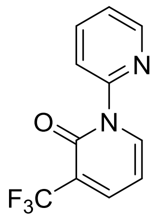

Following general procedure **A** and starting from commercially available 2-hydroxy-3-(trifluoromethyl)-pyridine (326 mg, 2.00 mmol) and 2-bromopyridine (0.38 mL, 4.0 mmol), **5h** (252 mg, 1.05 mmol, 52%) (CAS number 1644063-33-1) was obtained as a white solid.[3]

^{1}H NMR (400 MHz, $CDCl_3$) δ 8.56 (dd, *J* = 4.9, 1.7 Hz, 1H), 8.12 (dd, *J* = 7.1, 1.9 Hz, 1H), 7.98 (d, *J* = 8.2 Hz, 1H), 7.88-7.79 (m, 2H), 7.35 (ddd, *J* = 7.4, 4.9, 0.9 Hz, 1H), 6.37 (t, *J* = 7.0 Hz, 1H).

^{19}F NMR (376 MHz, $CDCl_3$) δ -66.0.

^{13}C NMR (101 MHz, $CDCl_3$) δ 158.1, 150.7, 149.0, 140.2, 139.7 (q, *J* = 4.9 Hz), 137.9, 123.8, 122.6 (q, *J* = 271.8 Hz), 121.8 (q, *J* = 30.9 Hz), 121.5, 104.4.

HRMS calculated for $C_{11}H_8F_3N_2O^+$ $[M+H]^+$ 241.0583; Found 241.0584.

3-Fluoro-2*H*-[1,2'-bipyridin]-2-one (5i)

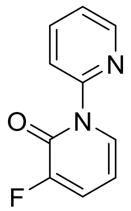

Following general procedure **A** and starting from commercially available 3-fluro-2-hydroxypyridine (226 mg, 2.00 mmol) and 2-bromopyridine (0.38 mL, 4.0 mmol), **5i** (281 mg, 1.48 mmol, 74%) (CAS number 1872255-08-7) was obtained as a white solid.[3]

^{1}H NMR (400 MHz, $CDCl_3$) δ 8.56 (ddd, *J* = 4.9, 1.9, 0.8 Hz, 1H, $\underline{H}_{Ar}$), 7.97 (dt, *J* = 8.2, 0.9 Hz, 1H, $\underline{H}_{Ar}$), 7.85 (ddd, *J* = 8.2, 7.5, 1.9 Hz, 1H, $\underline{H}_{Ar}$), 7.74 (dt, *J* = 7.2, 1.7 Hz, 1H, $\underline{H}_{Ar}$), 7.34 (ddd, *J* = 7.4, 4.9, 1.0 Hz, 1H, $\underline{H}_{Ar}$), 7.15 (ddd, *J* = 9.2, 7.4, 1.8 Hz, 1H, $\underline{H}_{Ar}$), 6.22 (td, *J* = 7.3, 4.5 Hz, 1H, $\underline{H}_{Ar}$).

^{19}F NMR (376 MHz, $CDCl_3$) δ -130.1.

^{13}C NMR (101 MHz, $CDCl_3$) δ 156.2 (d, *J* = 26.5 Hz), 152.8 (d, *J* = 249.9 Hz), 151.1, 149.1, 138.1, 131.4 (d, *J* = 5.3 Hz), 123.7, 121.4, 120.4 (d, *J* = 17.3 Hz), 104.1 (d, *J* = 5.9 Hz).

HRMS calculated for $C_{10}H_8FN_2O^+$ $[M+H]^+$ 191.0615; Found 191.0618.

4-Methyl-2*H*-[1,2'-bipyridin]-2-one (5j)

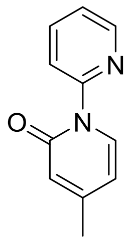

Following general procedure **A** and starting from commercially available 2-hydroxy-4-methylpyridine (458 mg, 4.20 mmol) and 2-bromopyridine (0.80 mL, 8.4 mmol), **5j** (764 mg, 4.10 mmol, 98%) (CAS number 1644063-34-2) was obtained as an off-white solid.[3]

^{1}H NMR (400 MHz, $CDCl_3$) δ 8.54 (d, *J* = 4.9 Hz, 1H, $\underline{H}_{Ar}$), 7.95 (d, *J* = 8.2 Hz, 1H, $\underline{H}_{Ar}$), 7.84-7.76 (m, 2H, $\underline{H}_{Ar}$), 7.29 (ddd, *J* = 7.3, 4.9, 0.9 Hz, 1H, $\underline{H}_{Ar}$), 6.44 (s, 1H, $\underline{H}_{Ar}$), 6.14 (dd, *J* = 7.2, 1.8 Hz, 1H, $\underline{H}_{Ar}$), 2.22 (s, 3H, $C\underline{H}_3$).

^{13}C NMR (101 MHz, $CDCl_3$) δ 162.2, 152.1, 152.0, 148.9, 137.8, 135.0, 123.1, 121.5, 120.2, 109.2, 21.5.

HRMS calculated for $C_{11}H_{11}N_2O^+$ $[M+H]^+$ 187.0866; found 187.0866.

4-(Trifluoromethyl)-2*H*-[1,2'-bipyridin]-2-one (5k)

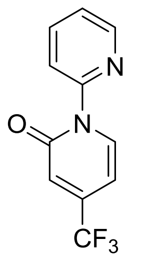

Following general procedure **A** and starting from commercially available 2-hydroxy-3-(trifluoromethyl)-pyridine (245 mg, 1.50 mmol) and 2-bromopyridine (0.29 mL, 3.0 mmol), **5k** (203 mg, 0.850 mmol, 57%) (CAS number 1644063-35-3) was obtained as a pale yellow solid.[3]
^{1}H NMR (400 MHz, $CDCl_3$) δ 8.57 (ddd, *J* = 4.9, 1.8, 0.8 Hz, 1H, $\underline{H}_{Ar}$), 8.06 (d, *J* = 7.4 Hz, 1H, $\underline{H}_{Ar}$), 7.95 (dt, *J* = 8.2, 0.9 Hz, 1H, $\underline{H}_{Ar}$), 7.85 (ddd, *J* = 8.2, 7.5, 1.9 Hz, 1H, $\underline{H}_{Ar}$), 7.35 (ddd, *J* = 7.4, 4.9, 1.0 Hz, 1H, $\underline{H}_{Ar}$), 6.93-6.91 (m, 1H, $\underline{H}_{Ar}$), 6.41 (dd, *J* = 7.4, 2.0 Hz, 1H, $\underline{H}_{Ar}$).
^{19}F NMR (376 MHz, $CDCl_3$) δ -67.0.
^{13}C NMR (101 MHz, $CDCl_3$) δ 161.1, 151.0, 149.2, 141.7 (q, *J* = 34.0 Hz), 138.1, 138.0, 123.8, 122.1 (q, *J* = 274.0 Hz), 121.2, 119.9 (q, *J* = 4.4 Hz), 101.6 (q, *J* = 2.4 Hz).
HRMS calculated for $C_{11}H_8F_3N_2O^+$ $[M+H]^+$ 241.0583; Found 241.0582.

4-(Benzyloxy)-2*H*-[1,2'-bipyridin]-2-one (5l)

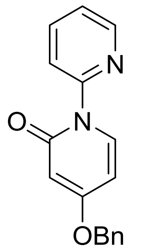

Following general procedure **A** and starting from commercially available 4-benzyloxy-2-hydroxypyridine (302 g, 1.50 mmol) and 2-bromopyridine (0.29 mL, 3.0 mmol), **5l** (113 mg, 0.410 mmol, 27%) (CAS number 1644063-36-4) was obtained as a white solid.[3]
^{1}H NMR (400 MHz, $CDCl_3$) δ 8.54 (d, *J* = 3.9 Hz, 1H, $\underline{H}_{Ar}$), 7.94 (d, *J* = 8.2 Hz, 1H, $\underline{H}_{Ar}$), 7.86-7.78 (m, 2H, $\underline{H}_{Ar}$), 7.403-7.33 (m, 5H, $\underline{H}_{Ar}$), 7.30-7.27 (m, 1H, $\underline{H}_{Ar}$), 6.11 (dd, *J* = 7.8, 2.6 Hz, 1H, $\underline{H}_{Ar}$), 6.03 (d, *J* = 2.5 Hz, 1H, $\underline{H}_{Ar}$), 5.05 (s, 2H, $OC\underline{H}_2$).
^{13}C NMR (101 MHz, $CDCl_3$) δ 167.7, 163.7, 151.8, 148.9, 137.8, 136.2, 135.3, 128.9 (2C), 128.7, 127.9 (2C), 122.9, 121.5, 102.2, 98.6, 70.5.
HRMS calculated for $C_{17}H_{14}N_2NaO_2^+$ $[M+Na]^+$ 301.0953; Found 301.0948.

5-Methyl-2*H*-[1,2'-bipyridin]-2-one (5m)

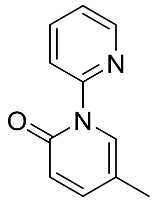

Following general procedure **A** and starting from commercially available 2-hydroxy-4-methylpyridine (458 mg, 4.20 mmol) and 2-bromopyridine (0.80 mL, 8.4 mmol), **5m** (691 mg, 3.71 mmol, 88%) (CAS number 53427-88-6) was obtained as a white solid.[3]
^{1}H NMR (400 MHz, $CDCl_3$) δ 8.55 (d, *J* = 3.9 Hz, 1H, $\underline{H}_{Ar}$), 7.93 (d, *J* = 8.1 Hz, 1H, $\underline{H}_{Ar}$), 7.85-7.78 (m, 1H, $\underline{H}_{Ar}$), 7.64 (s, 1H, $\underline{H}_{Ar}$), 7.32-7.22 (m, 2H, $\underline{H}_{Ar}$), 6.58 (d, *J* = 9.3 Hz, 1H, $\underline{H}_{Ar}$), 2.12 (s, 3H, $C\underline{H}_3$).
^{13}C NMR (101 MHz, $CDCl_3$) δ 161.6, 152.1, 148.9, 143.1, 137.7, 133.2, 123.0, 121.7, 121.6, 115.3, 17.3.
HRMS calculated for $C_{11}H_{11}N_2O^+$ $[M+H]^+$ 187.0866; found 187.0867.

2-(Pyridin-2-yl)isoquinolin-1(2*H*)-one (5n)

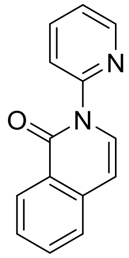

Following general procedure **A** and starting from commercially available isoquinolin-1(2*H*)-one (218 mg, 1.50 mmol) and 2-bromopyridine (0.29 mL, 3.0 mmol), **5n** (276 mg, 1.24 mmol, 83%) (CAS number 1532-89-4) was obtained as a white solid.
^{1}H NMR (400 MHz, $CDCl_3$) δ 8.59 (dd, *J* = 4.8, 1.7 Hz, 1H), 8.49 (d, *J* = 8.0 Hz, 1H), 8.01 (d, *J* = 8.2 Hz, 1H), 7.86 (td, *J* = 7.8, 1.9 Hz, 1H), 7.78 (d, *J* = 7.6 Hz, 1H), 7.69 (td, *J* = 8.2, 7.8, 1.3 Hz, 1H), 7.58-7.49 (m, 2H), 7.31 (ddd, *J* = 7.3, 4.9, 0.9 Hz, 1H), 6.63 (d, *J* = 7.6 Hz, 1H).
^{13}C NMR (101 MHz, $CDCl_3$) δ 162.1, 152.4, 148.9, 137.7, 137.2, 133.0, 130.1, 128.5, 127.2, 126.7, 126.1, 122.7, 121.7, 106.9. Spectra data matched with the values reported in literature.[2]
HRMS calculated for $C_{14}H_{10}N_2NaO^+$ $[M+Na]^+$ 245.0685; Found 245.0694.

4*H*-[1,2'-bipyridin]-4-one (5o)

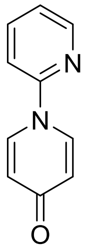

Following general procedure **A** and starting from commercially available 4-hydroxypyridine (143 mg, 1.50 mmol) and 2-bromopyridine (0.29 mL, 3.0 mmol), **5o** (181 mg, 1.05 mmol, 70%) (CAS number 76520-27-9) was obtained as a yellow solid.

^{1}H NMR (400 MHz, $CDCl_3$) δ 8.49 (d, *J* = 4.8 Hz, 1H, $\underline{H}_{Ar}$), 8.18 (d, *J* = 8.0 Hz, 2H, $\underline{H}_{Ar}$), 7.89 (ddd, *J* = 15.7, 8.2, 1.9 Hz, 1H, $\underline{H}_{Ar}$), 7.38 (d, *J* = 8.3 Hz, 1H, $\underline{H}_{Ar}$), 7.30 (dd, *J* = 7.4, 4.8 Hz, 1H, $\underline{H}_{Ar}$), 6.45 (d, *J* = 8.0 Hz, 2H, $\underline{H}_{Ar}$).

^{13}C NMR (101 MHz, $CDCl_3$) δ 180.0, 152.1, 149.2, 139.7, 136.1 (2C), 122.6, 118.9 (2C), 113.0. Spectra data matched with the values reported in literature.[2]

HRMS calculated for $C_{10}H_9N_2O^+$ $[M+H]^+$ 173.0709; Found 173.0713.

1-(Pyridin-2-yl)pyrazin-2(1*H*)-one (5p)

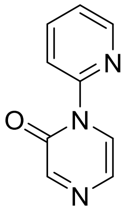

Following general procedure **A** and starting from commercially available 2-hydroxy-4-methylpyridine (192 mg, 2.00 mmol) and 2-bromopyridine (0.38 mL, 4.0 mmol), **5p** (335 mg, 1.93 mmol, 97%) was obtained as a white solid. **mp** 124-126 °C. **Rf** 0.80 (DCM/MeOH 19:1).[3]

^{1}H NMR (400 MHz, $CDCl_3$) δ 8.58 (dd, *J* = 4.9, 1.0 Hz, 1H, $\underline{H}_{Ar}$), 8.25 (d, *J* = 0.9 Hz, 1H, $\underline{H}_{Ar}$), 8.16 (d, *J* = 8.2 Hz, 1H, $\underline{H}_{Ar}$), 7.94 (dd, *J* = 4.7, 1.0 Hz, 1H, $\underline{H}_{Ar}$), 7.87 (td, *J* = 7.9, 1.8 Hz, 1H, $\underline{H}_{Ar}$), 7.42 (d, *J* = 4.7 Hz, 1H, $\underline{H}_{Ar}$), 7.36 (ddd, *J* = 7.4, 4.9, 0.6 Hz, 1H, $\underline{H}_{Ar}$).

^{13}C NMR (101 MHz, $CDCl_3$) δ 155.5, 151.5, 149.9, 149.1, 138.3, 125.3, 123.9, 123.9, 120.4.

IR 2994 (w), 1679 (s), 1587 (s), 1569 (s), 1502 (s), 1470 (m), 1443 (s), 1287 (s), 1264 (s), 1250 (s), 1216 (s), 1156 (m), 1034 (m), 1020 (m), 995 (m), 851 (m), 798 (s), 782 (s), 736 (s).

HRMS calculated for $C_9H_8N_3O^+$ $[M+H]^+$ 174.0662; found 174.0662.

2.2. General procedure B for the synthesis of *N*-alkylindoles

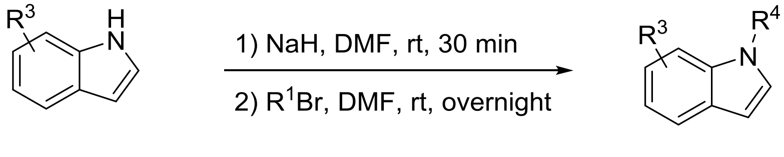

Sodium hydride (60% in mineral oil, 1.10 equiv) was suspended in DMF [0.6 M]. 1H-Indole (1.00 equiv) was solubilized in DMF [1.0 M] and added to the suspension at 0 °C. The mixture was stirred at rt for 30 min. Bromoalkyl or TMSCl (1.50 equiv) was diluted in DMF [3.0 M] and added to the solution at 0 °C. The mixture was stirred at rt for 1 hour. The solution was quenched with water (20 mL) and extracted three times with EtOAc (10 mL). The organic layers were combined, dried over $MgSO_4$ and concentrated under reduced pressure. The liquid was filtered through a 5 cm pad of silica with 100% pentane or Et_2O to afforded the title compound.

5-Bromo-1-methyl-1*H*-indole (15b)

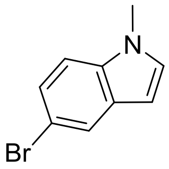

Following general procedure **B** and starting from commercially available 5-bromoindole (0.59 mg, 3.0 mmol), **15b** (630 mg, 3.00 mmol, quantitative yield) (CAS number 10075-52-2) was obtained as a pale yellow solid.

^{1}H NMR (400 MHz, $CDCl_3$) δ 7.75 (d, *J* = 1.6 Hz, 1H, $\underline{H}_{Ar}$), 7.30 (dd, *J* = 8.7, 1.8 Hz, 1H, $\underline{H}_{Ar}$), 7.19 (d, *J* = 8.7 Hz, 1H, $\underline{H}_{Ar}$), 7.05 (d, *J* = 3.1 Hz, 1H, $\underline{H}_{Ar}$), 6.43 (d, *J* = 3.1 Hz, 1H, $\underline{H}_{Ar}$), 3.78 (s, 3H, $NC\underline{H}_3$).

^{13}C NMR (101 MHz, $CDCl_3$) δ 135.5, 130.2, 130.1, 124.4, 123.4, 112.8, 110.8, 100.6, 33.1. Spectra data matched with the values reported in literature.[4]

1-(*tert*-Butyldimethylsilyl)-1*H*-indole (15c)

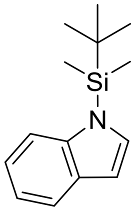

Following general procedure **B** and starting from commercially available indole (2.34 g, 20.0 mmol), **15c** (4.63 g, 20.0 mmol, quantitative yield) (CAS number 40899-73-8) was obtained as a pale yellow solid.

^{1}H NMR (400 MHz, $CDCl_3$) δ 7.56 (d, *J* = 7.2 Hz, 1H, $\underline{H}_{Ar}$), 7.44 (d, *J* = 7.7 Hz, 1H, $\underline{H}_{Ar}$), 7.10 (d, *J* = 3.2 Hz, 1H, $\underline{H}_{Ar}$), 7.09-6.99 (m, 2H, $\underline{H}_{Ar}$), 6.54 (d, *J* = 2.7 Hz, 1H, $\underline{H}_{Ar}$), 0.85 (s, 9H, $C(C\underline{H}_3)_3$), 0.52 (s, 6H, $Si(C\underline{H}_3)_2$).

^{13}C NMR (101 MHz, $CDCl_3$) δ 141.1, 131.5, 131.1, 121.5, 120.8, 119.9, 114.0, 104.9, 26.5, 19.7, -3.8. Spectra data matched with the values reported in literature.[5]

6-Bromo-1-(*tert*-butyldimethylsilyl)-1*H*-indole (15d)

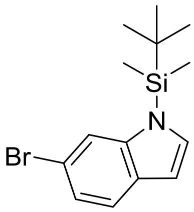

Following general procedure **B** and starting from commercially available indole (2.34 g, 20.0 mmol), **Xd** (4.63 g, 20.0 mmol, quantitative yield) (CAS number 40899-73-8) was obtained as a pale yellow solid.

^{1}H NMR (400 MHz, $CDCl_3$) δ 7.64 (s, 1H, $\underline{H}_{Ar}$), 7.48 (d, $\underline{J}$ = 8.4 Hz, 1H, $\underline{H}_{Ar}$), 7.21 (dd, *J* = 8.4, 1.7 Hz, 1H, $\underline{H}_{Ar}$), 7.15 (d, *J* = 3.2 Hz, 1H, $\underline{H}_{Ar}$), 6.58 (dd, *J* = 3.2, 0.8 Hz, 1H, $\underline{H}_{Ar}$), 0.93 (s, 9H, $C(C\underline{H}_3)_3$), 0.61 (s, 6H, $Si(C\underline{H}_3)_2$).

^{13}C NMR (101 MHz, $CDCl_3$) δ 142.1, 131.9, 130.4, 123.3, 121.9, 116.9, 115.2, 105.1, 26.5, 19.7, -3.7. Spectra data matched with the values reported in literature.[6]

---

[4] Greulich, T.W.; Daniliuc, C.*J*.; Studer, A. *Org. Lett.* **2015**, *17*, 254−257.
[5] Dhanak, D.; Reese, C. B. *J. Chem. Soc., Perkin Trans. 1* **1986**, 2181–2186.
[6] Kawasaki, I.; Yamashita, M; Ohta, S. *Chem. Pharm. Bull.* **1996**, *44*, 1831–1839.

2.3. General procedure C for the synthesis of indoleBX reagents

1-Acetoxy-1,2-benziodoxol-3-(1*H*)-one (18)

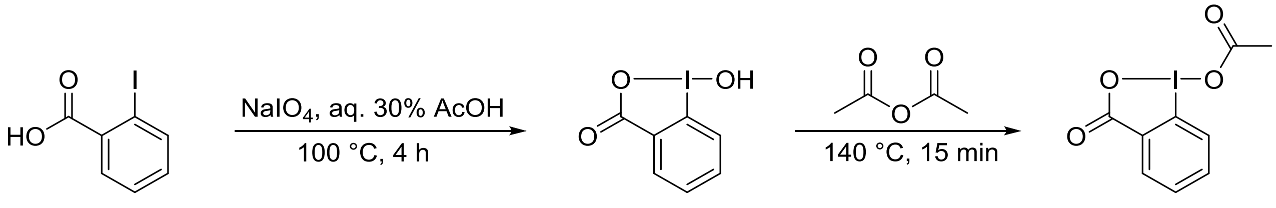

Following a reported procedure[7], sodium periodate (18.1 g, 85.0 mmol, 1.05 equiv) and 2-iodobenzoic acid (20.0 g, 81.0 mmol, 1.00 equiv) were suspended in 30% (v:v) aq. AcOH (160 mL). The mixture was vigorously stirred and refluxed for 4 h and allowed to cool to room temperature, while protecting it from light. After 1 h, the crude product was collected by filtration. The crystals were washed with ice water (3 × 40 mL) followed by acetone (45 mL) and dried under reduced pressure in the dark to afforded 1-hydroxy-1,2-benziodoxol-3-(1*H*)-one (20.8 g, 79.0 mmol, 98%) as a white solid. Following a reported procedure, 1-hydroxy-1,2-benziodoxol-3-(1*H*)-one (20.8 g, 79.0 mmol, 1.00 equiv) was suspended in acetic anhydride (75.0 mL, 788 mmol, 10.0 equiv) and heated to reflux (140 °C) until complete dissolution (about 15 min). The resulting clears solution was slowly let to cool to room temperature and then cooled to 5 °C in the fridge. The white crystals were filtered, washed with pentane (3 × 30 mL) and dried under reduced pressure to afforded 1-acetoxy-1,2-benziodoxol-3-(1*H*)-one (22.3 g, 73.0 mmol, 92%) as a white solid.

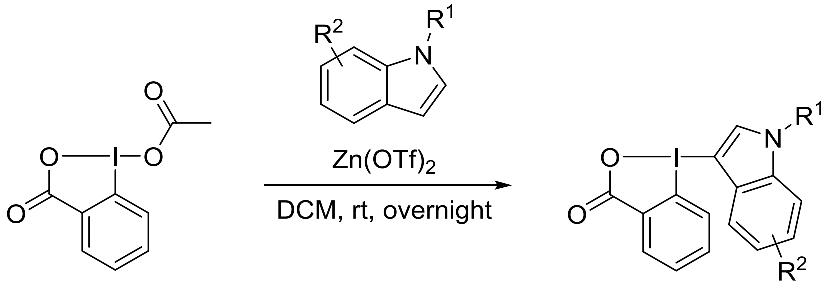

Following a slightly modified reported procedure,[8] 1-acetoxy-1,2- benziodoxol-3-(1*H*)-one (1.20 equiv), the corresponding azaheterocycle (1.00 equiv) and zinc(II) trifluoromethanesulfonate (0.20 equiv) were dissolved in DCM [0.05 M]. The reaction was stirred overnight at room temperature, directly purified by flash chromatography (eluent DCM/MeOH see ratio thereafter) and triturated in ACN to afforded the pure desired hypervalent iodine reagent.

1-(1-Methyl-1*H*-indol-3-yl)-1*H*-1λ$_3$ -benzo[*b*]iodo-3(2*H*)-one (6a)

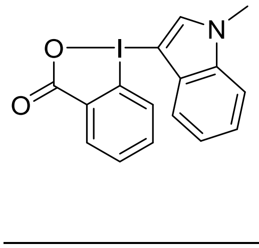

Following general procedure **C**, starting from commercially available 1-methylindole (0.43 mL, 3.4 mmol) and **16**, a purification by column chromatography (DCM/MeOH 19:1) affordeded the title compound **6a** (0.79 g, 2.1 mmol, 61% yield) (CAS number 2130906-04-4) as an off-white solid.

^{1}H NMR (400 MHz, $CDCl_3$) δ 8.43 (d, *J* = 7.5 Hz, 1H, $\underline{H}_{Ar}$), 7.77 (s, 1H, C$\underline{H}$-N), 7.56-7.49 (m, 2H, $\underline{H}_{Ar}$), 7.47-7.41 (m, 2H, $\underline{H}_{Ar}$), 7.35-7.26 (m, 2H, $\underline{H}_{Ar}$), 6.85 (d, *J*

[7] Parsons, A. T.; Buchwald, S. L. *Angew. Chem. Int. Ed.* **2011**, *50*, 9120–9123.
[8] Caramenti, P.; Nicolai, S.; Waser, *J. Chem. - Eur. J.* **2017**, *23*, 14702–14706.

= 8.2 Hz, 1H, H_{Ar}), 4.02 (s, 3H, NCH_3).
^{13}C NMR (101 MHz, $CDCl_3$) δ 167.0, 138.7, 137.7, 133.6, 133.4, 132.7, 130.7, 129.5, 125.4, 124.5, 122.8, 120.1, 116.3, 110.9, 79.2, 34.1. Spectra data matched with the values reported in literature.[7]

1-(5-Bromo-1-methyl-1*H*-indol-3-yl)-1*H*-1λ_3 -benzo[*b*]iodo-3(2*H*)-one (6b)

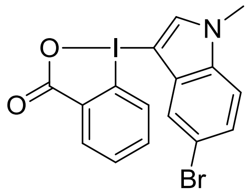

Following general procedure **C**, starting from **15b** (406 mg, 1.93 mmol) and **16**, a purification by column chromatography (DCM/MeOH 19:1) affordeded the title compound **6b** (470 mg, 1.03 mmol, 53% yield) as an off-white solid.
^{1}H NMR (400 MHz, CD_2Cl_2) δ 8.33 (d, *J* = 7.4 Hz, 1H, H_{Ar}), 7.70 (s, 1H, CH-N), 7.63 (s, 1H, H_{Ar}), 7.57 (t, *J* = 7.3 Hz, 1H, H_{Ar}), 7.52 (dd, *J* = 8.8, 1.6 Hz, 1H, H_{Ar}), 7.44 (d, *J* = 8.8 Hz, 1H, H_{Ar}), 7.34 (t, *J* = 7.1 Hz, 1H, H_{Ar}), 6.84 (d, *J* = 8.3 Hz, 1H, H_{Ar}), 3.96 (s, 3H, NCH_3).
^{13}C NMR (101 MHz, CD_2Cl_2) δ 166.6, 139.9, 136.9, 133.9, 133.7, 132.5, 131.5, 131.0, 127.5, 125.7, 123.0, 116.7, 116.2, 112.8, 79.5, 34.4. Spectra data matched with the values reported in literature.[9]

1-(1*H*-Indol-3-yl)-1*H*-1λ3 -benzo[*b*]iodo-3(2*H*)-one (6c)

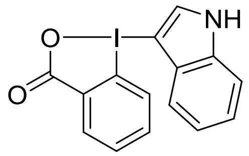

Following general procedure **C** but replacing $Zn(OTf)_2$ by $Sc(OTf)_3$, starting from **15c** (579 mg, 2.50 mmol) and **16**, a purification by column chromatography (DCM/MeOH 5:1) affordeded the title compound **6c** (527 mg, 1.45 mmol, 58% yield) as a pale beige solid.
^{1}H NMR (400 MHz, DMSO-d6) δ 12.36 (s, 1H, NH), 8.26 (s, 1H, CH-N), 8.12 (d, *J* = 7.3 Hz, 1H, H_{Ar}), 7.64 (d, *J* = 8.2 Hz, 1H, H_{Ar}), 7.56 (t, *J* = 7.3 Hz, 1H, H_{Ar}), 7.49 (d, *J* = 7.9 Hz, 1H, H_{Ar}), 7.41 (t, *J* = 7.6 Hz, 1H, H_{Ar}), 7.31 (t, *J* = 7.6 Hz, 1H, H_{Ar}), 7.20 (t, *J* = 7.5 Hz, 1H, H_{Ar}), 6.76 (d, *J* = 8.2 Hz, 1H, H_{Ar}).
^{13}C NMR (101 MHz, DMSO-d6) δ 165.8, 136.5 (2C), 134.6, 133.1, 131.3, 130.1, 128.6, 126.2, 123.3, 121.6, 119.2, 116.0, 112.9, 80.3. Spectra data matched with the values reported in literature.[7]

1-(6-Bromo-1*H*-indol-3-yl)-1*H*-1λ3 -benzo[*b*]iodo-3(2*H*)-one (6d)

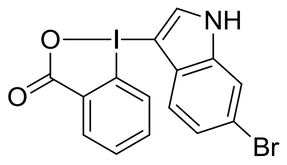

Following general procedure **C**, but replacing $Zn(OTf)_2$ by $Sc(OTf)_3$, starting from **15d** (528 mg, 1.70 mmol) and **16**, a purification by column chromatography (DCM/MeOH 19:1) and washing with DCM affordeded the title compound **6d** (563 mg, 1.27 mmol, 75% yield) as a white solid. **mp 194-196** °C. **Rf** 0.58 (DCM/MeOH 9:1).
^{1}H NMR (400 MHz, DMSO-d6) δ 12.46 (bs, 1H, NH), 8.27 (s, 1H, H_{Ar}), 8.12 (dd, *J* = 7.5, 1.5 Hz, 1H, H_{Ar}), 7.86 (d, *J* = 1.5 Hz, 1H, H_{Ar}), 7.57 (t, *J* = 7.3 Hz, 1H, H_{Ar}), 7.49 (d, *J* = 8.5 Hz, 1H, H_{Ar}), 7.42 (td, *J* = 7.3, 1.5 Hz, 1H, H_{Ar}), 7.32 (dd, *J* = 8.5, 1.7 Hz, 1H, H_{Ar}), 6.74 (d, *J* = 8.1 Hz, 1H, H_{Ar}).
^{13}C NMR (101 MHz, DMSO-d6) δ 165.9, 137.5, 137.4, 134.4, 133.3, 131.4, 130.2, 127.7, 126.3, 124.4, 121.2, 116.1, 116.0, 115.4, 80.9.
IR ν_{max} 2606 (br), 1593 (m), 1578 (m), 1549 (m), 1366 (s), 1265 (m), 887 (m), 828 (s), 794 (s), 736 (s).
HRMS calculated for $C_{15}H_{10}{}^{79}BrINO_2^+$ $[M+H]^+$ 441.8934; found 441.8933.

[9] Grenet, E.; Waser, J. *Org. Lett.* **2018**, *20*, 1473-1475.

3. General procedure D for the synthesis of 6-(indol-3-yl)-1,2'-bipyridin-2-ones

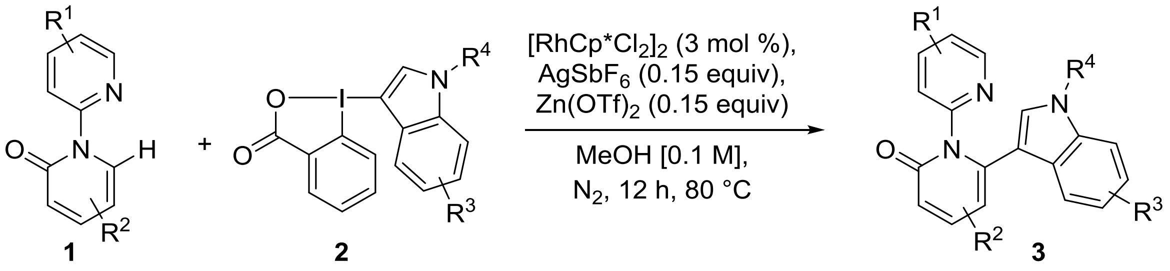

In a sealed tube, $[RhCp^*Cl_2]_2$ (3.7 mg, 6.0 µmol, 3 mol %), $AgSbF_6$ (10.3 mg, 30.0 µmol, 0.15 equiv), $Zn(OTf)_2$ (10.9 mg, 30.0 µmol, 0.15 equiv), the corresponding pyridinone (0.20 mmol, 1.00 equiv) and the corresponding hypervalent iodine reagent (0.20 mmol, 1.00 equiv) were solubilized in dry MeOH (2.0 mL, 0.1 M) under N_2. The mixture was stirred at 80 °C for 12 h. The suspension was diluted with DCM (5 mL) and quenched with a saturated aqueous solution of $NaHCO_3$ (5 mL). The two layers were separated and the aqueous layer was extracted twice with DCM (5 mL). The organic layers were combined, dried over magnesium sulfate dehydrate, filtered and concentrated under reduced pressure. The crude residue was purified by preparative TLC using DCM/MeOH 19:1 as eluent to afforded the pure title compound.

6-(1-Methyl-1*H*-indol-3-yl)-2*H*-[1,2'-bipyridin]-2-one (7a)

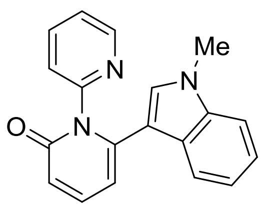

Following General Procedure **D**, starting from **5a** and **6a**, a purification by preparative TLC (DCM/MeOH 19:1) afforded the title compound **7a** (51.9 mg, 0.172 mmol, 86% yield) as a white solid. **mp** 231-233 °C. **Rf** 0.51 (DCM/MeOH 19:1).

^{1}H NMR (400 MHz, $CDCl_3$) δ 8.58 (ddd, *J* = 4.9, 1.8, 0.7 Hz, 1H, $\underline{H}_{Ar}$), 7.71 (d, *J* = 8.0 Hz, 1H, $\underline{H}_{Ar}$), 7.63 (td, *J* = 7.7, 1.9 Hz, 1H, $\underline{H}_{Ar}$), 7.50 (dd, *J* = 9.2, 7.0 Hz, 1H, $\underline{H}_{Ar}$), 7.25-7.21 (m, 3H, $\underline{H}_{Ar}$), 7.19-7.12 (m, 2H, $\underline{H}_{Ar}$), 6.63 (dd, *J* = 9.2, 1.1 Hz, 1H, $\underline{H}_{Ar}$), 6.50 (dd, *J* = 7.0, 1.1 Hz, 1H, $\underline{H}_{Ar}$), 6.47 (s, 1H, $C2_{indole}\underline{H}$), 3.57 (s, 3H, $NC\underline{H}_3$).

^{13}C NMR (101 MHz, $CDCl_3$) δ 164.2, 153.2, 149.2, 143.2, 140.3, 138.4, 136.3, 129.9, 126.7, 124.2, 123.7, 122.6, 120.6, 119.9, 118.7, 109.6, 109.5, 108.0, 33.0.

IR ν_{max} 3080 (w), 1660 (s), 1591 (s), 1580 (s), 1557 (s), 1437 (m), 1391 (m), 1237 (m), 1142 (m), 807 (s), 786 (m), 750 (s).

HRMS calculated for $C_{19}H_{16}N_3O^+$ $[M+H]^+$ 302.1288; Found 302.1291.

5'-Methoxy-6-(1-methyl-1*H*-indol-3-yl)-2*H*-[1,2'-bipyridin]-2-one (7b)

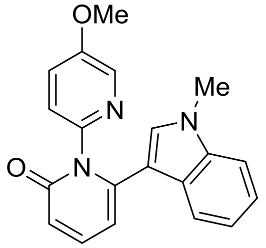

Following General Procedure **D**, starting from **5b** and **6a**, a purification by preparative TLC (DCM/MeOH 19:1) afforded the title compound **7b** (54.3 mg, 0.164 mmol, 82% yield) as a yellow solid. **mp** 225-227 °C. **Rf** 0.51 (DCM/MeOH 19:1).

^{1}H NMR (400 MHz, $CDCl_3$) δ 8.13 (d, *J* = 2.6 Hz, 1H, $\underline{H}_{Ar}$), 7.61 (d, *J* = 8.0 Hz, 1H, $\underline{H}_{Ar}$), 7.37 (dd, *J* = 9.2, 7.0 Hz, 1H, $\underline{H}_{Ar}$), 7.14 (d, *J* = 3.8 Hz, 2H, $\underline{H}_{Ar}$), 7.09-7.00 (m, 2H, $\underline{H}_{Ar}$), 6.95 (d, *J* = 8.7 Hz, 1H, $\underline{H}_{Ar}$), 6.50 (dd, *J* = 9.2, 1.2 Hz, 1H, $\underline{H}_{Ar}$), 6.41 (s, 1H, $C2_{indole}\underline{H}$), 6.37 (dd, *J* = 7.0, 1.2 Hz, 1H, $\underline{H}_{Ar}$), 3.72 (s, 3H, $OC\underline{H}_3$), 3.50 (s, 3H, $NC\underline{H}_3$).

^{13}C NMR (101 MHz, $CDCl_3$) δ 164.5, 155.5, 145.7, 143.6, 140.1, 136.3, 136.2, 130.0, 126.7, 124.2, 122.9, 122.6, 120.6, 119.9, 118.5, 109.6, 109.6, 107.9, 56.0, 33.1.

IR ν_{max} 3080 (w), 1662 (s), 1582 (s), 1543 (s), 1473 (s), 1260 (s), 807 (s), 752 (s), 737 (s).

HRMS calculated for $C_{20}H_{18}N_3O_2^+$ $[M+H]^+$ 332.1394; Found 332.1392.

6-(1-Methyl-1*H*-indol-3-yl)-5'-(trifluoromethyl)-2*H*-[1,2'-bipyridin]-2-one (7c)

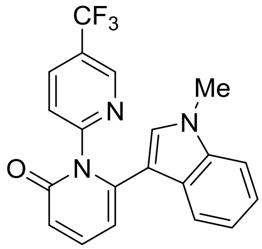

Following General Procedure **D**, starting from **5c** and **6a**, a purification by preparative TLC (DCM/MeOH 19:1) afforded the title compound **7c** (53.2 mg, 0.144 mmol, 72% yield) as a yellow solid. **mp** > 300 °C. **Rf** 0.74 (DCM/MeOH 19:1).

^{1}H NMR (400 MHz, $CDCl_3$) δ 8.82 (s, 1H, $\underline{H}_{Ar}$), 7.88 (d, *J* = 8.2 Hz, 1H, $\underline{H}_{Ar}$), 7.66 (d, *J* = 8.0 Hz, 1H, $\underline{H}_{Ar}$), 7.54 (dd, *J* = 9.2, 7.0 Hz, 1H, $\underline{H}_{Ar}$), 7.34 (d, *J* = 8.2 Hz, 1H, $\underline{H}_{Ar}$), 7.29-7.24 (m, 2H, $\underline{H}_{Ar}$), 7.22-7.11 (m, 1H, $\underline{H}_{Ar}$), 6.67 (d, *J* = 9.2 Hz, 1H, $\underline{H}_{Ar}$), 6.54-6.49 (m, 2H, $\underline{H}_{Ar}$ + $C2_{indole}\underline{H}$), 3.63 (s, 1H, $NC\underline{H}_3$).

^{19}F NMR (376 MHz, $CDCl_3$) δ -62.3.

^{13}C NMR (101 MHz, $CDCl_3$) δ 164.0, 156.1, 146.4 (q, *J* = 3.6 Hz), 142.9, 140.7, 136.4, 135.7 (q, *J* = 3.2 Hz), 129.7, 126.6 (q, *J* = 33.5 Hz), 126.5, 124.4, 123.2 (q, *J* = 272.7 Hz), 122.9, 120.8, 119.7, 118.9, 109.8, 109.3, 108.5, 33.1.

IR ν_{max} 1663 (m), 1583 (m), 1544 (m), 1326 (s), 1128 (s), 1081 (s), 1017 (s), 799 (s), 743 (s).

HRMS calculated for $C_{20}H_{15}F_3N_3O^+$ $[M+H]^+$ 370.1162; Found 370.1160.

6-(1-Methyl-1*H*-indol-3-yl)-1-(quinolin-2-yl)pyridin-2(1*H*)-one (7f)

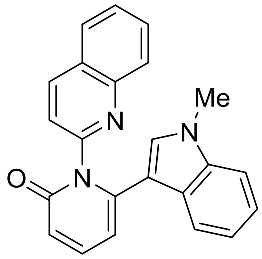

Following General Procedure **D**, starting from **5f** and **6a**, a purification by preparative TLC (DCM/MeOH 19:1) afforded the title compound **7f** (55.4 mg, 0.158 mmol, 79% yield) as a pale yellow solid. **mp** 231-233 °C. **Rf** 0.58 (DCM/MeOH 19:1).

^{1}H NMR (400 MHz, $CDCl_3$) δ 8.12 (d, *J* = 8.6 Hz, 1H, $\underline{H}_{Ar}$), 8.08 (d, *J* = 8.6 Hz, 1H, $\underline{H}_{Ar}$), 7.80-7.71 (m, 3H, $\underline{H}_{Ar}$), 7.59-7.52 (m, 2H, $\underline{H}_{Ar}$), 7.23-7.13 (m, 4H, $\underline{H}_{Ar}$), 6.66 (dd, *J* = 9.2, 1.2 Hz, 1H, $\underline{H}_{Ar}$), 6.55-6.52 (m, 2H, $\underline{H}_{Ar}$ + $C2_{indole}\underline{H}$), 3.39 (s, 3H, $NC\underline{H}_3$).

^{13}C NMR (101 MHz, $CDCl_3$) δ 164.3, 152.5, 147.1, 143.1, 140.4, 138.8, 136.2, 130.4, 130.1, 129.5, 127.8, 127.7, 127.5, 126.9, 122.6, 121.6, 120.7, 119.8, 118.8, 109.6, 109.2, 108.1, 32.9.

IR ν_{max} 3062 (w), 1669 (s), 1586 (s), 1544 (s), 1504 (m), 1398 (m), 1236 (m), 815 (s), 803 (s), 768 (s), 745 (s).

HRMS calculated for $C_{23}H_{17}N_3NaO^+$ $[M+Na]^+$ 374.1264; found 374.1271.

3-Methyl-6-(1-methyl-1*H*-indol-3-yl)-2*H*-[1,2'-bipyridin]-2-one (7g)

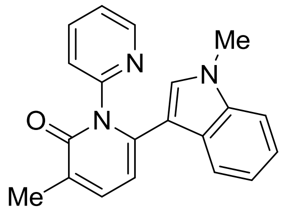

Following General Procedure **D**, starting from **5g** and **6a**, a purification by preparative TLC (DCM/MeOH 19:1) afforded the title compound **7g** (50.5 mg, 0.160 mmol, 80% yield) as a pale yellow solid. **mp** 228-230 °C. **Rf** 0.51 (DCM/MeOH 19:1).

^{1}H NMR (400 MHz, $CDCl_3$) δ 8.57 (dd, *J* = 4.9, 1.1 Hz, 1H, $\underline{H}_{Ar}$), 7.70 (d, *J* = 8.0 Hz, 1H, $\underline{H}_{Ar}$), 7.61 (td, *J* = 7.7, 1.9 Hz, 1H, $\underline{H}_{Ar}$), 7.38 (dd, *J* = 7.0, 1.1 Hz, 1H, $\underline{H}_{Ar}$), 7.24-7.19 (m, 3H, $\underline{H}_{Ar}$), 7.18-7.10 (m, 2H, $\underline{H}_{Ar}$), 6.46 (s, 1H, $C2_{indole}\underline{H}$), 6.43 (d, *J* = 7.0 Hz, 1H, $\underline{H}_{Ar}$), 3.57 (s, 3H, $NC\underline{H}_3$), 2.22 (d, *J* = 0.8 Hz, 3H, $C\underline{H}_3$).

^{13}C NMR (101 MHz, $CDCl_3$) δ 164.4, 153.6, 149.1, 140.2, 138.2, 137.5, 136.3, 129.6, 127.7, 126.8, 124.2, 123.5, 122.5, 120.4, 119.9, 109.8, 109.5, 107.7, 32.9, 17.1.

IR ν_{max} 3087 (w), 2926 (w), 1650 (s), 1603 (m), 1556 (s), 1469 (m), 1243 (m), 1133 (m), 809 (m), 792 (s), 746 (s).

HRMS calculated for $C_{20}H_{18}N_3O^+$ $[M+H]^+$ 316.1444; Found 316.1448.

6-(1-Methyl-1*H*-indol-3-yl)-3-(trifluoromethyl)-2*H*-[1,2'-bipyridin]-2-one (7h)

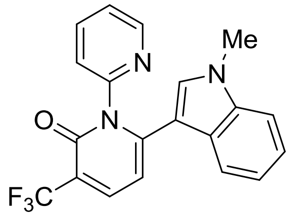

Following General Procedure **D**, starting from **5h** and **6a**, a purification by preparative TLC (DCM/MeOH 19:1) afforded the title compound **7h** (48.0 mg, 0.130 mmol, 65% yield) as an off-white solid. **mp** 265-267 °C. **Rf** 0.71 (DCM/MeOH 19:1).

^{1}H NMR (400 MHz, CD_2Cl_2) δ 8.59 (d, *J* = 3.5 Hz, 1H, $\underline{H}_{Ar}$), 7.89 (d, *J* = 7.5 Hz, 1H, $\underline{H}_{Ar}$), 7.73 (d, *J* = 7.3 Hz, 2H, $\underline{H}_{Ar}$), 7.39-7.09 (m, 5H, $\underline{H}_{Ar}$), 6.60 (d, *J* = 7.5 Hz, 1H, $\underline{H}_{Ar}$), 6.46 (s, 1H, $C2_{indole}\underline{H}$), 3.57 (s, 3H, $NC\underline{H}_3$).

^{19}F NMR (376 MHz, $CDCl_3$) δ -63.6.

^{13}C NMR (101 MHz, CD_2Cl_2) δ 160.1, 152.6, 149.7, 148.6, 139.8 (q, *J* = 4.8 Hz), 138.9, 136.8, 131.2, 126.6, 124.6, 124.4, 123.7 (q, *J* = 270.8 Hz), 123.2, 121.4, 119.8, 117.0 (q, *J* = 30.7 Hz), 110.3, 108.7, 105.9, 33.4.

IR ν_{max} 3105 (w), 1672 (m), 1555 (s), 1416 (m), 1318 (s), 1142 (s), 1117 (s), 1104 (s), 1057 (s), 1038 (s), 739 (s).

HRMS calculated for $C_{20}H_{14}F_3N_3NaO^+$ $[M+Na]^+$ 392.0981; Found 392.0984.

3-Fluoro-6-(1-methyl-1*H*-indol-3-yl)-2*H*-[1,2'-bipyridin]-2-one (7i)

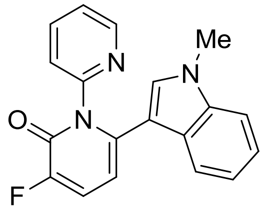

Following General Procedure **D**, starting from **5i** and **6a**, a purification by preparative TLC (DCM/MeOH 19:1) afforded the title compound **7i** (47.9 mg, 0.150 mmol, 75% yield) as an off-white solid. **mp** 204-206 °C. **Rf** 0.58 (DCM/MeOH 19:1).

^{1}H NMR (400 MHz, $CDCl_3$) δ 8.56 (dd, *J* = 4.9, 1.2 Hz, 1H, $\underline{H}_{Ar}$), 7.67-7.62 (m, 2H, $\underline{H}_{Ar}$), 7.30-7.24 (m, 2H, $\underline{H}_{Ar}$), 7.24-7.21 (m, 2H, $\underline{H}_{Ar}$), 7.18-7.11 (m, 2H, $\underline{H}_{Ar}$), 6.50 (s, 1H, $C2_{indole}\underline{H}$), 6.38 (dd, *J* = 7.8, 4.4 Hz, 1H, $\underline{H}_{Ar}$), 3.59 (s, 3H, $NC\underline{H}_3$).

^{19}F NMR (376 MHz, $CDCl_3$) δ -133.9.

^{13}C NMR (101 MHz, $CDCl_3$) δ 157.5 (d, *J* = 26.0 Hz), 152.1 (d, *J* = 1.3 Hz), 151.0 (d, *J* = 248.5 Hz), 149.3, 138.7 (d, *J* = 5.4 Hz), 138.5, 136.2, 129.7, 126.6, 124.0, 123.9, 122.6, 120.9 (d, *J* = 16.5 Hz), 120.6, 119.6, 109.6, 108.9, 105.5 (d, *J* = 5.1 Hz), 33.0.

IR ν_{max} 3056 (w), 1673 (s), 1617 (s), 1570 (m), 1278 (s), 1239 (s), 1223 (m), 1137 (m), 819 (m), 784 (m), 753 (s), 745 (s).

HRMS calculated for $C_{19}H_{15}FN_3O^+$ $[M+H]^+$ 320.1194; Found 320.1192.

4-Methyl-6-(1-methyl-1*H*-indol-3-yl)-2*H*-[1,2'-bipyridin]-2-one (7j)

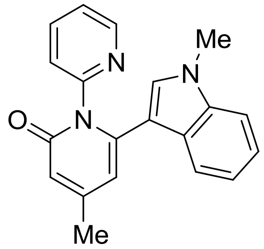

Following General Procedure **D**, starting from **5j** and **6a**, a purification by preparative TLC (DCM/MeOH 19:1) afforded the title compound **7j** (49.2 mg, 0.156 mmol, 78% yield) as an off-white solid. **mp** 186-188 °C. **Rf** 0.41 (DCM/MeOH 19:1).

^{1}H NMR (400 MHz, $CDCl_3$) δ 8.54 (dd, *J* = 4.6, 1.4 Hz, 1H, $\underline{H}_{Ar}$), 7.70 (d, *J* = 8.0 Hz, 1H, $\underline{H}_{Ar}$), 7.60 (td, *J* = 7.7, 1.9 Hz, 1H, $\underline{H}_{Ar}$), 7.24-7.09 (m, 5H, $\underline{H}_{Ar}$), 6.49 (s, 1H, $C2_{indole}\underline{H}$), 6.44 (s, 1H, C(O)C$\underline{H}$), 6.34 (d, *J* = 1.6 Hz, 1H), 3.57 (s, 3H, $NC\underline{H}_3$), 2.28 (s, 3H, $C\underline{H}_3$).

^{13}C NMR (101 MHz, $CDCl_3$) δ 164.2, 153.2, 151.7, 149.1, 141.8, 138.2, 136.3, 129.9, 126.6, 124.3, 123.5, 122.5, 120.5, 119.8, 117.2, 110.6, 109.6, 109.5, 33.0, 21.7.

IR ν_{max} 2970 (m), 2902 (m), 1657 (s), 1599 (s), 1588 (s), 1549 (s), 1470 (s), 1435 (m), 1396 (s), 1339 (m), 1261 (m), 1239 (m), 1070 (s), 1051 (s), 1011 (s), 826 (m), 786 (m), 732 (s).

HRMS calculated for $C_{20}H_{18}N_3O^+$ $[M+H]^+$ 316.1444; Found 316.1446.

6-(1-Methyl-1*H*-indol-3-yl)-4-(trifluoromethyl)-2*H*-[1,2'-bipyridin]-2-one (7k)

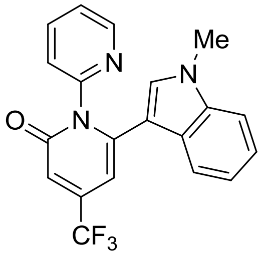

Following General Procedure **D**, starting from **5k** and **6a**, a purification by preparative TLC (DCM/MeOH 19:1) afforded the title compound **7k** (48.7 mg, 0.132 mmol, 66% yield) as a pale yellow solid. **mp** 200-202 °C. **Rf** 0.65 (DCM/MeOH 19:1).

^{1}H NMR (400 MHz, $CDCl_3$) δ 8.60 (d, *J* = 3.7 Hz, 1H, $\underline{H}_{Ar}$), 7.68 (ddd, *J* = 12.2, 6.4, 2.3 Hz, 2H, $\underline{H}_{Ar}$), 7.33-7.13 (m, 5H, $\underline{H}_{Ar}$), 6.88 (s, 1H, C(O)C$\underline{H}$), 6.63 (d, *J* = 1.5 Hz, 1H, $\underline{H}_{Ar}$), 6.51 (s, 1H, $C2_{indole}\underline{H}$), 3.59 (s, 3H, $NC\underline{H}_3$).
^{19}F NMR (376 MHz, $CDCl_3$) δ -66.8.
^{13}C NMR (101 MHz, $CDCl_3$) δ 163.3, 152.5, 149.5, 145.4, 141.8 (q, *J* = 33.5 Hz), 138.7, 136.4, 130.4, 126.3, 124.2, 124.0, 123.1, 122.6 (q, *J* = 274.2 Hz), 121.2, 119.6, 115.7 (q, *J* = 4.0 Hz), 109.8, 108.8, 102.7 (q, *J* = 2.1 Hz), 33.1.
IR ν_{max} 3099 (w), 3062 (w), 2926 (w), 1675 (m), 1610 (m), 1552 (s), 1282 (s), 1170 (s), 1133 (s), 1079 (m), 1006 (m), 932 (m), 857 (s), 734 (s).
HRMS calculated for $C_{20}H_{14}F_3N_3NaO^+$ $[M+Na]^+$ 392.0981; Found 392.0990.

4-(Benzyloxy)-6-(1-methyl-1*H*-indol-3-yl)-2*H*-[1,2'-bipyridin]-2-one (7l)

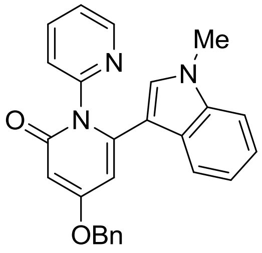

Following General Procedure **D**, starting from **5l** and **6a**, a purification by preparative TLC (DCM/MeOH 19:1) afforded the title compound **7l** (68.4 mg, 0.168 mmol, 84% yield) as an off-white solid. **mp** 162-164 °C. **Rf** 0.37 (DCM/MeOH 19:1).

^{1}H NMR (400 MHz, $CDCl_3$) δ 8.56 (d, *J* = 4.8 Hz, 1H, $\underline{H}_{Ar}$), 7.71 (d, *J* = 8.0 Hz, 1H, $\underline{H}_{Ar}$), 7.62 (td, *J* = 7.7, 1.8 Hz, 1H, $\underline{H}_{Ar}$), 7.48-7.33 (m, 5H, $\underline{H}_{Ar}$), 7.25-7.19 (m, 3H, $\underline{H}_{Ar}$), 7.18-7.12 (m, 2H, $\underline{H}_{Ar}$), 6.47 (s, 1H, $C2_{indole}\underline{H}$), 6.33 (d, *J* = 2.5 Hz, 1H, $\underline{H}_{Ar}$), 6.12 (d, *J* = 2.5 Hz, 1H, $\underline{H}_{Ar}$), 5.11 (s, 2H, $OC\underline{H}_2$), 3.56 (s, 3H, $NC\underline{H}_3$).
^{13}C NMR (101 MHz, $CDCl_3$) δ 167.6, 165.8, 152.9, 149.1, 143.0, 138.3, 136.3, 135.6, 129.9, 128.8 (2C), 128.5, 127.9 (2C), 126.5, 124.6, 123.6, 122.6, 120.6, 119.9, 109.6, 109.1, 102.8, 96.4, 70.3, 33.0.
IR ν_{max} 2982 (m), 2902 (m), 1660 (m), 1637 (m), 1552 (m), 1431 (m), 1398 (m), 1371 (m), 1344 (m), 1244 (m), 1197 (m), 1073 (m), 745 (s).
HRMS calculated for $C_{26}H_{22}N_3O_2^+$ $[M+H]^+$ 408.1707; Found 408.1701.

3-(1-Methyl-1*H*-indol-3-yl)-2-(pyridin-2-yl)isoquinolin-1(2*H*)-one (7n)

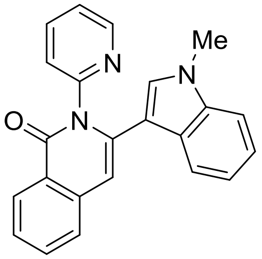

Following General Procedure **D**, starting from **5n** and **6a**, a purification by preparative TLC (DCM/MeOH 19:1) afforded the title compound **7n** (57.6 mg, 0.164 mmol, 82% yield) as a pale yellow solid. **mp** 173-175 °C. **Rf** 0.60 (DCM/MeOH 19:1).

^{1}H NMR (400 MHz, $CDCl_3$) δ 8.52 (ddd, *J* = 4.9, 1.8, 0.7 Hz, 1H, $\underline{H}_{Ar}$), 8.42 (d, *J* = 8.1 Hz, 1H, $\underline{H}_{Ar}$), 7.72 (d, *J* = 8.0 Hz, 1H, $\underline{H}_{Ar}$), 7.65 (ddd, *J* = 8.3, 7.2, 1.3 Hz, 1H, $\underline{H}_{Ar}$), 7.58 (td, *J* = 7.8, 1.9 Hz, 1H, $\underline{H}_{Ar}$), 7.53 (d, *J* = 7.8 Hz, 1H, $\underline{H}_{Ar}$), 7.45 (ddd, *J* = 8.2, 7.2, 1.2 Hz, 1H, $\underline{H}_{Ar}$), 7.22-7.09 (m, 5H, $\underline{H}_{Ar}$), 6.77 (s, 1H, $\underline{H}_{Ar}$), 6.56 (s, 1H, $C2_{indole}\underline{H}$), 3.57 (s, 1H, $NC\underline{H}_3$).
^{13}C NMR (101 MHz, $CDCl_3$) δ 163.9, 153.4, 149.1, 138.1, 137.5, 136.5, 136.3, 132.9, 129.7, 128.3, 127.0, 126.5, 126.0, 125.2, 124.6, 123.4, 122.4, 120.3, 120.0, 110.1, 109.5, 108.1, 32.9.
IR ν_{max} 2976 (w), 2902 (w), 1655 (m), 1618 (m), 1588 (m), 1563 (m), 1471 (m), 1394 (m), 1376 (m), 1334 (m), 1244 (m), 1130 (m), 1064 (m), 788 (m), 752 (m), 742 (s).
HRMS calculated for $C_{23}H_{18}N_3O^+$ $[M+H]^+$ 352.1444; Found 352.1451.

2-(1-Methyl-1*H*-indol-3-yl)-4*H*-[1,2'-bipyridin]-4-one (7o)

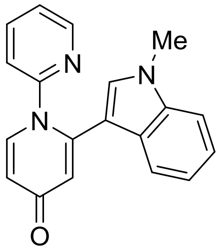

Following General Procedure **D**, starting from **5o** and **6a**, a purification by preparative TLC (DCM/MeOH 19:1) afforded the title compound **7o** (34.4 mg, 0.114 mmol, 57% yield) as an off-white solid. **mp** 150-152 °C. **Rf** 0.34 (DCM/MeOH 19:1).

^{1}H NMR (400 MHz, $CDCl_3$) δ 8.52 (d, *J* = 3.9 Hz, 1H), 7.90 (d, *J* = 7.8 Hz, 1H), 7.43-7.33 (m, 2H), 7.24 (d, *J* = 8.6 Hz, 1H), 7.20-7.13 (m, 2H), 7.02 (t, *J* = 7.4 Hz, 1H), 6.81 (d, *J* = 8.1 Hz, 1H), 6.77 (s, 1H), 6.64 (d, *J* = 2.5 Hz, 1H), 6.50 (dd, *J* = 7.8, 2.5 Hz, 1H), 3.68 (s, 3H).

^{13}C NMR (101 MHz, $CDCl_3$) δ 179.9, 154.0, 149.2, 144.5, 140.8, 137.8, 136.6, 129.6, 125.8, 123.2, 122.7, 121.5, 120.9, 119.9, 119.6, 117.6, 109.7, 109.3, 33.2.

IR ν_{max} 3056 (w), 2933 (w), 1625 (s), 1566 (s), 1547 (s), 1466 (s), 1447 (s), 1430 (s), 1277 (s), 1246 (s), 790 (m), 740 (s).

HRMS calculated for $C_{19}H_{16}N_3O^+$ $[M+H]^+$ 302.1288; Found 302.1284.

6-(5-Bromo-1-methyl-1*H*-indol-3-yl)-2*H*-[1,2'-bipyridin]-2-one (7q)

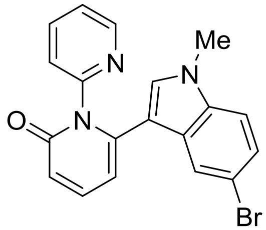

Following General Procedure **D**, starting from **5a** and **6b**, a purification by preparative TLC (DCM/MeOH 19:1) afforded the title compound **7q** (58.0 mg, 0.156 mmol, 76% yield) as a white solid. **mp** 215-217 °C. **Rf** 0.39 (DCM/MeOH 19:1).

^{1}H NMR (400 MHz, $CDCl_3$) δ 8.54 (d, *J* = 3.7 Hz, 1H, $\underline{H}_{Ar}$), 7.79 (d, *J* = 1.7 Hz, 1H, $\underline{H}_{Ar}$), 7.64 (d, *J* = 1.9 Hz, 1H, $\underline{H}_{Ar}$), 7.49 (dd, *J* = 9.2, 6.9 Hz, 1H, $\underline{H}_{Ar}$), 7.28 (dd, *J* = 8.7, 1.8 Hz, 1H, $\underline{H}_{Ar}$), 7.22 (ddd, *J* = 7.5, 4.9, 0.9 Hz, 1H, $\underline{H}_{Ar}$), 7.14 (d, *J* = 7.9 Hz, 1H, $\underline{H}_{Ar}$), 7.07 (d, *J* = 8.7 Hz, 1H, $\underline{H}_{Ar}$), 6.62 (dd, *J* = 9.2, 1.1 Hz, 1H, $\underline{H}_{Ar}$), 6.50 (s, 1H, $C2_{indole}\underline{H}$), 6.42 (dd, *J* = 6.9, 1.2 Hz, 1H, $\underline{H}_{Ar}$), 3.55 (s, 3H, $NC\underline{H}_3$).

^{13}C NMR (101 MHz, $CDCl_3$) δ 164.0, 152.9, 149.2, 142.4, 140.2, 138.4, 134.9, 130.8, 128.1, 125.5, 124.1, 123.8, 122.3, 119.2, 114.1, 111.1, 109.2, 108.0, 33.2.

IR ν_{max} 3093 (w), 2920 (w), 1661 (s), 1588 (s), 1548 (s), 1467 (s), 1434 (m), 1142 (m), 799 (s), 785 (s), 746 (m), 730 (m).

HRMS calculated for $C_{19}H_{14}{}^{79}BrN_3NaO^+$ $[M+Na]^+$ 402.0212; found 402.0210.

6-(1*H*-Indol-3-yl)-2*H*-[1,2'-bipyridin]-2-one (7r)

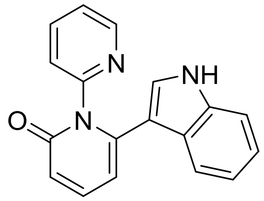

Following General Procedure **D**, starting from **5a** and **6c**, a purification by preparative TLC (DCM/MeOH 19:1) afforded the title compound **7r** (48.2 mg, 0.168 mmol, 84% yield) as a pale brown solid. **mp** 175-177 °C. **Rf** 0.35 (DCM/MeOH 19:1).

^{1}H NMR (400 MHz, MeOD) δ 8.47 (ddd, *J* = 4.9, 1.7, 0.6 Hz, 1H, $\underline{H}_{Ar}$), 7.78 (td, *J* = 7.8, 1.9 Hz, 1H, $\underline{H}_{Ar}$), 7.73 (dd, *J* = 9.1, 7.1 Hz, 1H, $\underline{H}_{Ar}$), 7.62 (d, *J* = 7.6 Hz, 1H, $\underline{H}_{Ar}$), 7.36 (ddd, *J* = 7.5, 5.0, 0.9 Hz, 1H, $\underline{H}_{Ar}$), 7.30-7.27 (m, 2H, $\underline{H}_{Ar}$), 7.14-7.05 (m, 2H, $\underline{H}_{Ar}$), 6.72-6.67 (m, 2H, $\underline{H}_{Ar}$), 6.62 (dd, *J* = 9.1, 1.0 Hz, 1H, $\underline{H}_{Ar}$). N$\underline{H}$ is not resolved in MeOD but could be observed at 10.45 ppm as a broad singlet in acetone-d6.

^{13}C NMR (101 MHz, MeOD) δ 166.2, 153.7, 149.9, 145.8, 143.1, 140.3, 137.2, 127.3, 127.2, 125.6, 125.5, 123.5, 121.4, 120.0, 118.2, 112.6, 110.6, 110.5.

IR ν_{max} 3124 (br), 1641 (s), 1545 (s), 1510 (m), 1461 (m), 1431 (s), 1389 (s), 1144 (m), 1016 (m), 801 (s), 794 (s), 739 (s).

HRMS calculated for $C_{18}H_{14}N_3O^+$ $[M+H]^+$ 288.1131; found 288.1132.

6-(6-Bromo-1*H*-indol-3-yl)-2*H*-[1,2'-bipyridin]-2-one (7s)

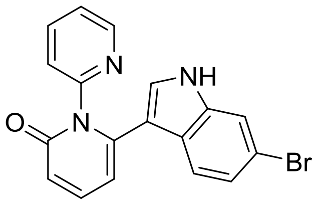

Following General Procedure **D**, starting from **5a** and **6d**, a purification by preparative TLC (DCM/MeOH 24:1) afforded the title compound **7s** (56.2 mg, 0.153 mmol, 77% yield) as a pale brown solid. **mp** 213-215 °C. **Rf** 0.28 (DCM/MeOH 19:1).

^{1}H NMR (400 MHz, $CDCl_3$) δ 9.66 (bs, 1H, N<u>H</u>), 8.44 (dd, *J* = 4.8, 1.1 Hz, 1H, $\underline{H}_{Ar}$), 7.61-7.50 (m, 2H, $\underline{H}_{Ar}$), 7.47 (d, *J* = 8.6 Hz, 1H, $\underline{H}_{Ar}$), 7.31 (d, *J* = 1.5 Hz, 1H, $\underline{H}_{Ar}$), 7.18 (dd, *J* = 8.6, 1.7 Hz, 1H, $\underline{H}_{Ar}$), 7.16-7.08 (m, 1H, $\underline{H}_{Ar}$), 7.06 (d, *J* = 7.9 Hz, 1H, $\underline{H}_{Ar}$), 6.60 (dd, *J* = 9.2, 1.0 Hz, 1H, $\underline{H}_{Ar}$), 6.53 (d, *J* = 2.7 Hz, 1H, $\underline{H}_{Ar}$), 6.48 (dd, *J* = 7.0, 1.0 Hz, 1H, $\underline{H}_{Ar}$).

^{13}C NMR (101 MHz, $CDCl_3$) δ 164.4, 152.7, 149.2, 143.2, 140.8, 138.6, 136.4, 126.5, 124.9, 124.0, 123.9, 123.8, 120.5, 118.7, 116.1, 114.8, 110.2, 108.7.

IR ν_{max} 3253 (br), 1665 (s), 1585 (m), 1546 (s), 1467 (m), 1441 (m), 1104 (m), 803 (s), 788 (m).

HRMS calculated for $C_{18}H_{12}{}^{79}BrN_3NaO^+$ $[M+Na]^+$ 388.0056; found 388.0054.

4. Same general procedure D for the synthesis of 8-(indol-3-yl)-quinoline *N*-oxides

8-(1-Methyl-1*H*-indol-3-yl)quinoline 1-oxide (12a)

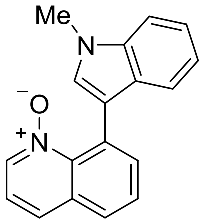

Following General Procedure **D**, starting from quinoline *N*-oxide **11a** and **6a**, a purification by preparative TLC (DCM/MeOH 19:1) afforded the title compound **12a** (32.8 mg, 0.120 mmol, 60% yield) as a yellow oil. **Rf** 0.80 (DCM/MeOH 19:1).

^{1}H NMR (400 MHz, $CDCl_3$) δ 8.37 (dd, *J* = 6.0, 1.2 Hz, 1H, $\underline{H}_{Ar}$), 7.83 (dd, *J* = 8.1, 1.6 Hz, 1H, $\underline{H}_{Ar}$), 7.73 (dd, *J* = 8.5, 1.1 Hz, 1H, $\underline{H}_{Ar}$), 7.67 (dd, *J* = 7.2, 1.6 Hz, 1H, $\underline{H}_{Ar}$), 7.59 (dd, *J* = 8.0, 7.3 Hz, 1H, $\underline{H}_{Ar}$), 7.41-7.32 (m, 2H, $\underline{H}_{Ar}$), 7.29-7.17 (m, 2H, $\underline{H}_{Ar}$), 7.12 (s, 1H, $C2_{indole}\underline{H}$), 7.09 (ddd, *J* = 8.0, 7.0, 1.1 Hz, 1H, $\underline{H}_{Ar}$), 3.86 (s, 3H, $NC\underline{H}_3$).

^{13}C NMR (101 MHz, $CDCl_3$) δ 140.9, 137.2, 136.3, 135.5, 132.6, 129.3, 129.2, 127.9, 127.9, 127.3, 125.6, 121.5, 121.0, 120.1, 119.6, 117.0, 109.3, 33.0.

IR ν_{max} 3051 (w), 2930 (w), 1707 (m), 1613 (w), 1570 (m), 1476 (m), 1418 (m), 1366 (m), 1302 (m), 1245 (s), 1160 (w), 1058 (w), 1021 (w), 902 (w), 824 (m).

HRMS calculated for $C_{18}H_{15}N_2O^+$ $[M+H]^+$ 275.1179; found 275.1188.

2-Methyl-8-(1-methyl-1*H*-indol-3-yl)quinoline 1-oxide (12b)

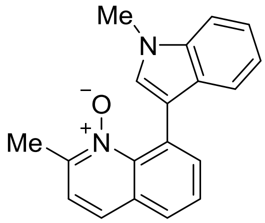

Following General Procedure **D**, starting from 2-methylquinoline *N*-oxide **11b** and **6a**, a purification by preparative TLC (DCM/MeOH 19:1) afforded the title compound **12b** (38.2 mg, 0.132 mmol, 66% yield) as a yellow oil. **Rf** 0.85 (DCM/MeOH 19:1).

^{1}H NMR (400 MHz, $CDCl_3$) δ 7.80 (dd, *J* = 8.0, 1.6 Hz, 1H, $\underline{H}_{Ar}$), 7.66-7.62 (m, 2H, $\underline{H}_{Ar}$), 7.54 (dd, *J* = 8.0, 7.3 Hz, 1H, $\underline{H}_{Ar}$), 7.34 (m, 1H, $\underline{H}_{Ar}$), 7.30 (m, 2H, $\underline{H}_{Ar}$), 7.22 (ddd, *J* = 8.2, 7.0, 1.2 Hz, 1H, $\underline{H}_{Ar}$), 7.14 (s, 1H, $C2_{indole}\underline{H}$), 7.05 (ddd, *J* = 7.9, 7.0, 1.0 Hz, 1H, $\underline{H}_{Ar}$), 3.86 (s, 3H, $NC\underline{H}_3$), 2.56 (s, 3H, $C\underline{H}_3$).

^{13}C NMR (101 MHz, $CDCl_3$) δ 146.8, 140.9, 136.3, 135.6, 131.3, 129.4, 128.8, 127.9, 126.9, 126.9, 124.5, 123.0, 121.5, 120.1, 119.3, 117.8, 109.3, 33.0, 19.4.

IR ν_{max} 3048 (w), 2936 (w), 2837 (w), 1665 (s), 1548 (s), 1466 (s), 1436 (m), 1362 (m), 1339 (m), 1228 (m), 1159 (m), 1125 (m), 1033 (m), 846 (m), 804 (s), 737 (m).

HRMS calculated for $C_{19}H_{17}N_2O^+$ $[M+H]^+$ 289.1335; found 289.1346.

6-Methoxy-8-(1-methyl-1*H*-indol-3-yl)quinoline 1-oxide (12c)

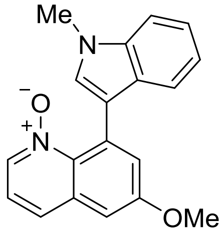

Following General Procedure **D**, starting from 6-methoxyquinoline *N*-oxide **11c** and **6a**, a purification by preparative TLC (DCM/MeOH 19:1) afforded the title compound **12c** (34.9 mg, 0.115 mmol, 57% yield) as a yellow oil. **Rf** 0.78 (DCM/MeOH 19:1).

^{1}H NMR (400 MHz, $CDCl_3$) δ 8.22 (dd, *J* = 6.0, 1.2 Hz, 1H, $\underline{H}_{Ar}$), 7.61 (dd, *J* = 8.4, 1.2 Hz, 1H, $\underline{H}_{Ar}$), 7.39 (dt, *J* = 7.9, 1.0 Hz, 1H, $\underline{H}_{Ar}$), 7.34 (dt, *J* = 8.4, 0.9 Hz, 1H, $\underline{H}_{Ar}$), 7.31 (d, *J* = 2.9 Hz, 1H, $\underline{H}_{Ar}$), 7.25-7.17 (m, 2H, $\underline{H}_{Ar}$), 7.12 (s, 1H, $C2_{indole}\underline{H}$), 7.12-7.05 (m, 2H, $\underline{H}_{Ar}$), 3.94 (s, 3H, $OC\underline{H}_3$), 3.85 (s, 3H, $NC\underline{H}_3$).

^{13}C NMR (101 MHz, $CDCl_3$) δ 158.0, 136.8, 136.3, 135.4, 134.1, 131.1, 129.2, 127.4, 126.8, 124.7, 121.6, 121.5, 120.1, 119.7, 116.7, 109.3, 106.0, 55.7, 33.0.

IR ν_{max} 3051 (w), 2985 (w), 1665 (m), 1609 (w), 1584 (m), 1552 (w), 1465 (w), 1436 (w), 1362 (w), 1266 (s), 1230 (w), 1159 (w), 1126 (w), 1030 (w), 805 (w).

HRMS calculated for $C_{19}H_{17}N_2O_2^+$ $[M+H]^+$ 305.1285; found 305.1287.

8-(1-methyl-1*H*-indol-3-yl)-6-phenylquinoline 1-oxide (12d)

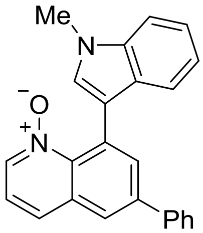

Following General Procedure **D**, starting from 6-phenylquinoline *N*-oxide **11d** and **6a**, a purification by preparative TLC (DCM/MeOH 19:1) afforded the title compound **12d** (51.3 mg, 0.146 mmol, 73% yield) as a yellow oil. **Rf** 0.58 (DCM/MeOH 19:1).
^{1}H NMR (400 MHz, $CDCl_3$) δ 8.34 (dd, *J* = 6.0, 1.2 Hz, 1H, $\underline{H}_{Ar}$), 7.98 (d, *J* = 2.1 Hz, 1H, $\underline{H}_{Ar}$), 7.93 (d, *J* = 2.1 Hz, 1H, $\underline{H}_{Ar}$), 7.75 (dd, *J* = 8.4, 1.1 Hz, 1H, $\underline{H}_{Ar}$), 7.73-7.65 (m, 2H, $\underline{H}_{Ar}$), 7.46 (t, *J* = 7.4 Hz, 2H, $\underline{H}_{Ar}$), 7.41 (dd, *J* = 8.5, 7.3 Hz, 2H, $\underline{H}_{Ar}$), 7.34 (d, *J* = 8.2 Hz, 1H, $\underline{H}_{Ar}$), 7.25-7.19 (m, 2H, $\underline{H}_{Ar}$), 7.16 (s, 1H, $C2_{indole}\underline{H}$), 7.12-7.05 (m, 1H, $\underline{H}_{Ar}$), 3.84 (s, 3H, $NC\underline{H}_3$).
^{13}C NMR (101 MHz, $CDCl_3$) δ 140.2, 139.9, 138.9, 136.9, 136.2, 134.8, 132.8, 129.6, 129.1, 129.0, 128.2, 127.4, 127.3, 125.8, 125.1, 121.4, 121.2, 120.0, 119.5, 116.7, 109.2, 32.8.
IR ν_{max} 3050 (w), 2975 (m), 2932 (m), 2873 (m), 1576 (m), 1478 (m), 1363 (m), 1332 (m), 1288 (m), 1250 (m), 1231 (m), 1170 (m), 1133 (m), 1116 (m), 1060 (m), 846 (m), 797 (m), 762 (s), 736 (s).
HRMS calculated for $C_{24}H_{19}N_2O^+$ $[M+H]^+$ 351.1492; found 351.1490.

5. Product modifications

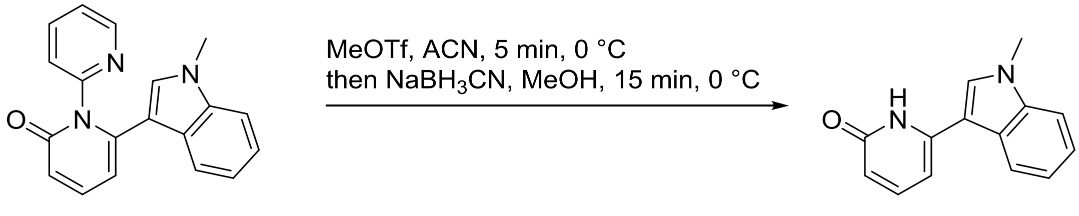

7a **13**

Following a modified reported procedure,[10] **7a** (90 mg, 0.30 mmol, 1.0 equiv) was solubilized in dry ACN (0.6 mL) under N_2. MeOTf (0.10 mL, 0.90 mmol, 3.0 equiv) was added to the mixture at 0 °C. The mixture was stirred 5 min at 0 °C and MeOH (3 mL) was added. $NaBH_3CN$ (94 mg, 1.5 mmol, 5.0 equiv) was added portionwise to the solution at 0 °C. The mixture was stirred 15 min at 0 °C and then quenched with H_2O (2 mL). The mixture was diluted with EtOAc (5 mL) and the layers were separated. The aqueous layer was extracted three times with EtOAc (5 mL). The organic layers were combined, dried over magnesium sulfate dehydrate, filtered and concentrated under reduced pressure. The crude residue was purified by preparative TLC using DCM/MeOH 19:1 as eluent to afforded **13** (50 mg, 0.22, 74%) as an off-white solid. **mp** 217-219 °C. **Rf** 0.53 (DCM/MeOH 19:1).

^{1}H NMR (400 MHz, $CDCl_3$) δ 12.08 (s, 1H, NH), 7.96 (s, 1H, H_{Ar}), 7.89 (d, *J* = 8.0 Hz, 1H, H_{Ar}), 7.49 (dd, *J* = 8.9, 7.2 Hz, 1H, H_{Ar}), 7.38 (d, *J* = 8.1 Hz, 1H, H_{Ar}), 7.31 (t, *J* = 7.4 Hz, 1H, H_{Ar}), 7.23 (t, *J* = 7.3 Hz, 1H, H_{Ar}), 6.66 (d, *J* = 7.0 Hz, 1H, H_{Ar}), 6.39 (d, *J* = 8.9 Hz, 1H, H_{Ar}), 3.87 (s, 3H, NCH_3).

^{13}C NMR (101 MHz, $CDCl_3$) δ 165.5, 143.0, 142.1, 137.8, 130.0, 125.3, 122.8, 121.2, 119.9, 115.9, 110.2, 109.1, 104.1, 33.5.

IR ν_{max} 3104 (m), 3043 (w), 2944 (br), 1650 (s), 1601 (s), 1570 (m), 1532 (m), 1460 (s), 1367 (s), 1256 (m), 1238 (m), 1137 (m), 1101 (s), 980 (m), 894 (s), 777 (s), 736 (s), 713 (s).

HRMS calculated for $C_{14}H_{13}N_2O^+$ $[M+H]^+$ 225.1022; found 225.1027.

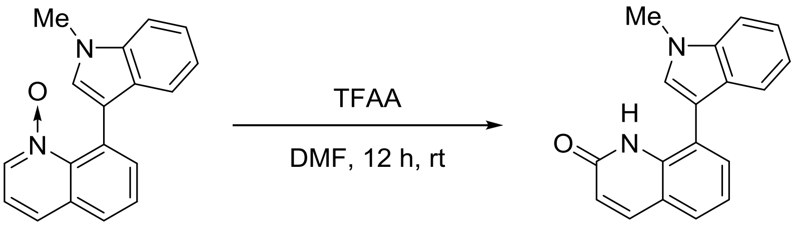

12a **14**

Following a reported procedure,[11] **12a** (27.4 mg, 0.100 mmol, 1.00 equiv) was solubilized in dry DMF (3 mL). TFAA (140 µL, 1.00 mmol, 10.0 equiv) was added to the mixture at rt. The mixture was stirred overnight and the TFAA excess was eliminated under reduced pressure. The mixture was poured into H_2O (30 mL) and extracted three times with EtOAc (10 mL). The organic layers were combined, washed twice with brine (5 mL), dried over magnesium sulfate dehydrate, filtered and concentrated under reduced pressure. The crude residue was purified by preparative TLC using DCM/MeOH 24:1 as eluent to afforded **14** (17.0 mg, 0.062 mmol, 62%) as a pale brown solid. **mp** 192-194 °C. **Rf** 0.92 (DCM/MeOH 19:1).

^{1}H NMR (400 MHz, $CDCl_3$) δ 9.03 (s, 1H, NH), 7.83 (d, *J* = 9.5 Hz, 1H, H_{Ar}), 7.61-7.54 (m, 2H, H_{Ar}), 7.45 (t, *J* = 7.4 Hz, 2H, H_{Ar}), 7.36-7.31 (m, 1H, H_{Ar}), 7.30 (d, *J* = 7.7 Hz, 1H, H_{Ar}), 7.23 (s, 1H, $C2_{indole}H$), 7.20-7.15 (m, 1H, H_{Ar}), 6.66 (d, *J* = 9.5 Hz, 1H, H_{Ar}), 3.91 (s, 3H, NCH_3). **^{13}C NMR** (101 MHz, $CDCl_3$) δ 162.6, 141.2, 137.4, 136.4, 132.4, 127.9, 127.0, 126.8, 123.0, 122.5, 122.0, 121.8, 120.7, 120.1, 119.5, 110.0, 109.5, 33.3. Spectra data matched with the values reported in literature.[7]

---

[10] Smout, V.; Peschiulli, A.; Verbeeck, S.; Mitchell, E. A.; Herrebout, W.; Bultinck, P.; Vande Velde, C. M. L.; Berthelot, D.; Meerpoel, L.; Maes, B. U. W. *J. Org. Chem.* **2013**, *78*, 9803–9814.

[11] Konno, K.; Hashimoto, K.; Shirahama, H.; Matsumoto, T. Heterocycles 1986, 24, 2169–2172.

6. Spectra of new compounds

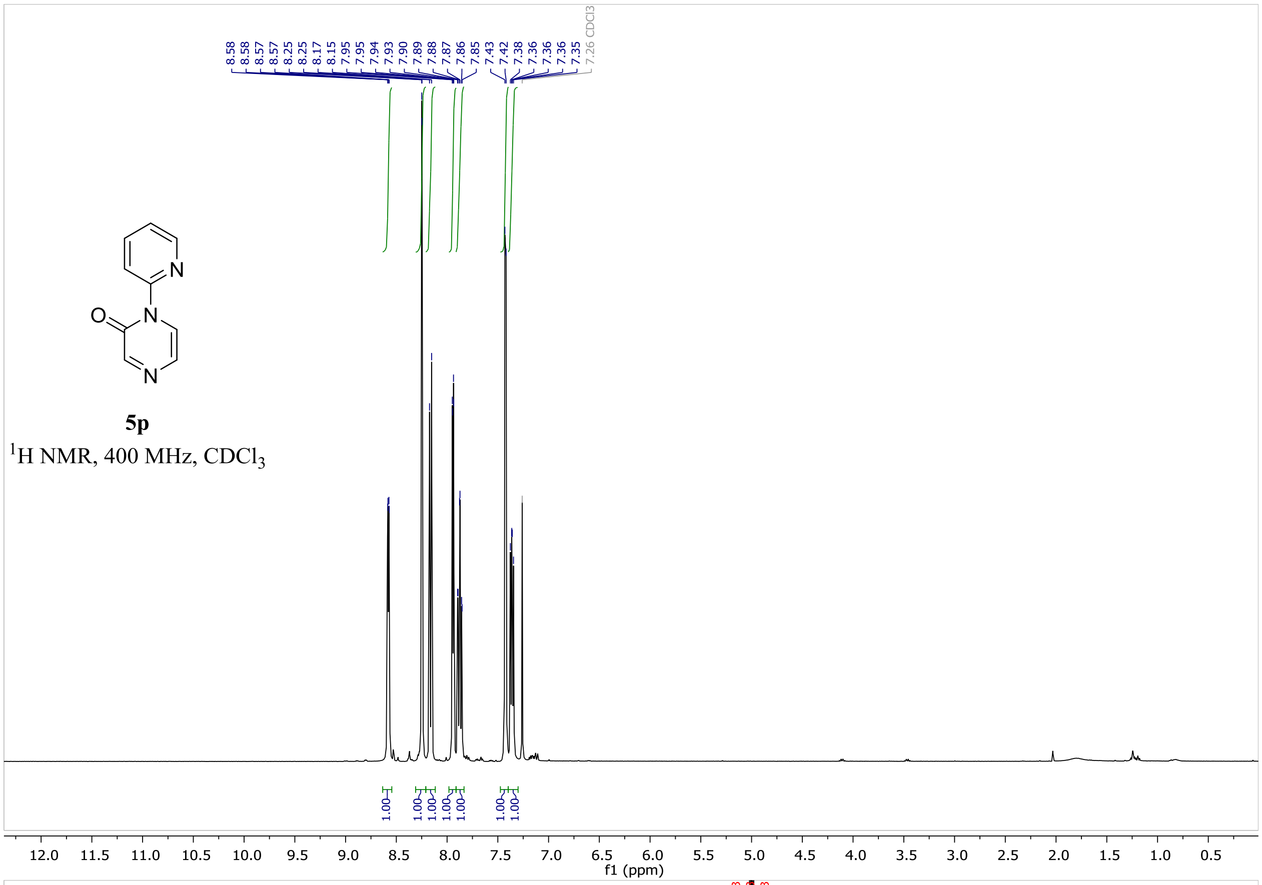

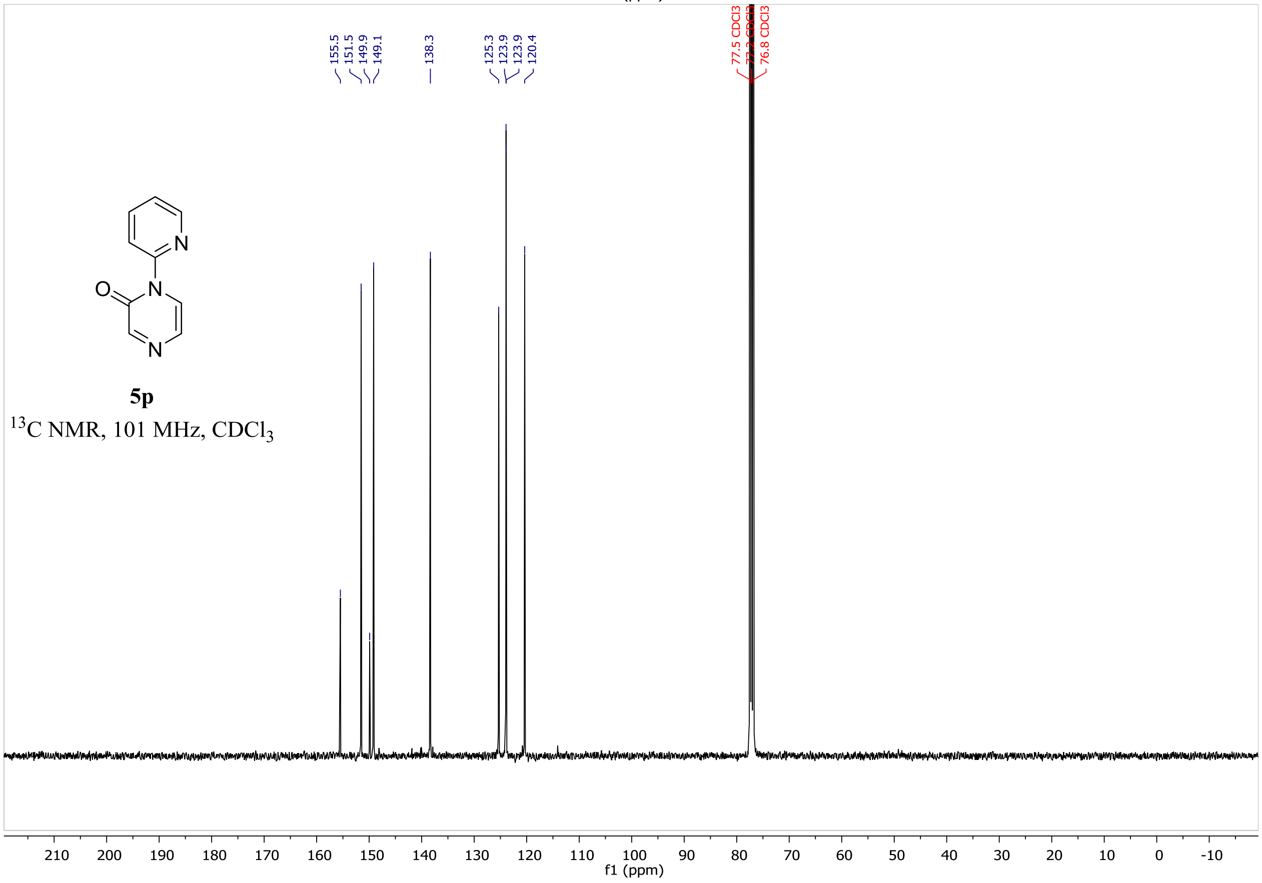

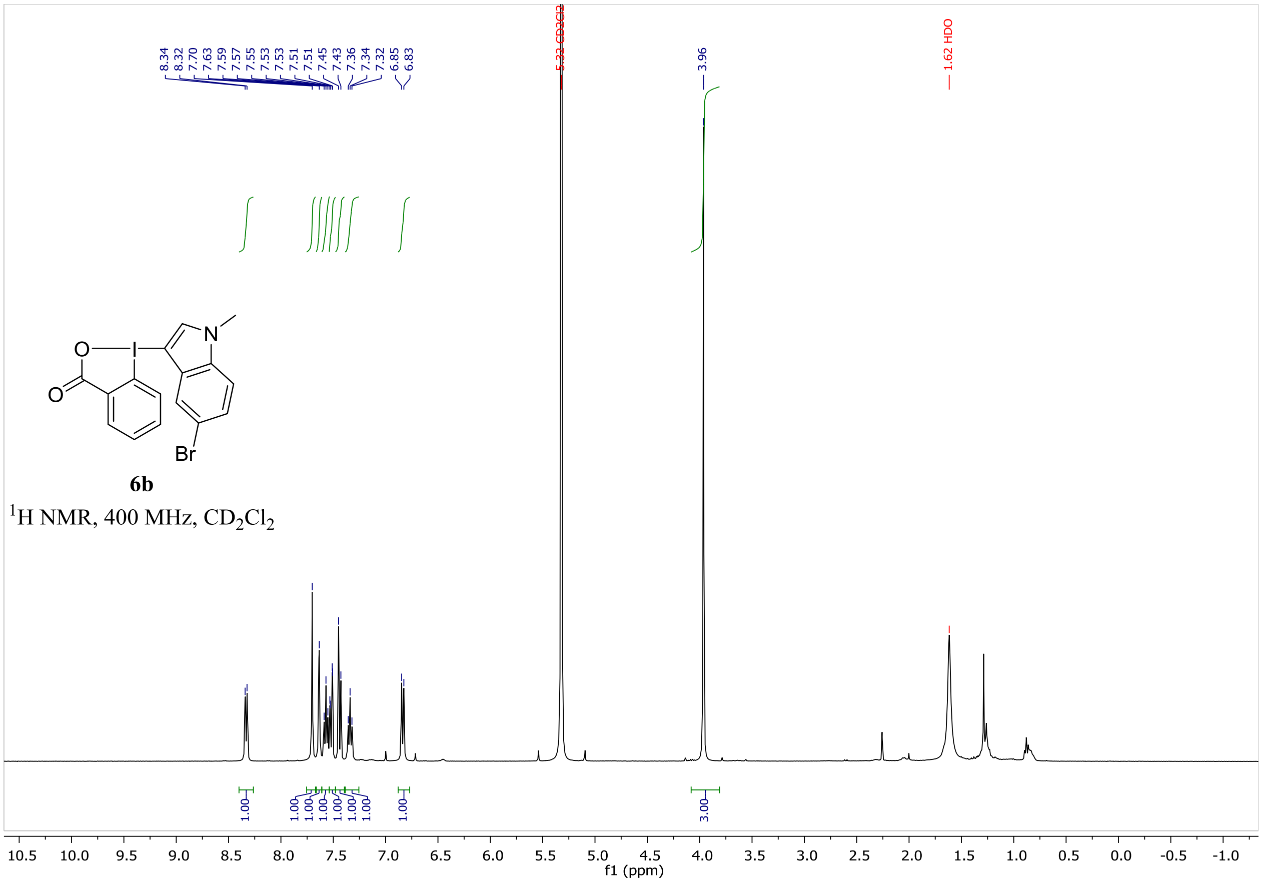

6b

^{1}H NMR, 400 MHz, CD_2Cl_2

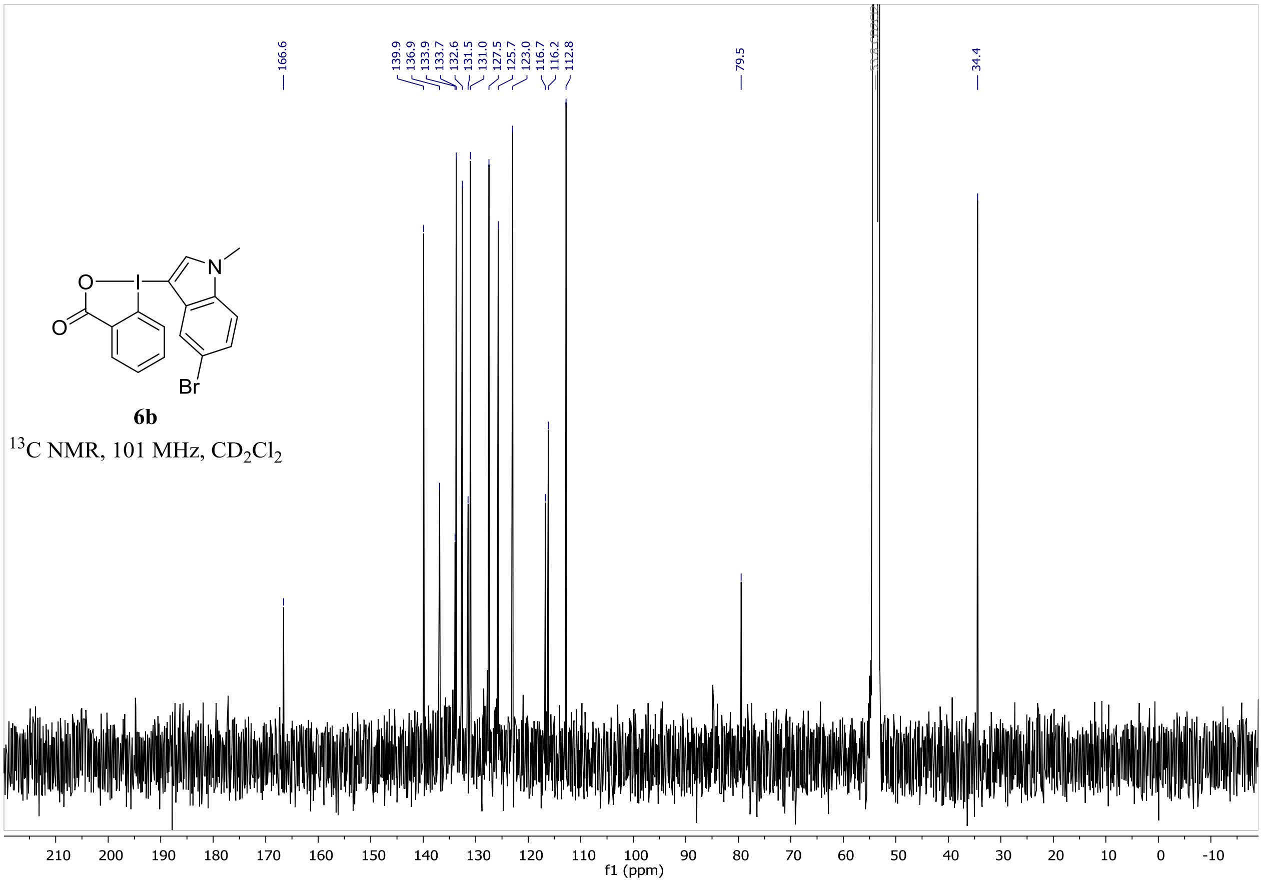

6b

^{13}C NMR, 101 MHz, CD_2Cl_2

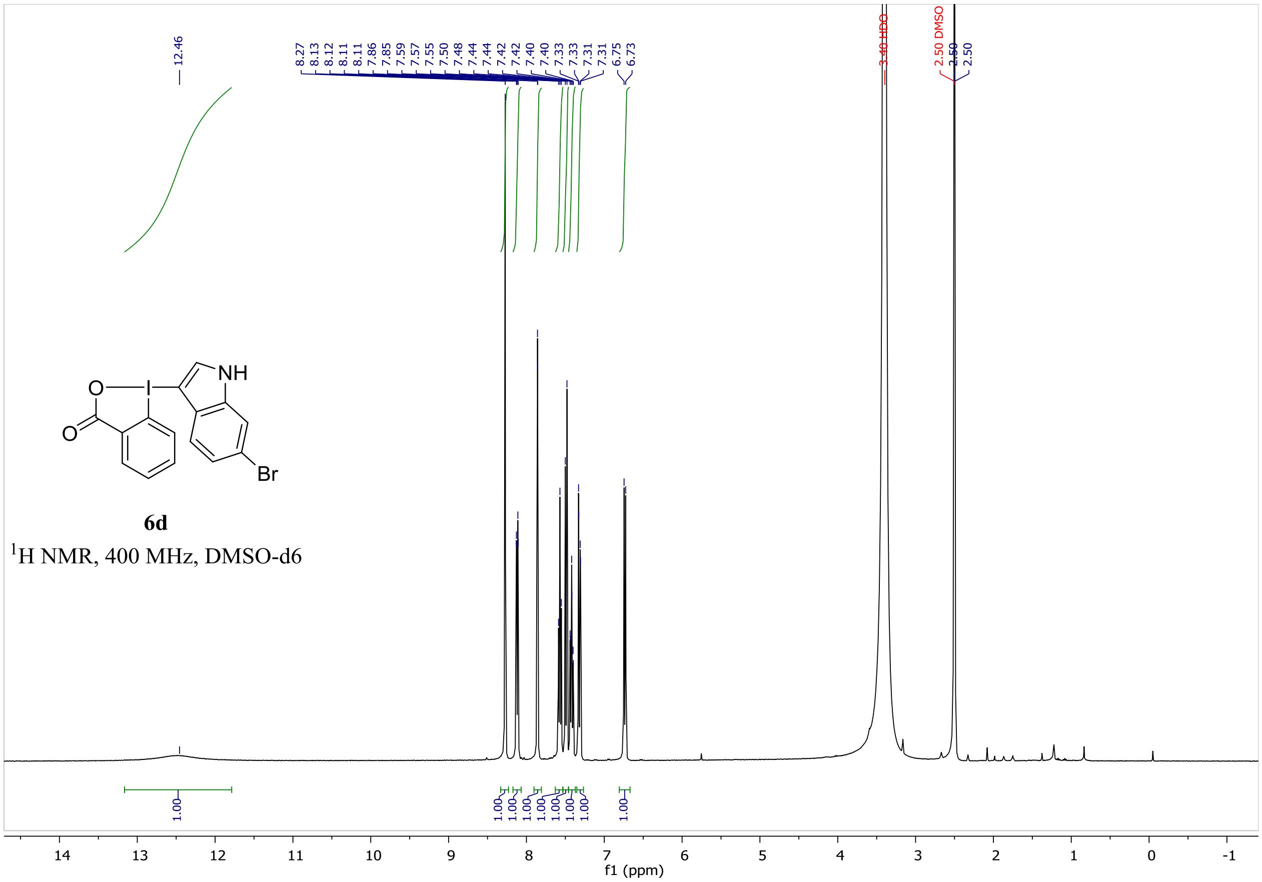

6d

^{1}H NMR, 400 MHz, DMSO-d6

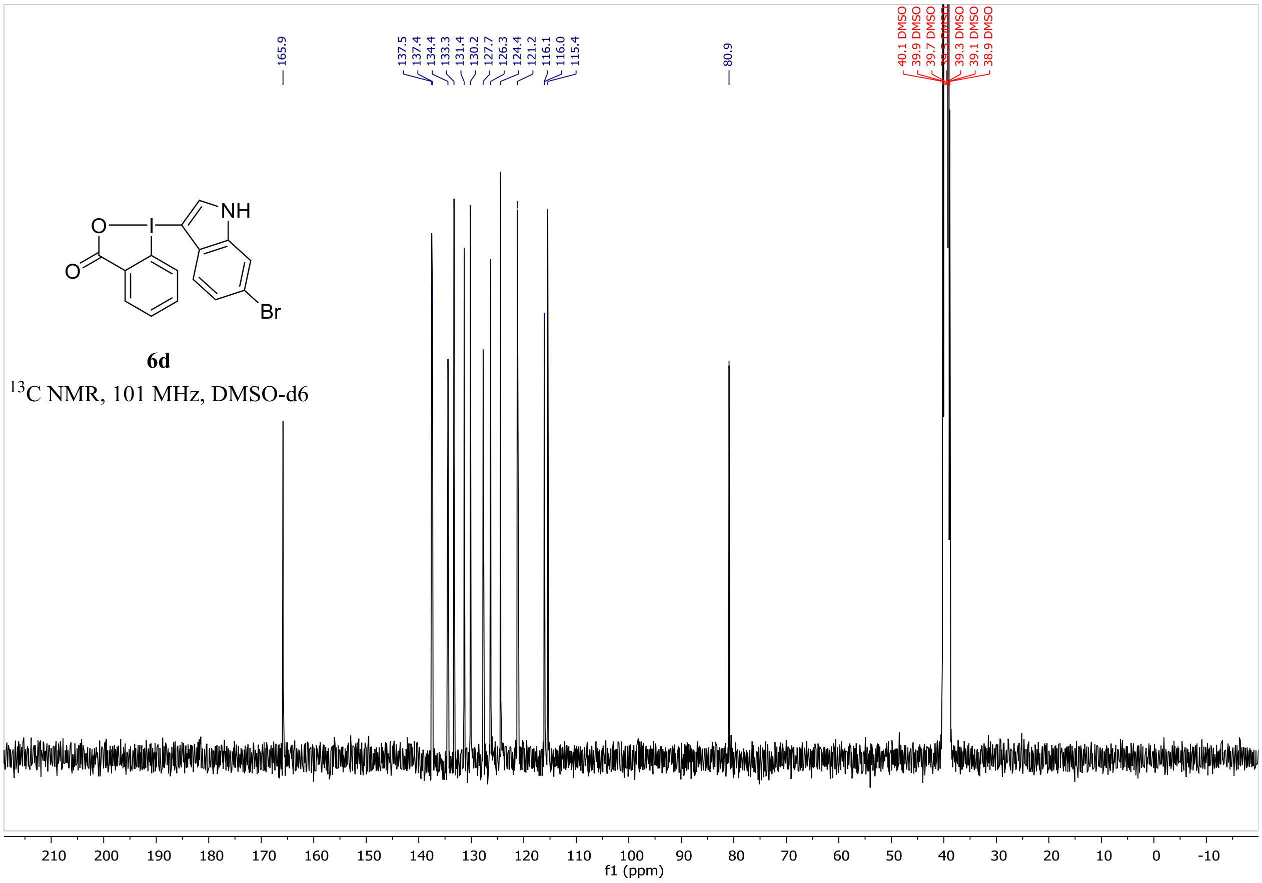

6d

^{13}C NMR, 101 MHz, DMSO-d6

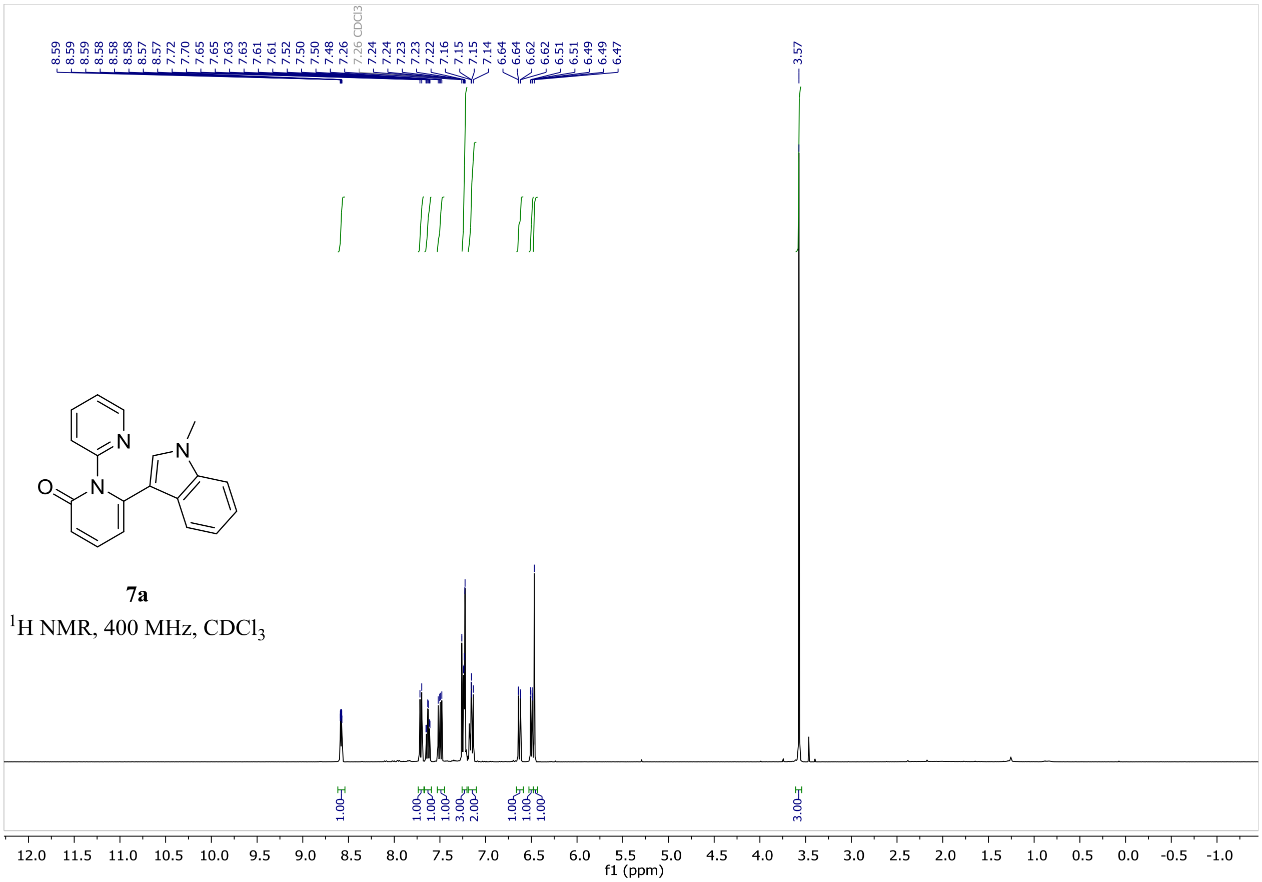
8.59 8.59 8.59 8.58 8.58 8.58 8.57 7.72 7.70 7.65 7.65 7.63 7.63 7.61 7.61 7.52 7.50 7.50 7.48 7.26 7.26 CDCl3 7.24 7.24 7.23 7.23 7.22 7.16 7.15 7.15 7.14 6.64 6.64 6.62 6.62 6.51 6.51 6.49 6.49 6.47
3.57
N
O
N
N
7a
1H NMR, 400 MHz, CDCl3
1.00 1.00 1.00 1.00 3.00 2.00 1.00 1.00 1.00 3.00
12.0 11.5 11.0 10.5 10.0 9.5 9.0 8.5 8.0 7.5 7.0 6.5 6.0 5.5 5.0 4.5 4.0 3.5 3.0 2.5 2.0 1.5 1.0 0.5 0.0 -0.5 -1.0
f1 (ppm)

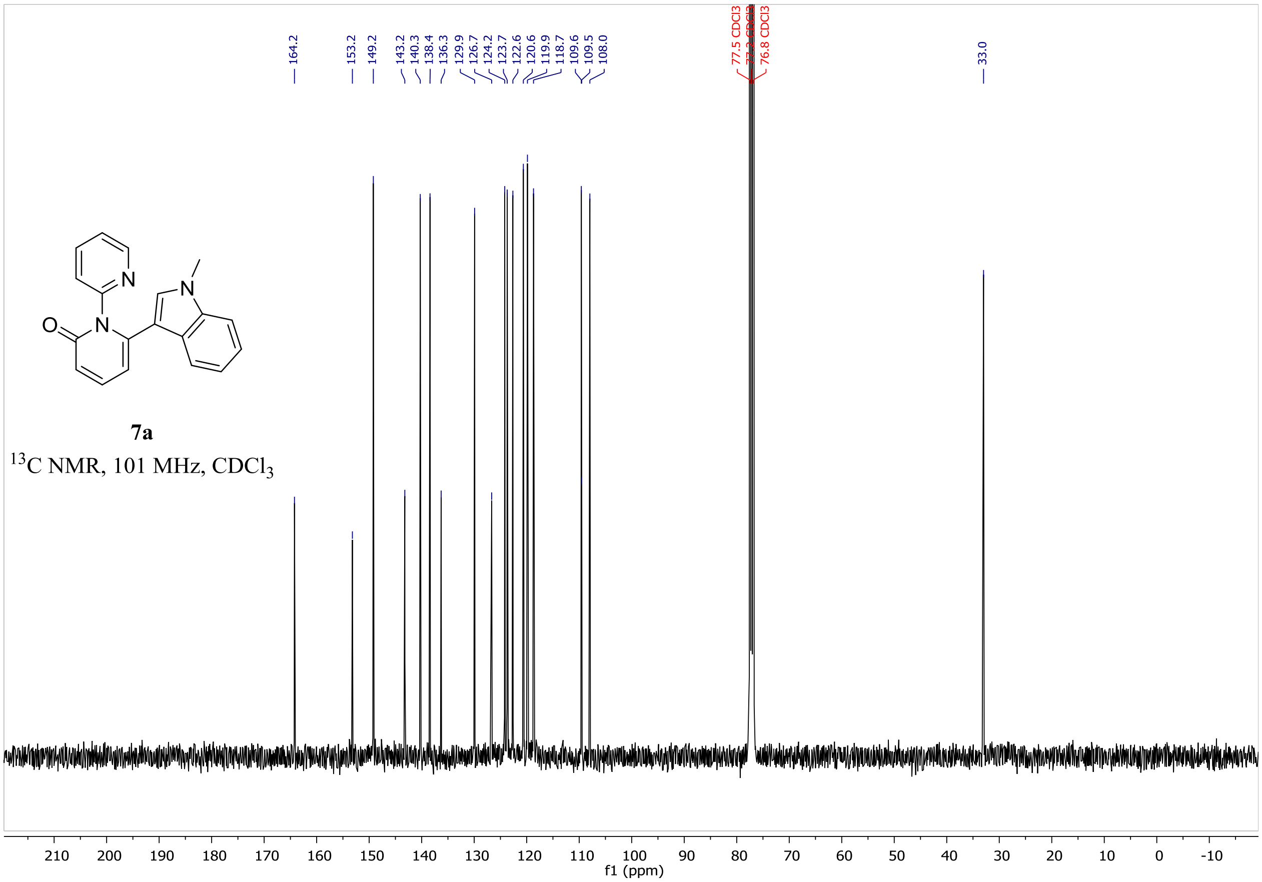
164.2
153.2 149.2
143.2 140.3 138.4 136.3 129.9 126.7 124.2 123.7 122.6 120.6 119.9 118.7 109.6 109.5 108.0
77.5 CDCl3
76.8 CDCl3
33.0
N
O
N
N
7a
13C NMR, 101 MHz, CDCl3
210 200 190 180 170 160 150 140 130 120 110 100 90 80 70 60 50 40 30 20 10 0 -10
f1 (ppm)

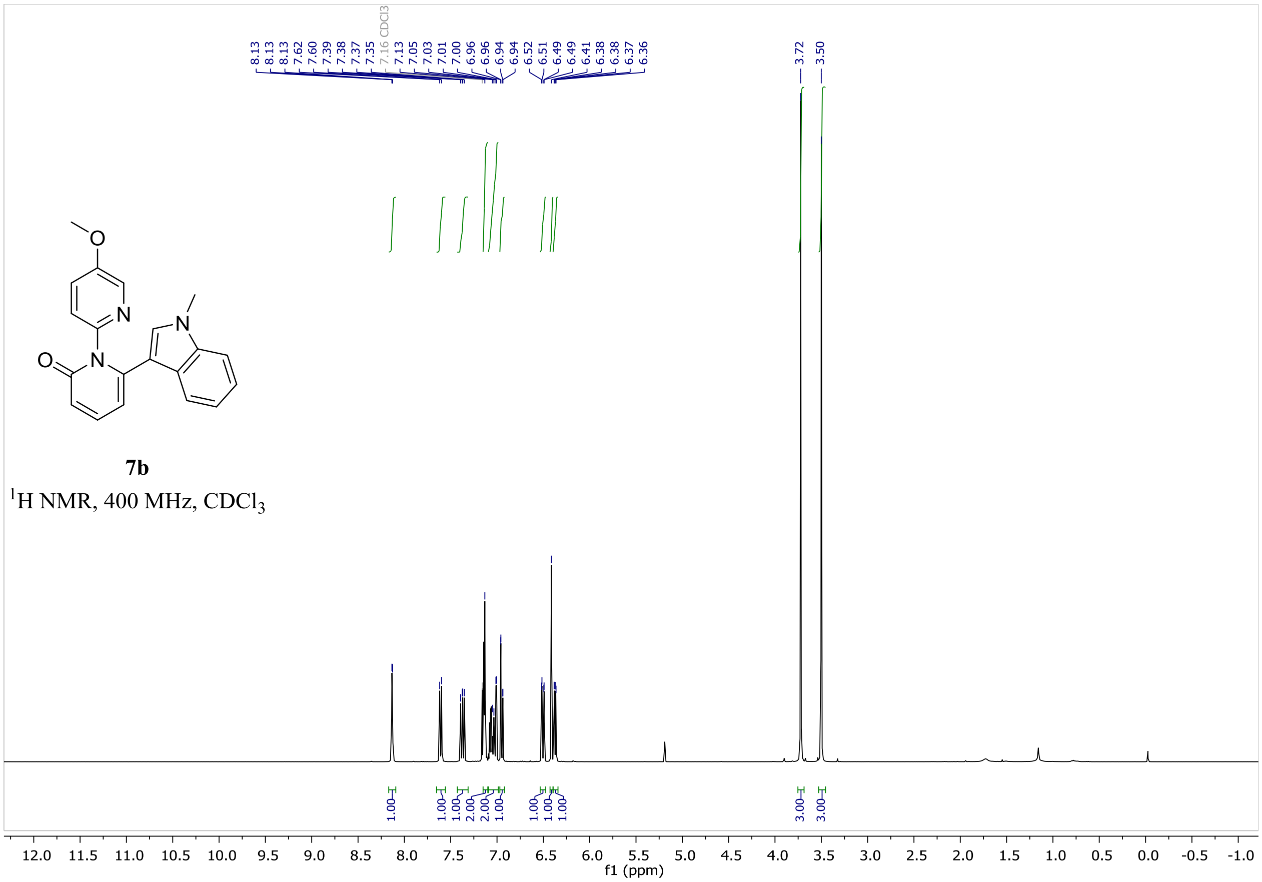

7b
^{1}H NMR, 400 MHz, $CDCl_3$

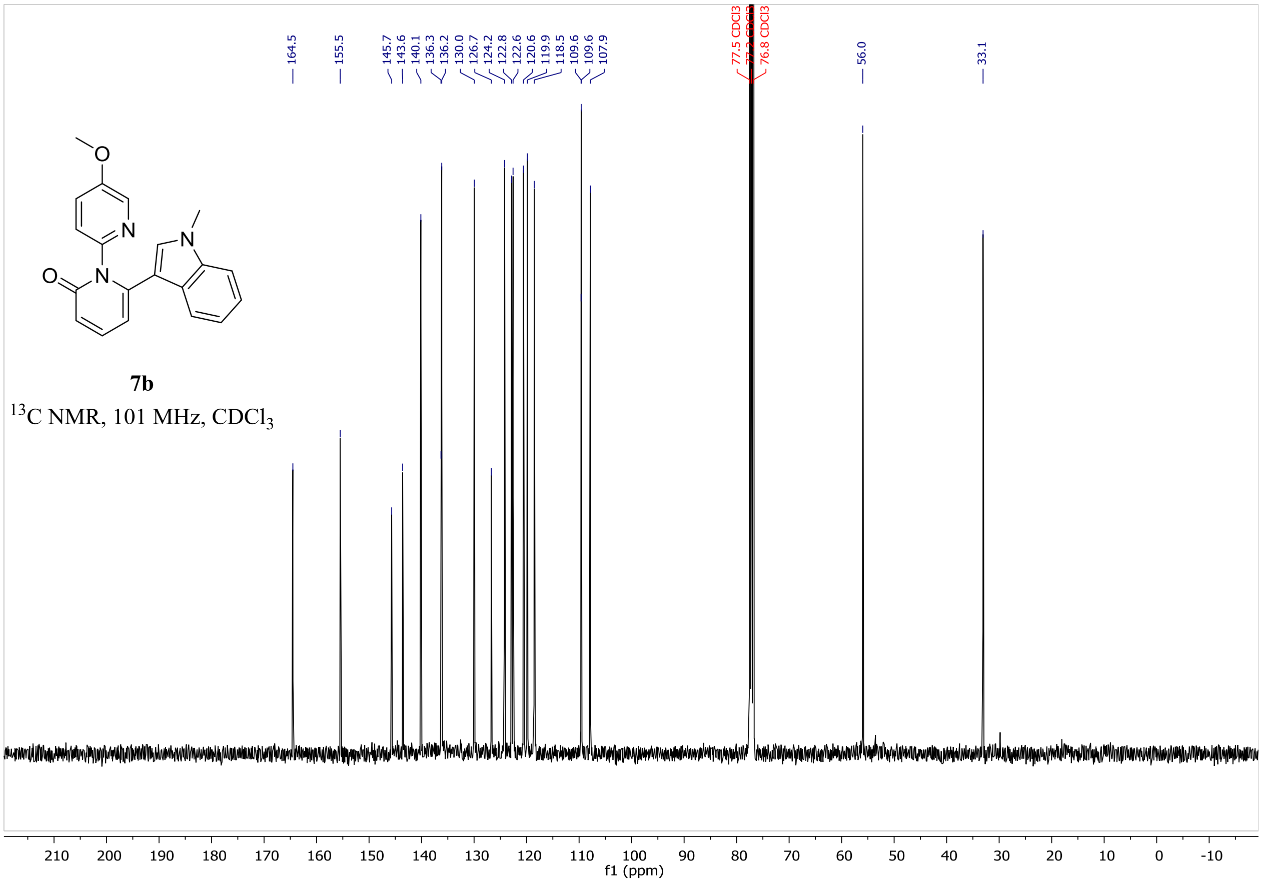

7b
^{13}C NMR, 101 MHz, $CDCl_3$

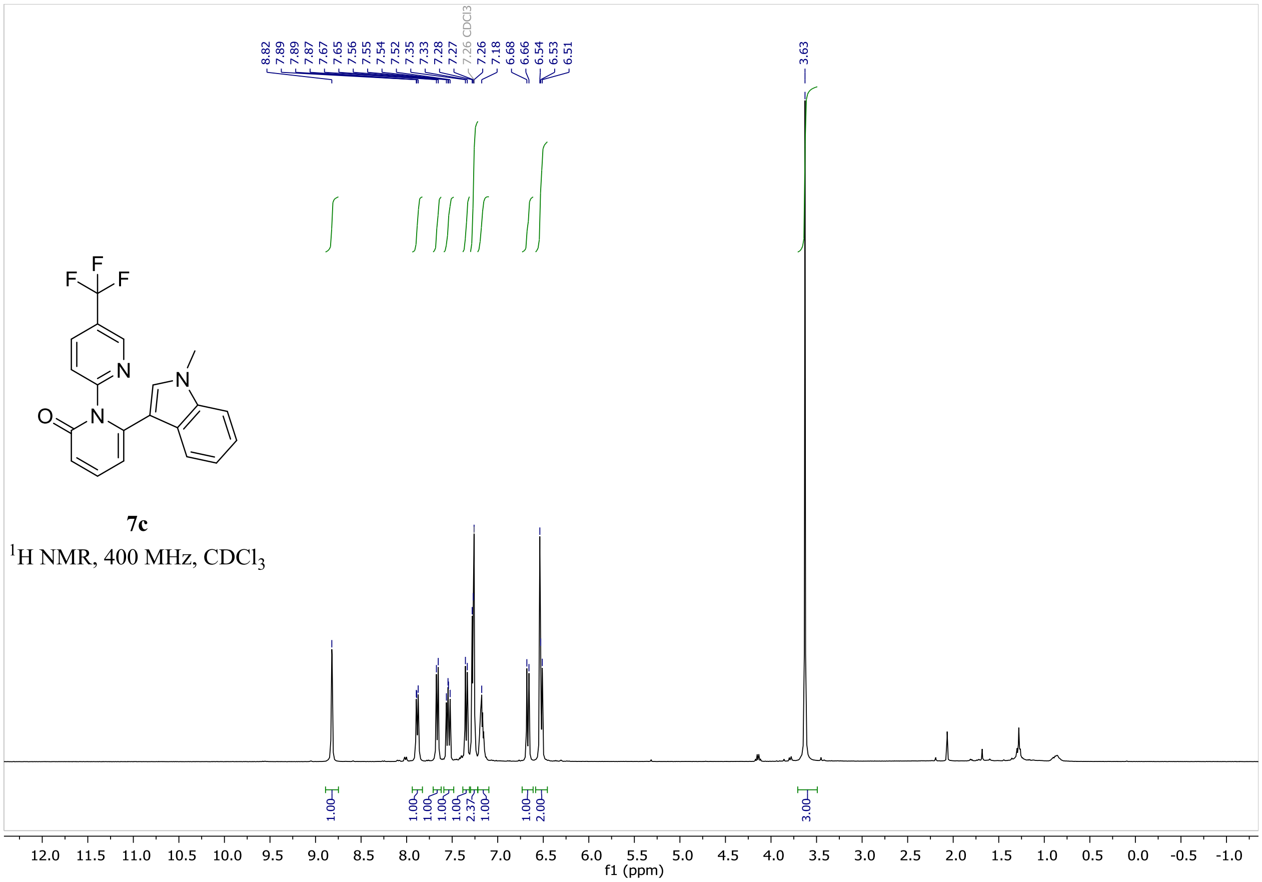
8.82
7.89
7.89
7.87
7.67
7.65
7.56
7.55
7.54
7.52
7.35
7.33
7.28
7.27
7.26 CDCl3
7.26
7.18
6.68
6.66
6.54
6.53
6.51
3.63
F
F
F
N
N
N
O
7c
^{1}H NMR, 400 MHz, $CDCl_3$
1.00
1.00
1.00
1.00
1.00
2.37
1.00
1.00
2.00
3.00
12.0 11.5 11.0 10.5 10.0 9.5 9.0 8.5 8.0 7.5 7.0 6.5 6.0 5.5 5.0 4.5 4.0 3.5 3.0 2.5 2.0 1.5 1.0 0.5 0.0 -0.5 -1.0
f1 (ppm)

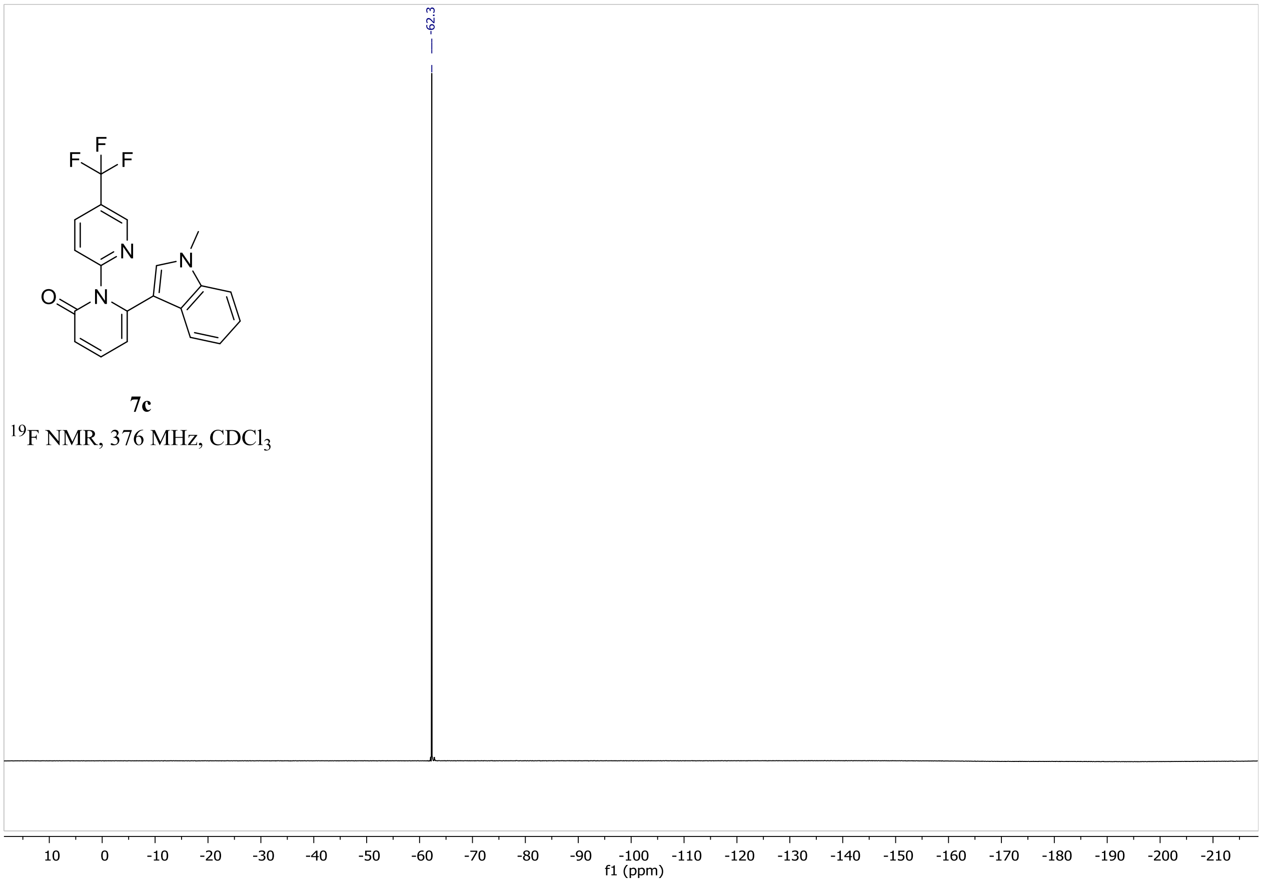
-62.3
F
F
F
N
N
N
O
7c
^{19}F NMR, 376 MHz, $CDCl_3$
10 0 -10 -20 -30 -40 -50 -60 -70 -80 -90 -100 -110 -120 -130 -140 -150 -160 -170 -180 -190 -200 -210
f1 (ppm)

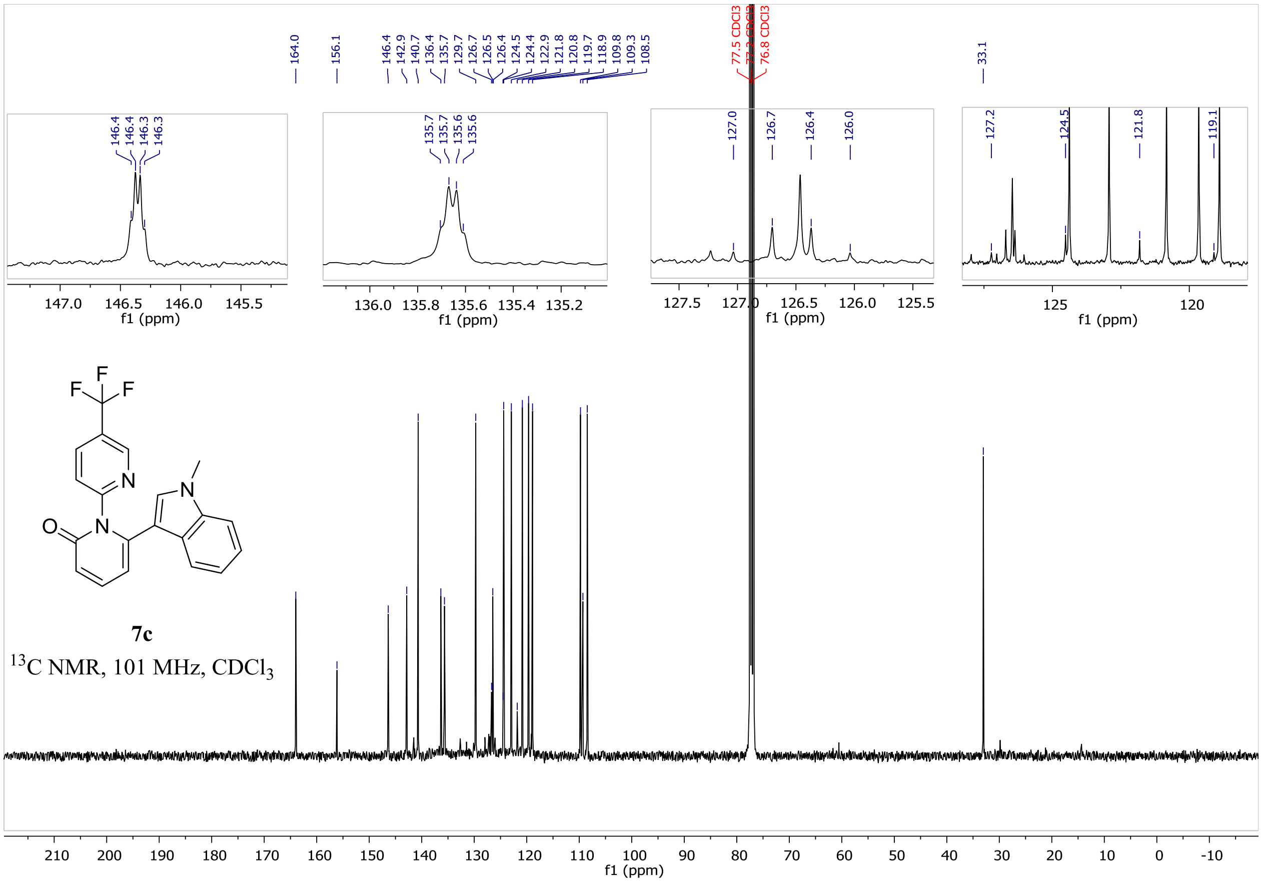
164.0
156.1
146.4
142.9
140.7
136.4
135.7
129.7
126.7
126.5
126.4
124.5
124.4
122.9
121.8
120.8
119.7
118.9
109.8
109.3
108.5
77.5 CDCl3
77.2 CDCl3
76.8 CDCl3
33.1
146.4 146.4 146.3 146.3
135.7 135.7 135.6 135.6
127.0 126.7 126.4 126.0
127.2 124.5 121.8 119.1
f1 (ppm)
7c
^{13}C NMR, 101 MHz, $CDCl_3$

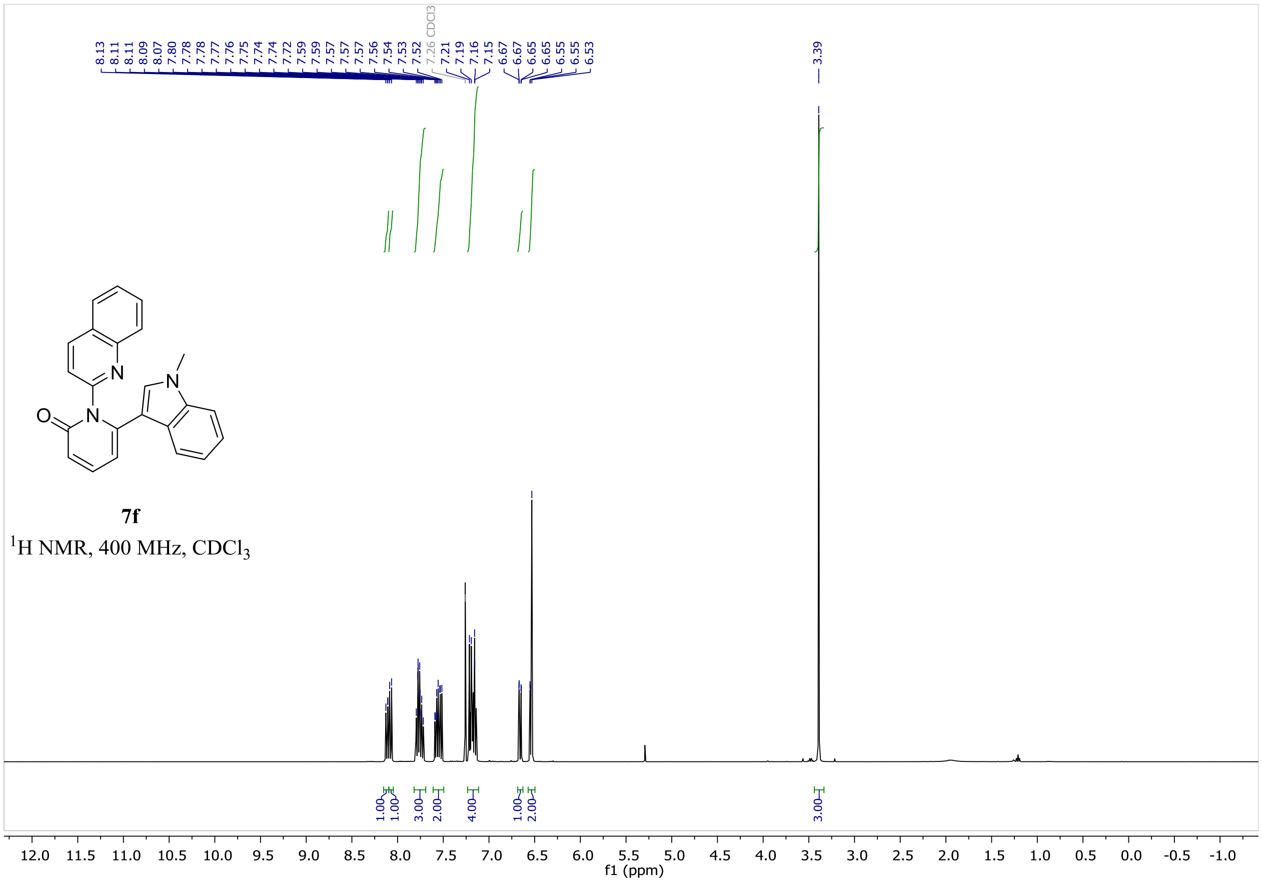

7f

^{1}H NMR, 400 MHz, $CDCl_3$

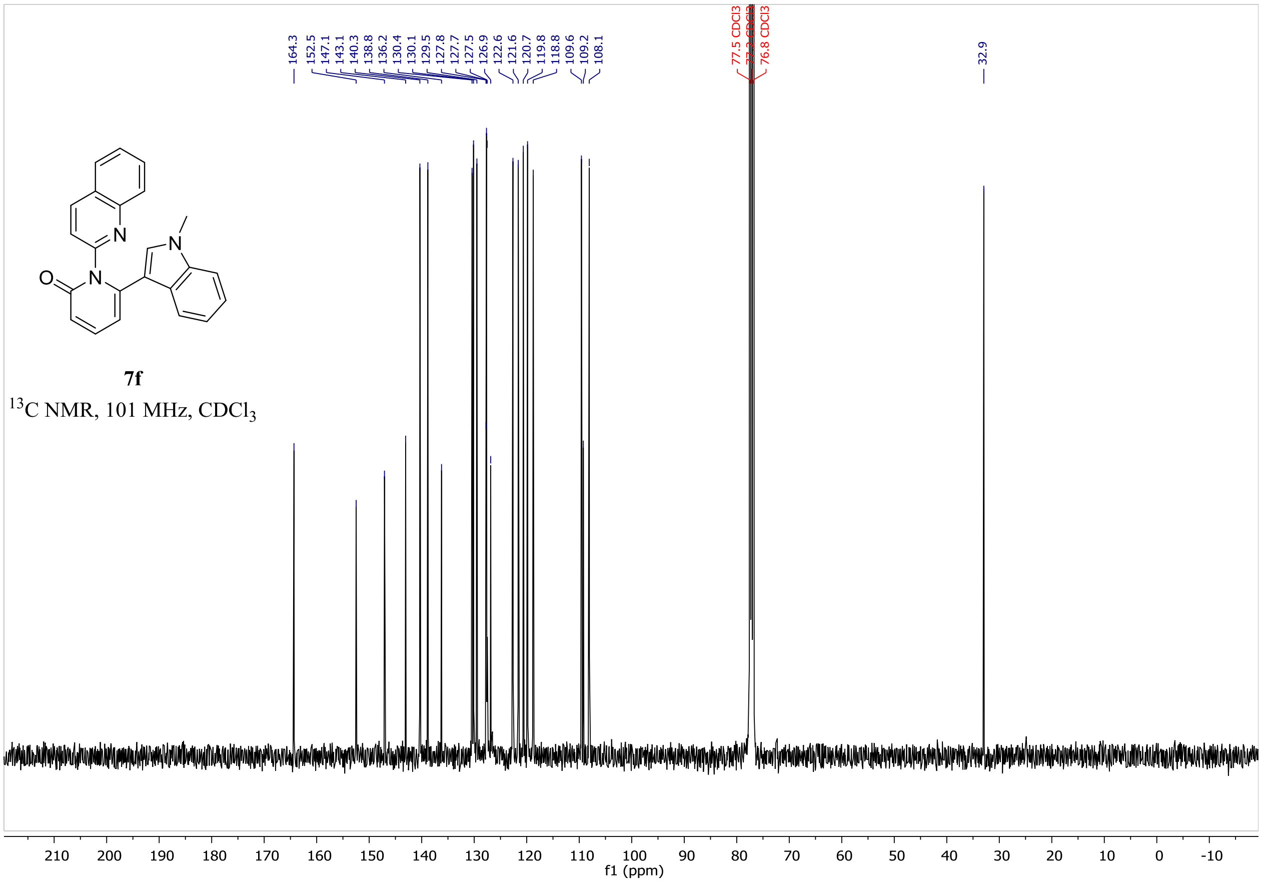

7f

^{13}C NMR, 101 MHz, $CDCl_3$

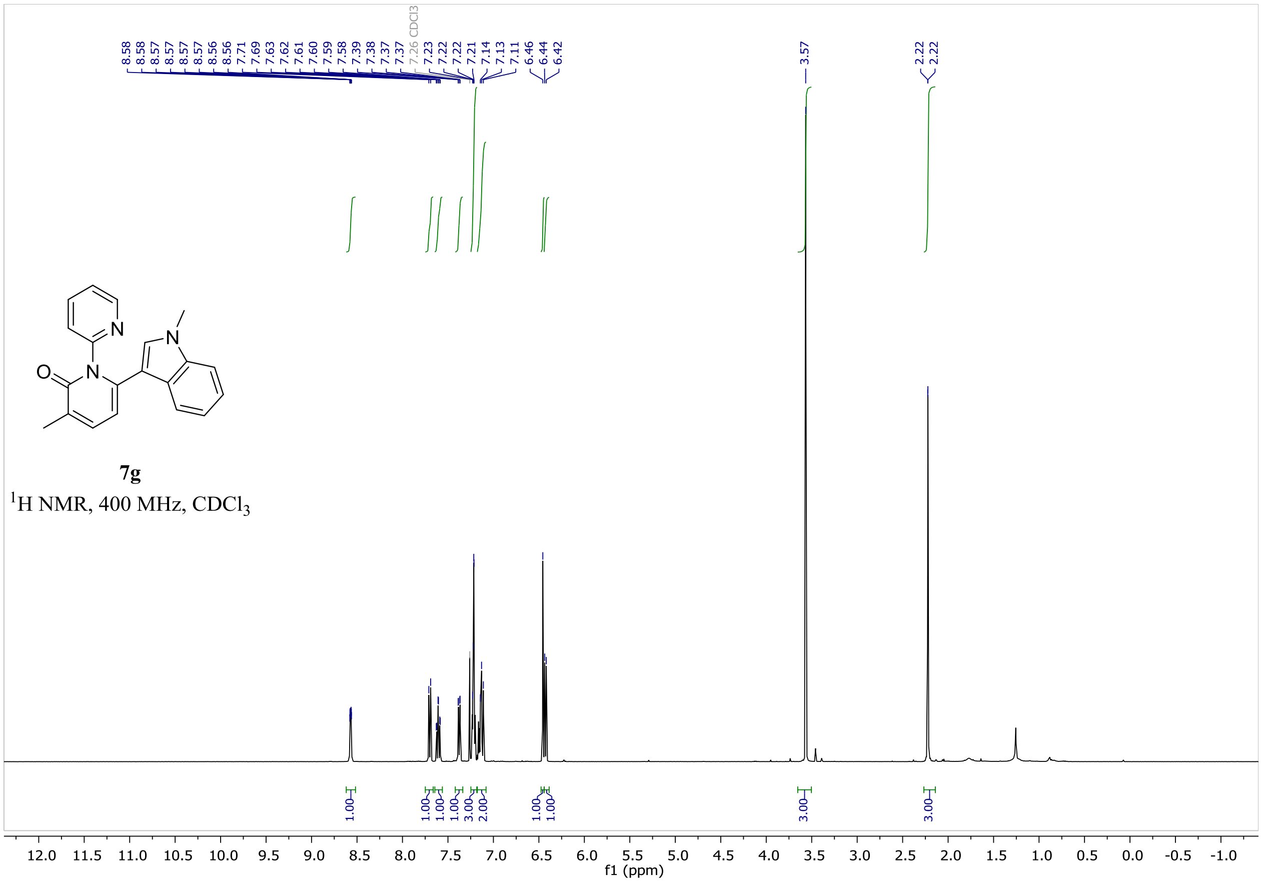

7g

^{1}H NMR, 400 MHz, $CDCl_3$

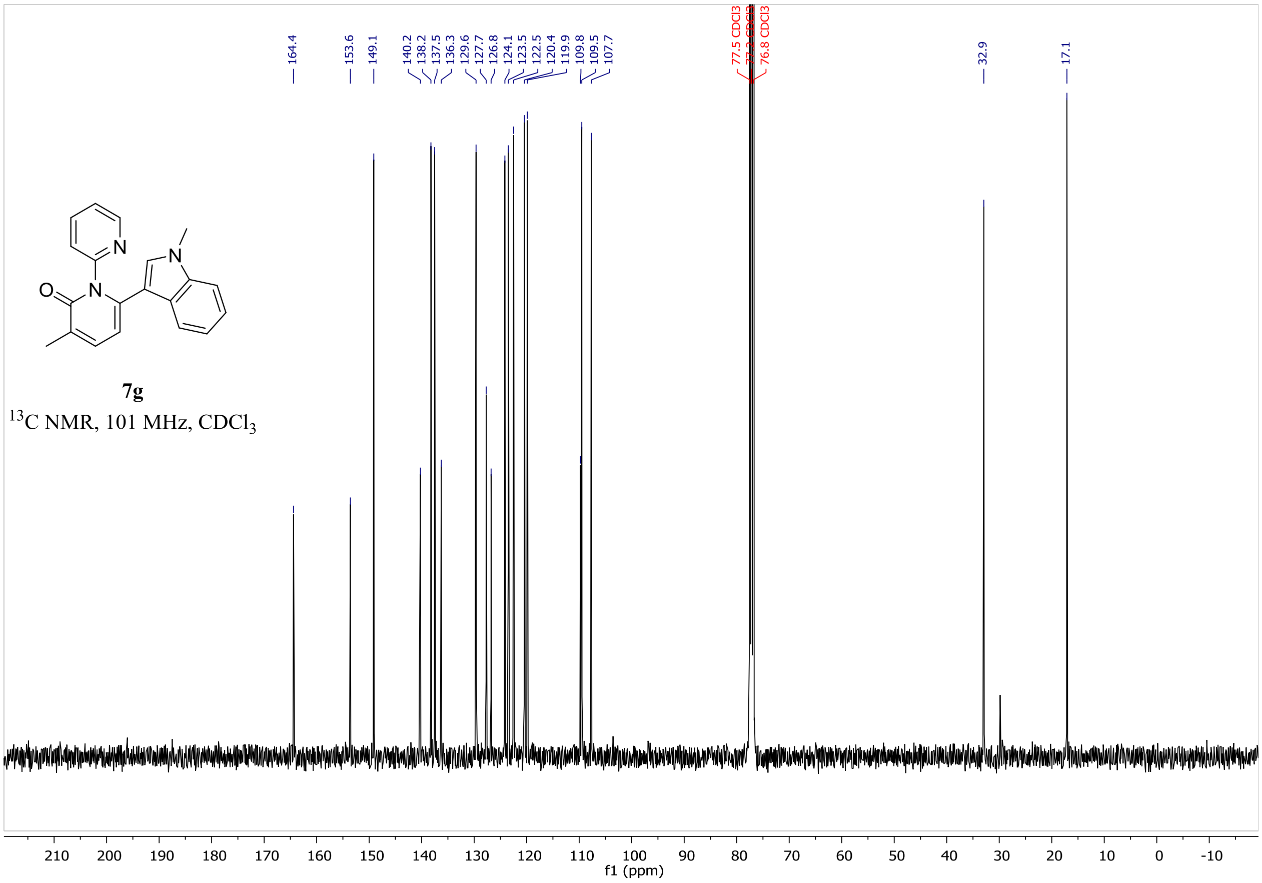

7g

^{13}C NMR, 101 MHz, $CDCl_3$

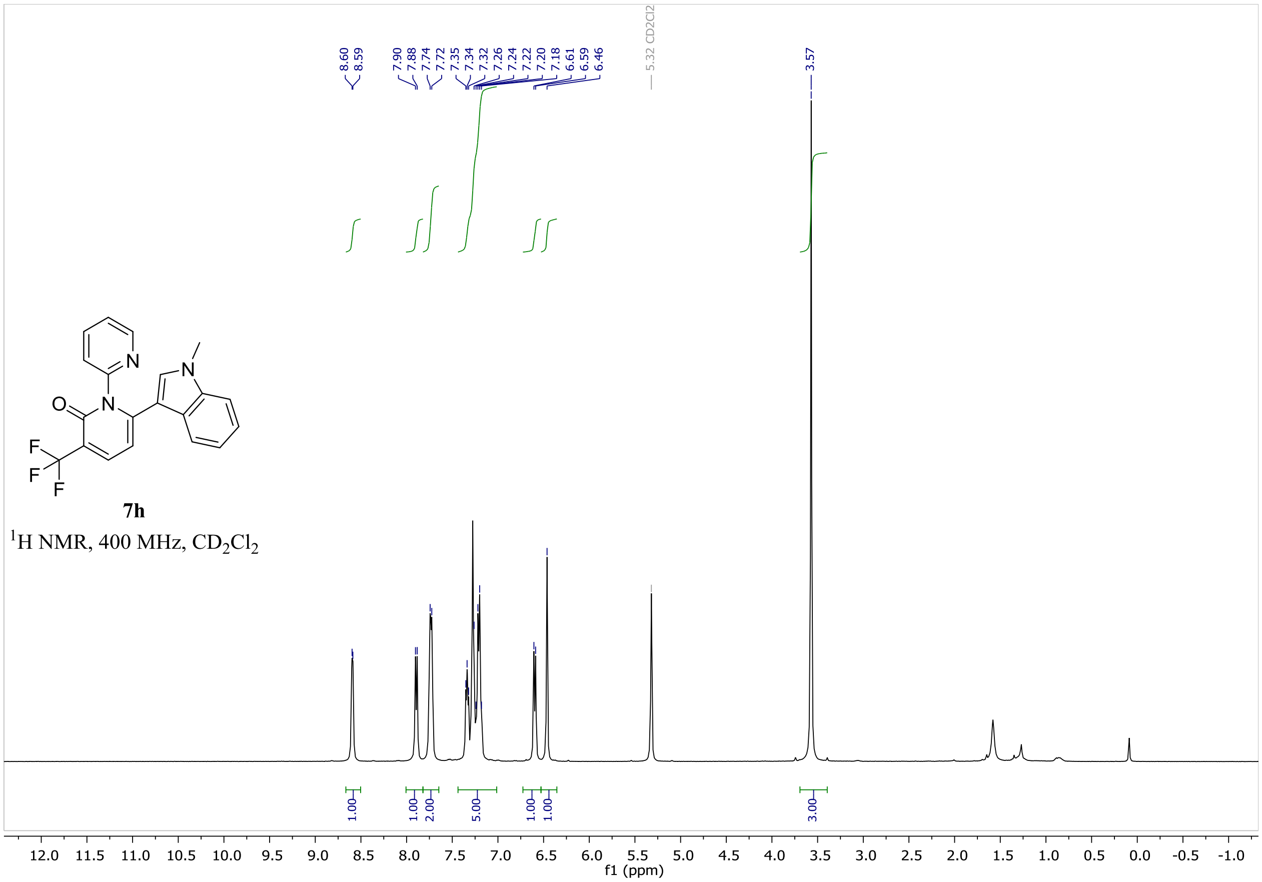
8.60
8.59
7.90
7.88
7.74
7.72
7.35
7.34
7.32
7.26
7.24
7.22
7.20
7.18
6.61
6.59
6.46
5.32 CD2Cl2
3.57
7h
^{1}H NMR, 400 MHz, CD_2Cl_2
1.00
1.00
2.00
5.00
1.00
1.00
3.00
12.0 11.5 11.0 10.5 10.0 9.5 9.0 8.5 8.0 7.5 7.0 6.5 6.0 5.5 5.0 4.5 4.0 3.5 3.0 2.5 2.0 1.5 1.0 0.5 0.0 -0.5 -1.0
f1 (ppm)

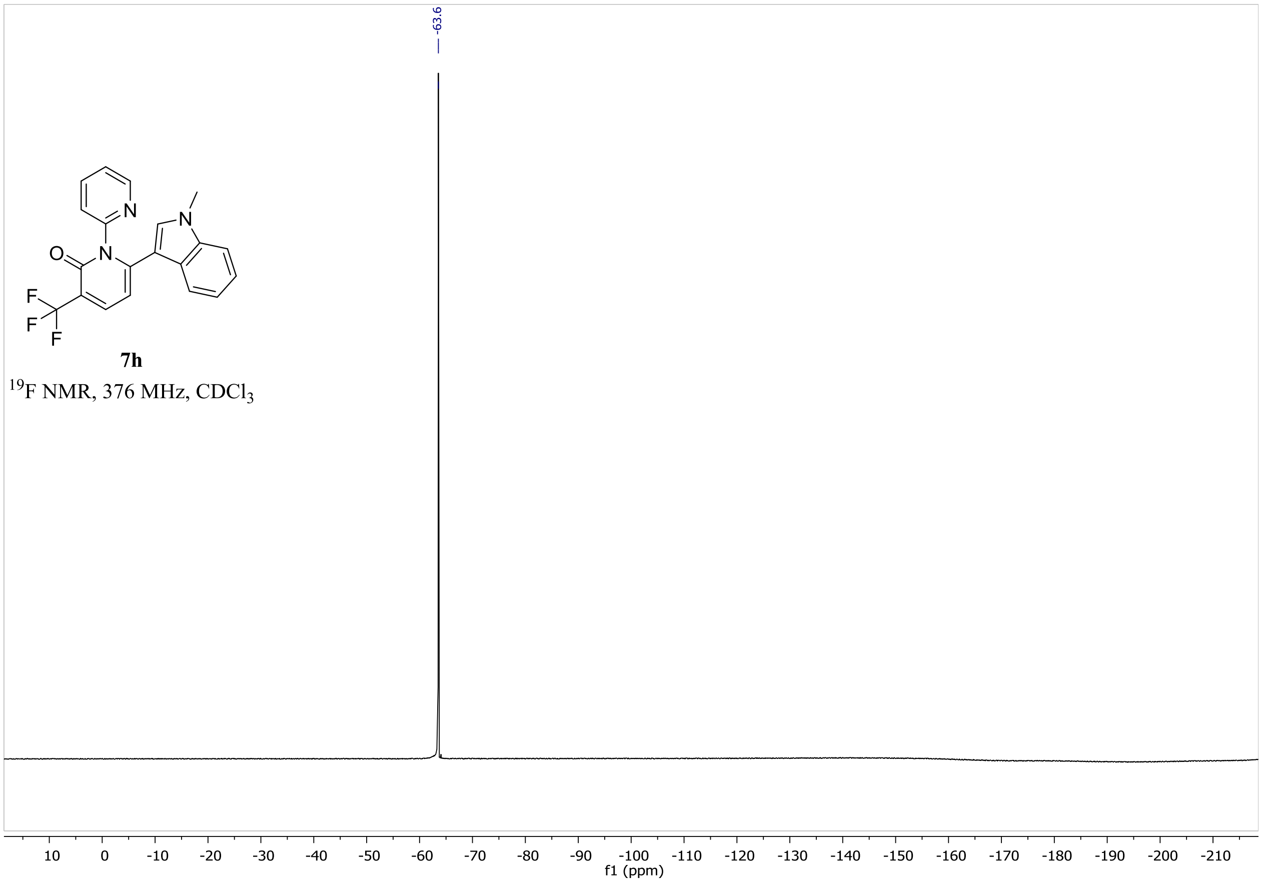
-63.6
7h
^{19}F NMR, 376 MHz, $CDCl_3$
10 0 -10 -20 -30 -40 -50 -60 -70 -80 -90 -100 -110 -120 -130 -140 -150 -160 -170 -180 -190 -200 -210
f1 (ppm)

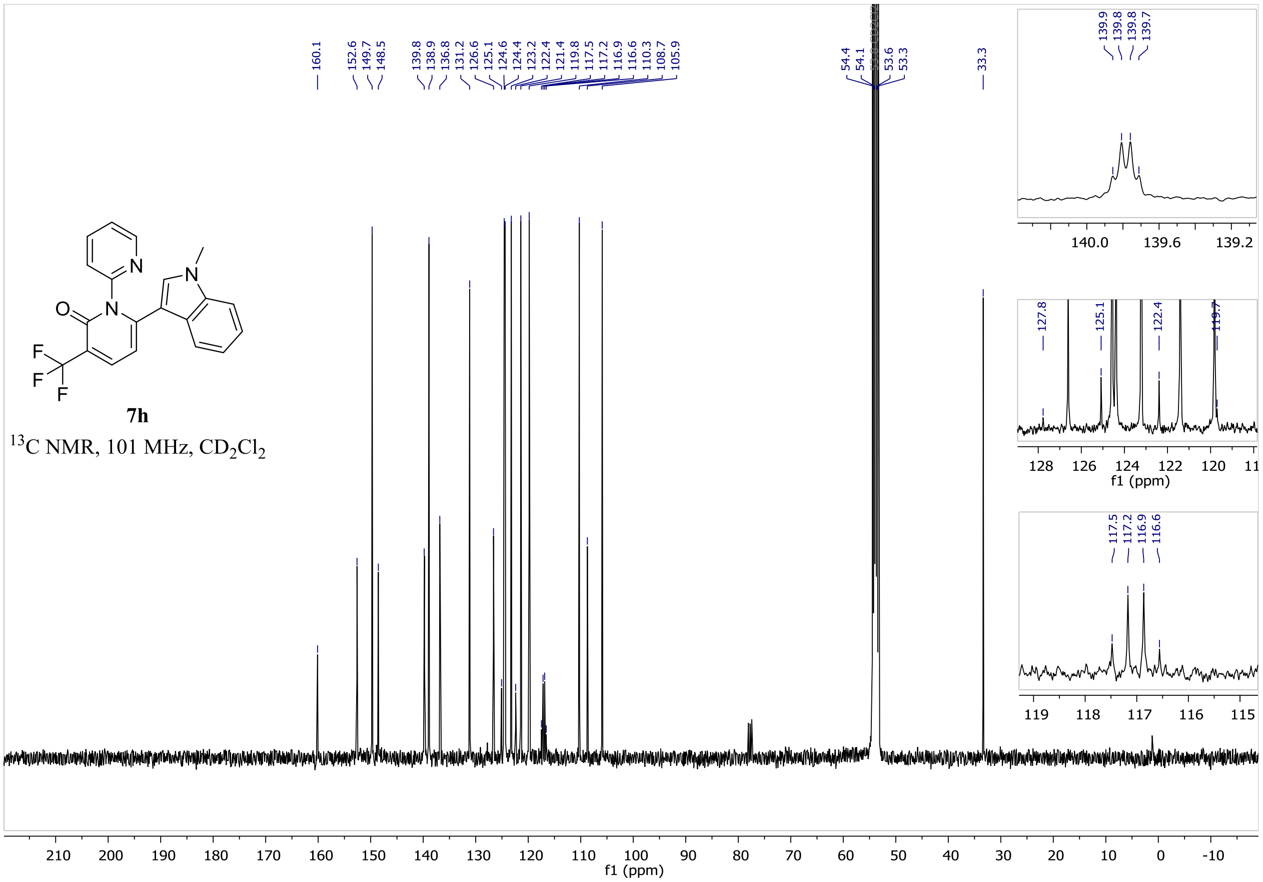

7h

^{13}C NMR, 101 MHz, CD_2Cl_2

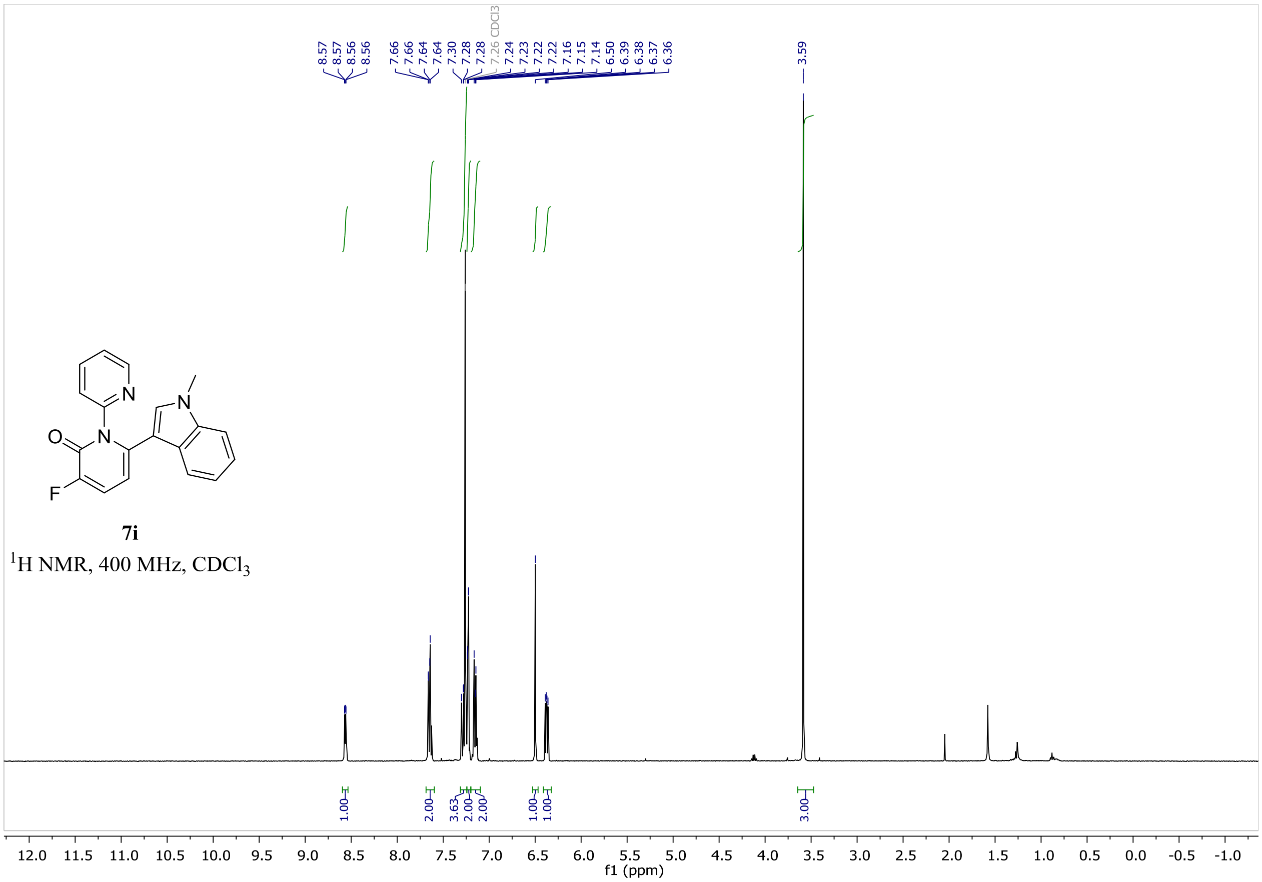
8.57 8.57 8.56 8.56 7.66 7.66 7.64 7.64 7.30 7.28 7.28 7.26 CDCl3 7.24 7.23 7.22 7.22 7.22 7.16 7.15 7.14 6.50 6.39 6.38 6.37 6.36
3.59
1.00 2.00 3.63 2.00 2.00 1.00 1.00 3.00
7i
1H NMR, 400 MHz, CDCl3
f1 (ppm)

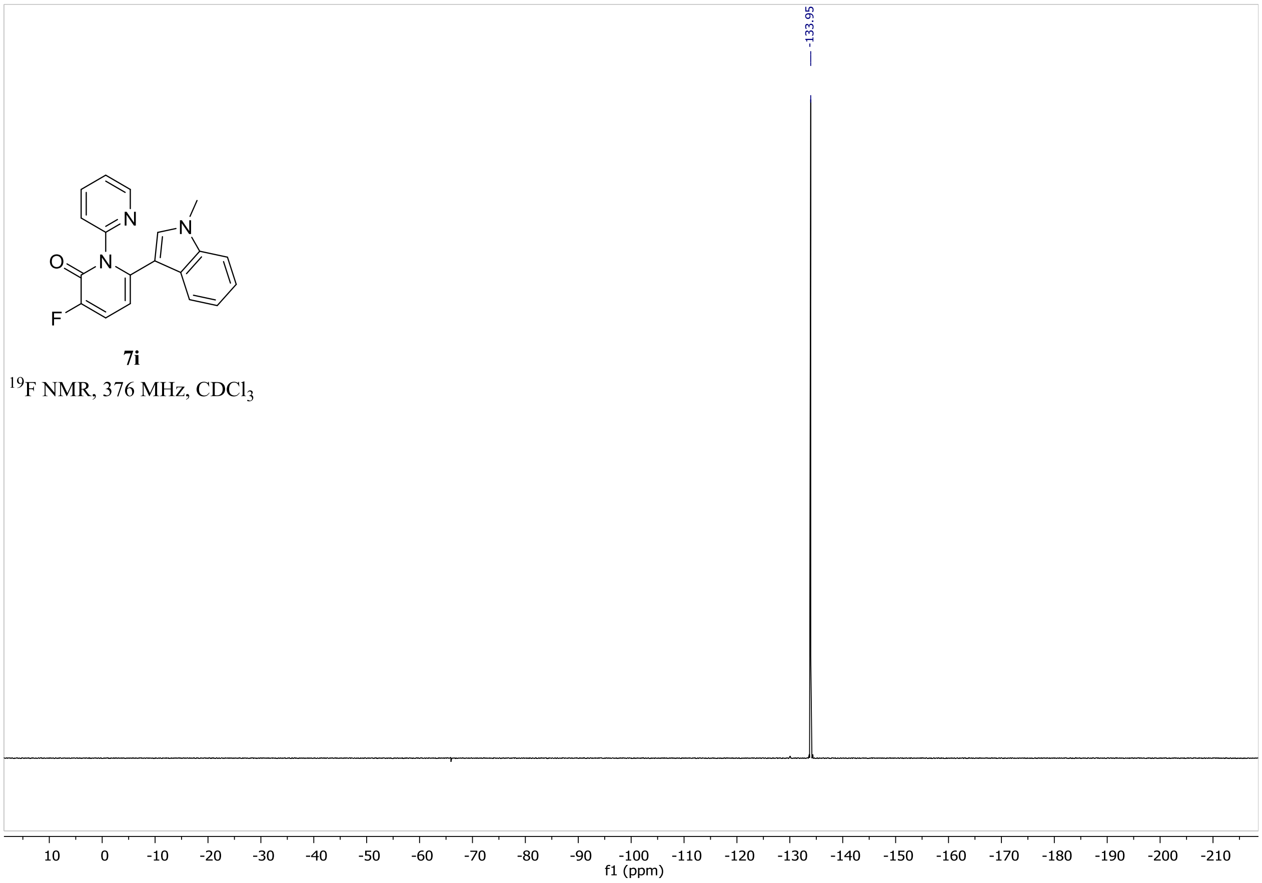
-133.95
7i
19F NMR, 376 MHz, CDCl3
f1 (ppm)

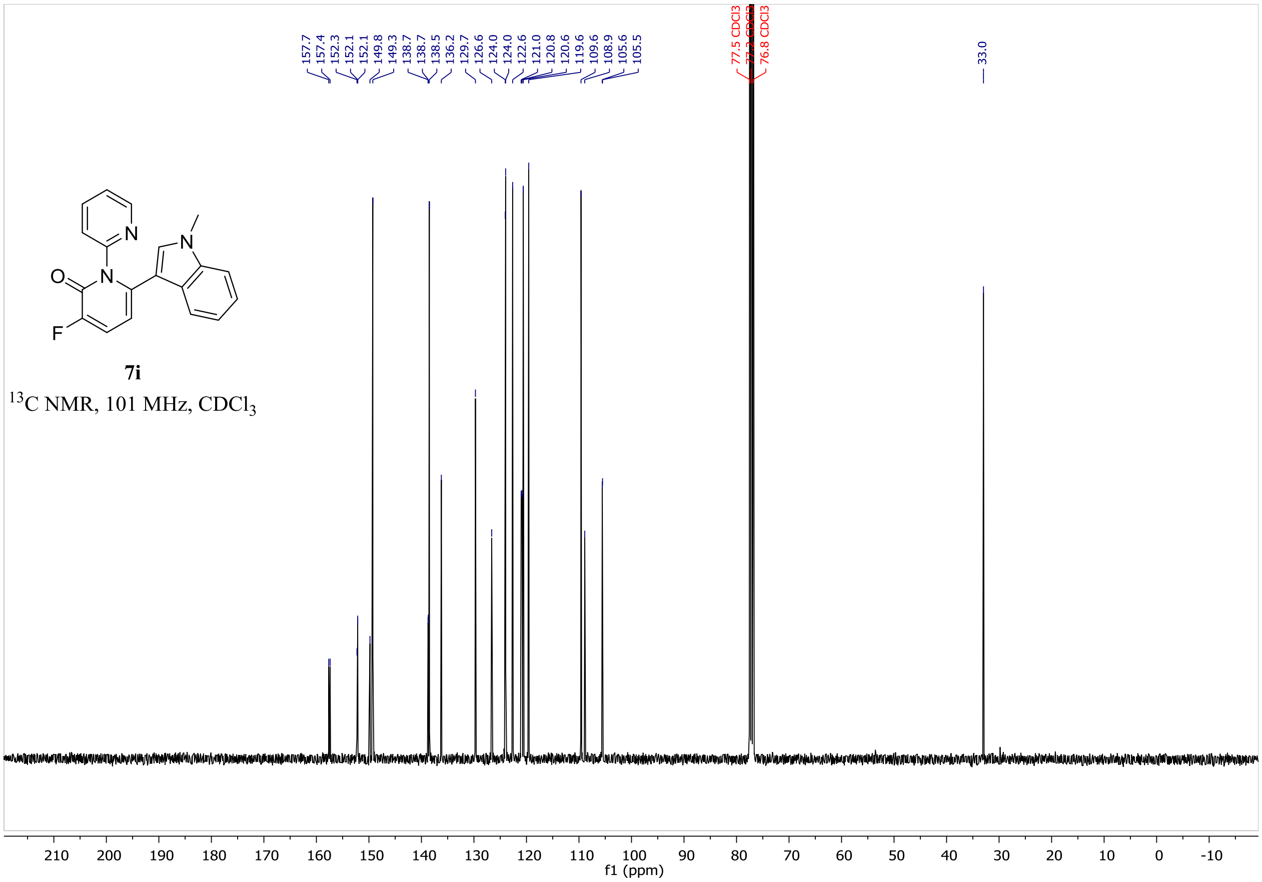

7i

^{13}C NMR, 101 MHz, $CDCl_3$

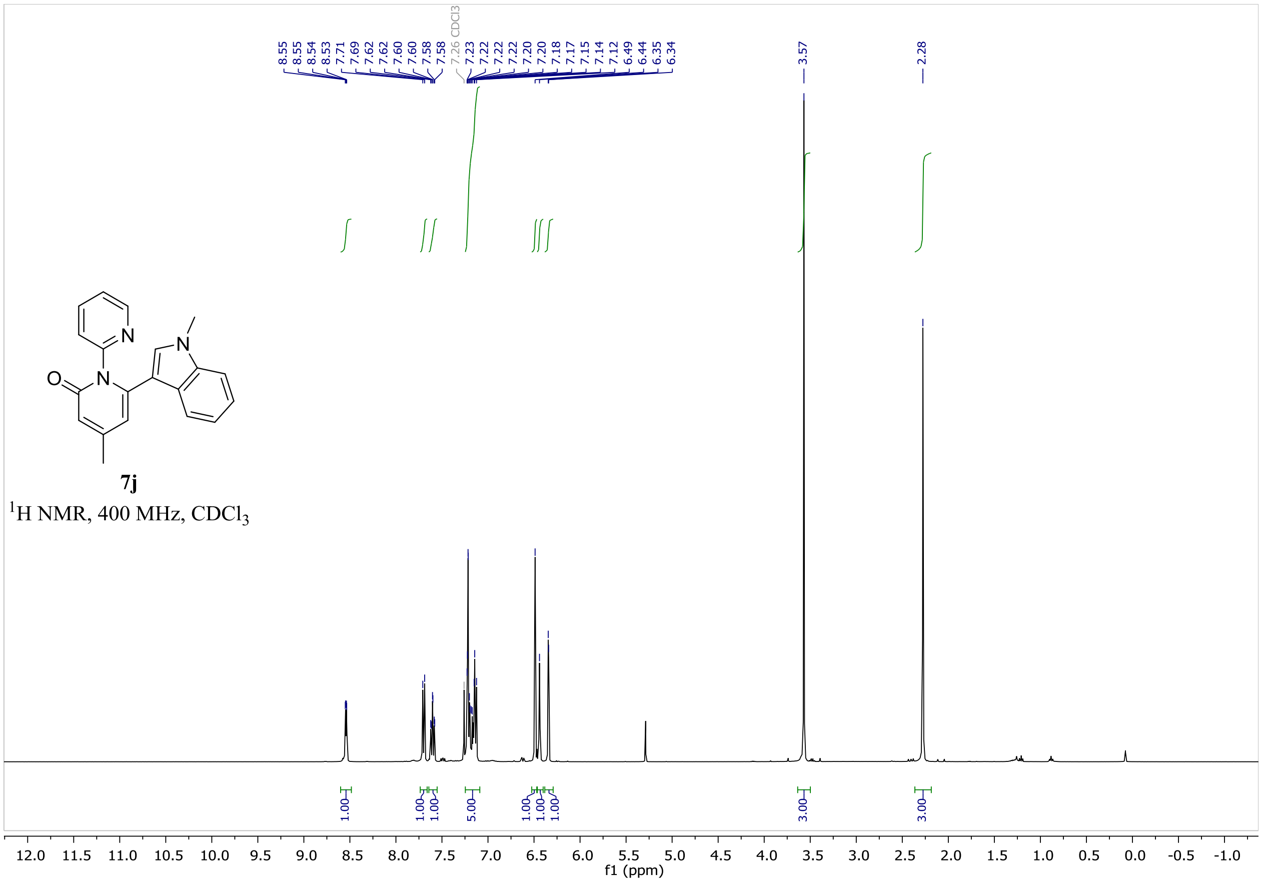

^{1}H NMR, 400 MHz, $CDCl_3$

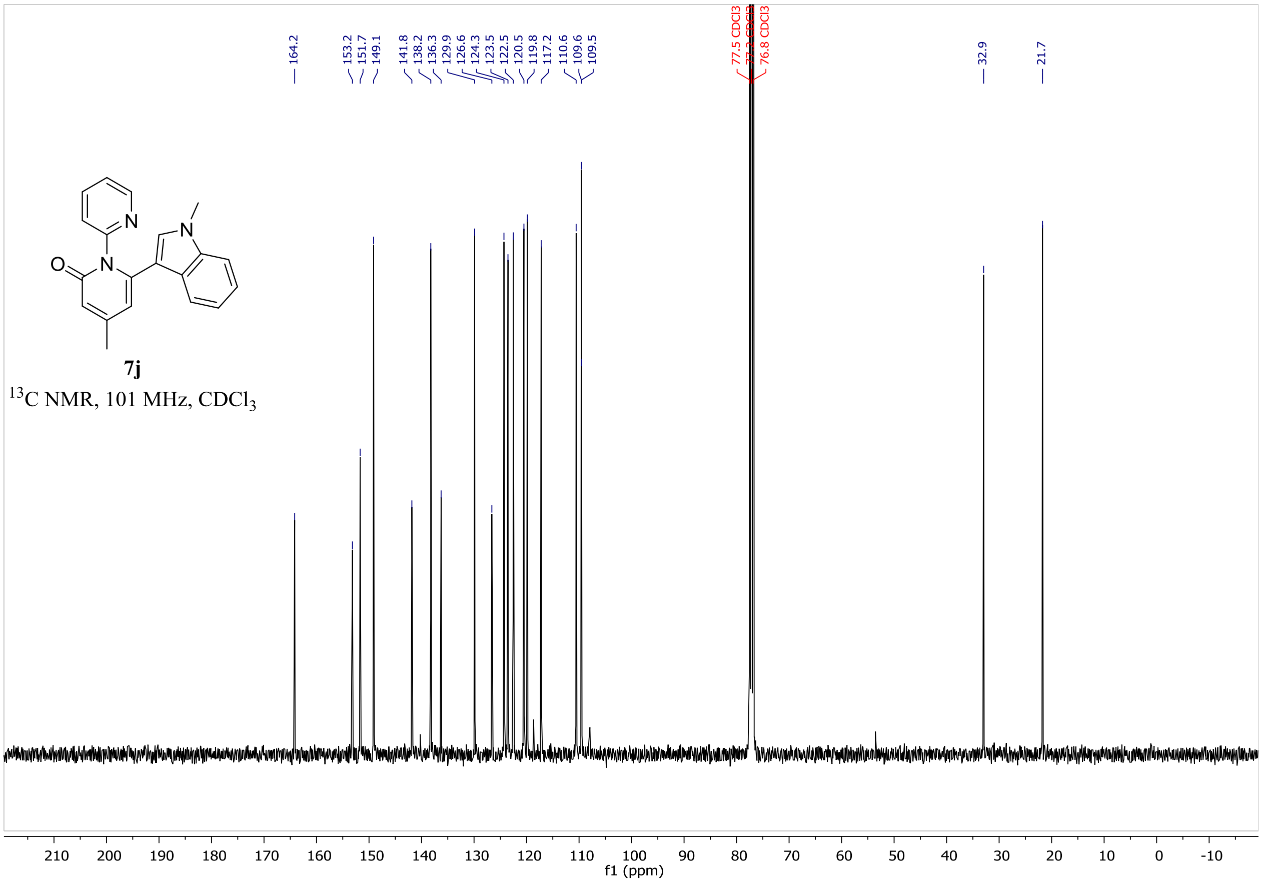

^{13}C NMR, 101 MHz, $CDCl_3$

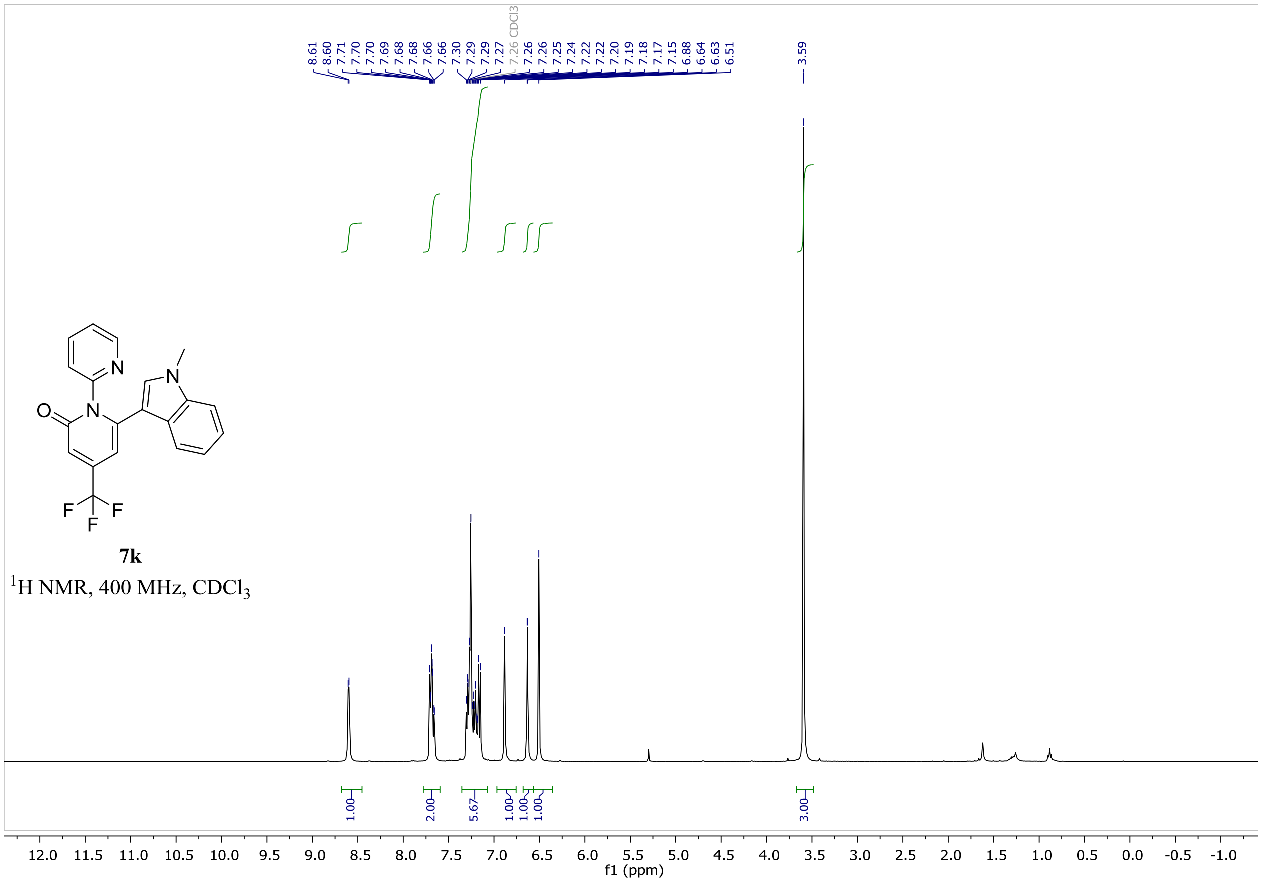

7k

^{1}H NMR, 400 MHz, $CDCl_3$

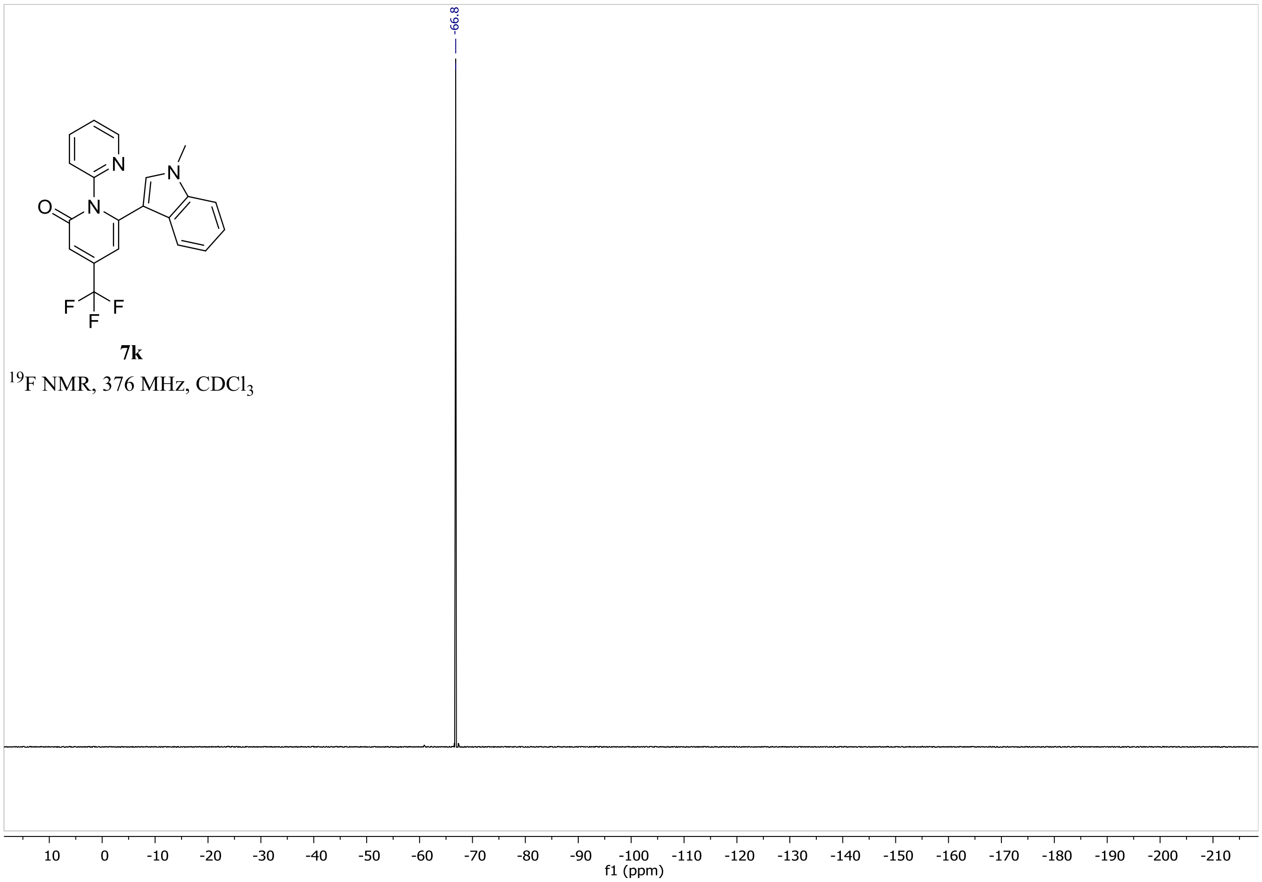

7k

^{19}F NMR, 376 MHz, $CDCl_3$

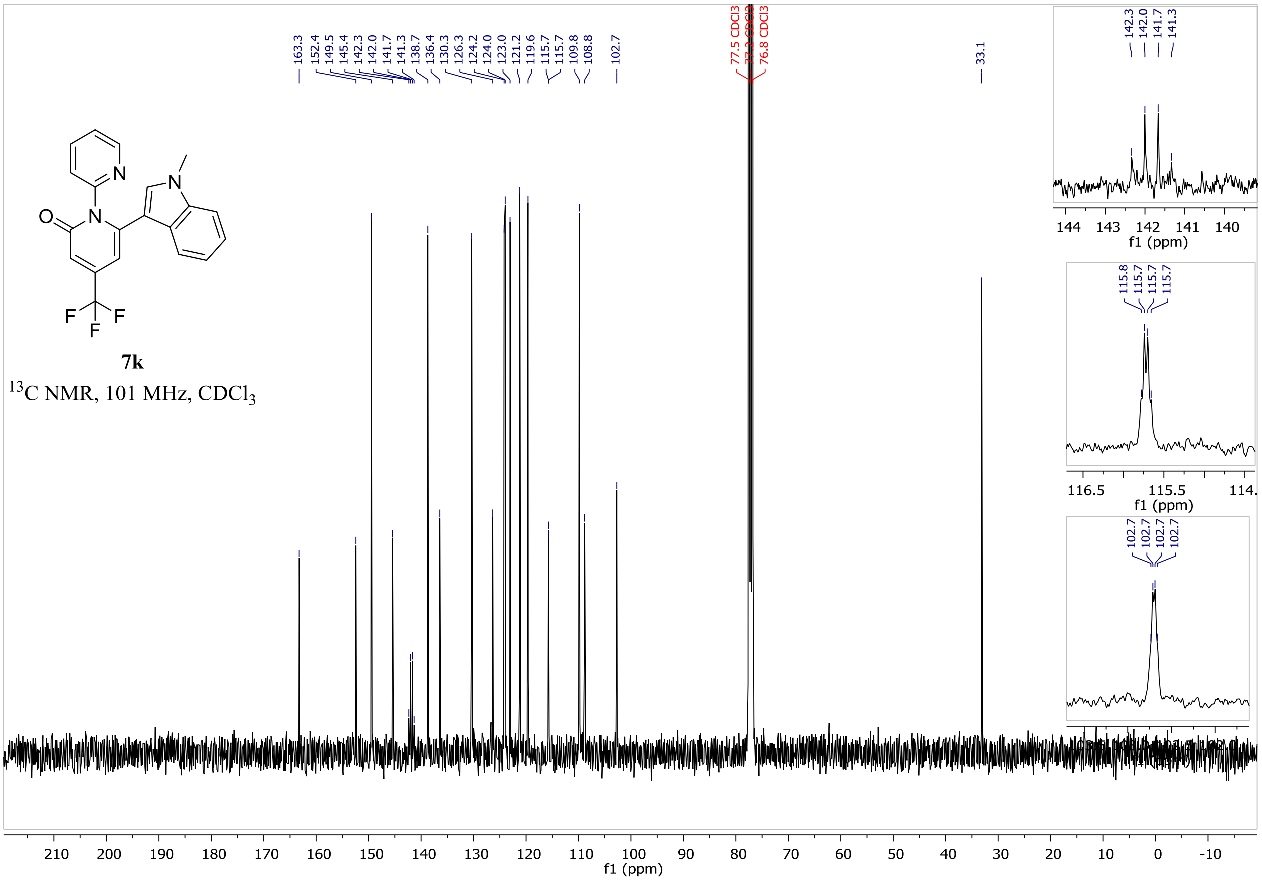

7k

^{13}C NMR, 101 MHz, $CDCl_3$

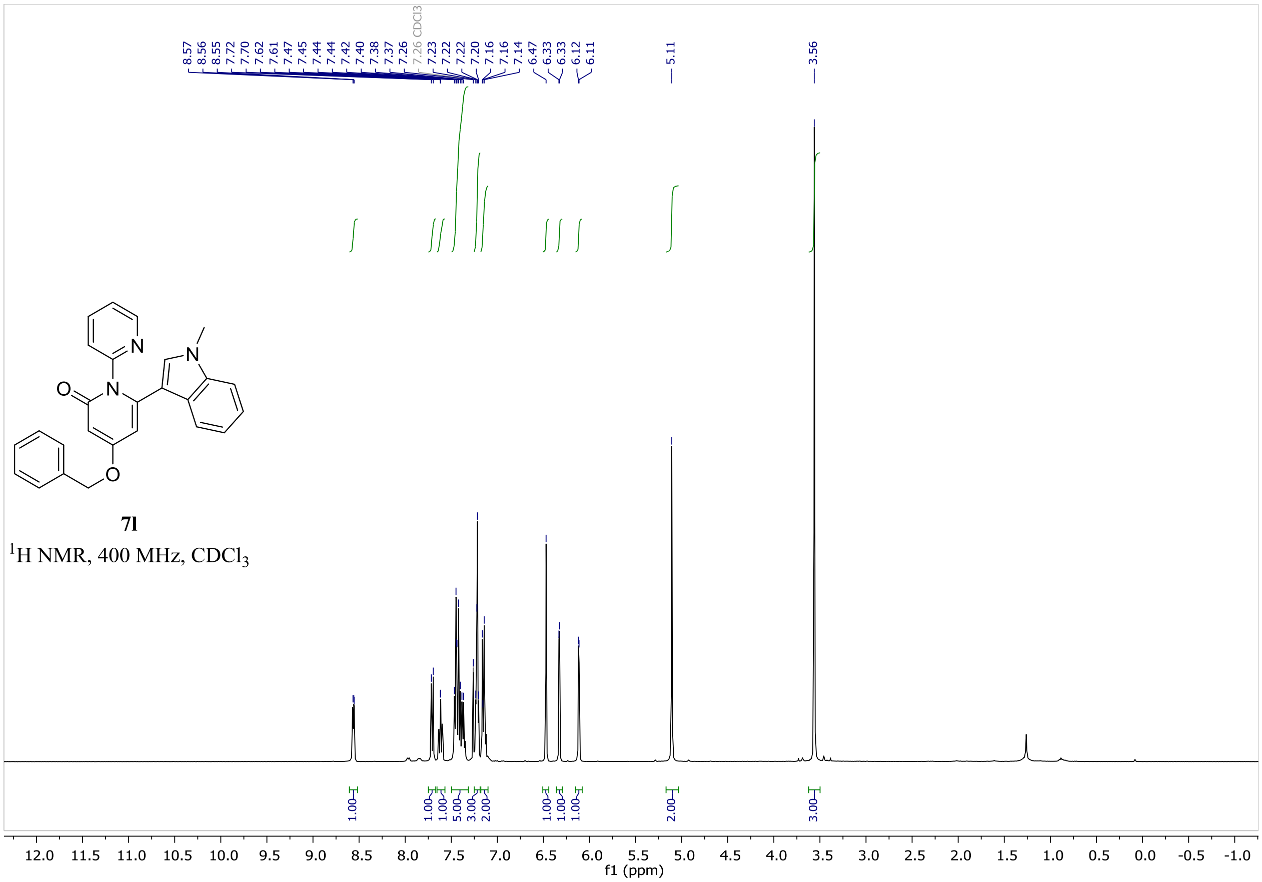

7l

^{1}H NMR, 400 MHz, $CDCl_3$

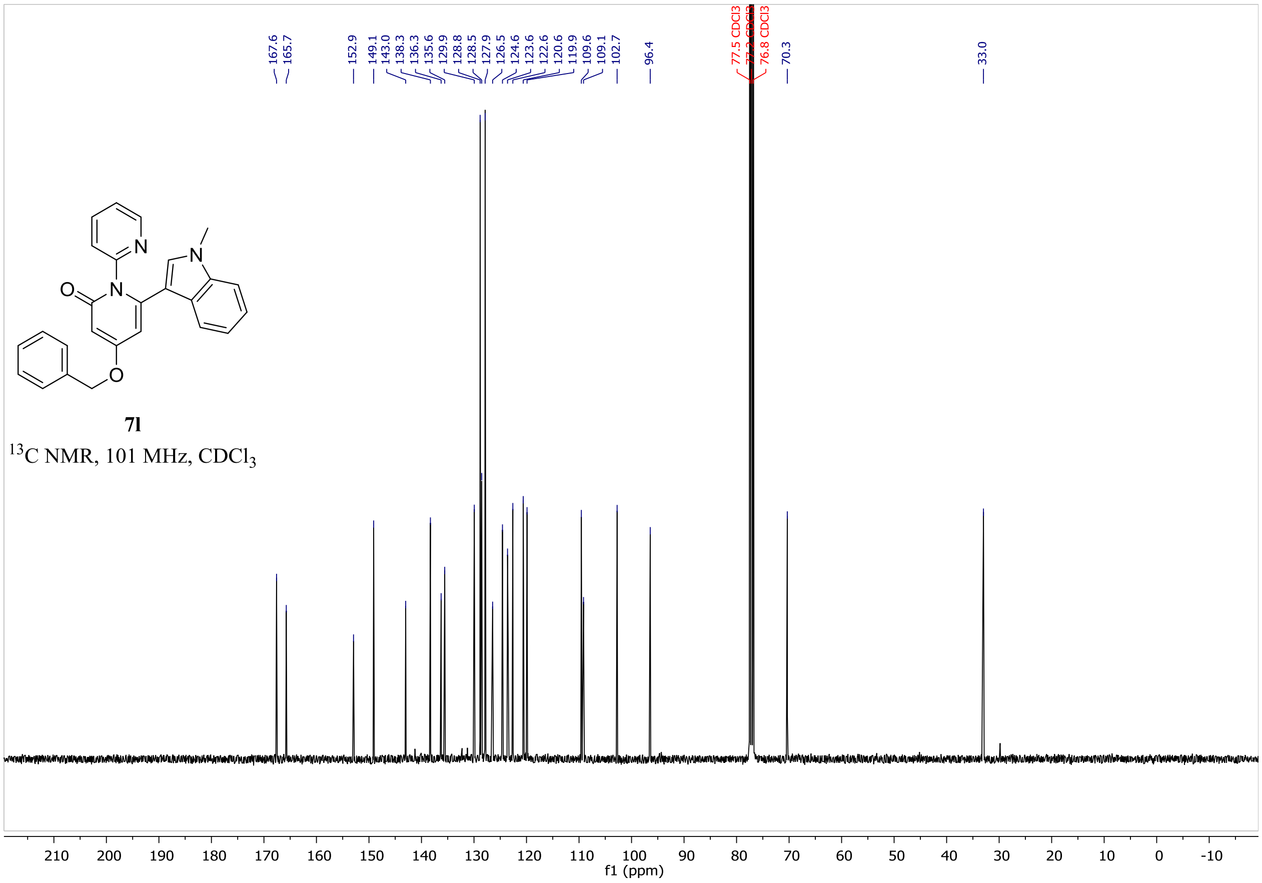

7l

^{13}C NMR, 101 MHz, $CDCl_3$

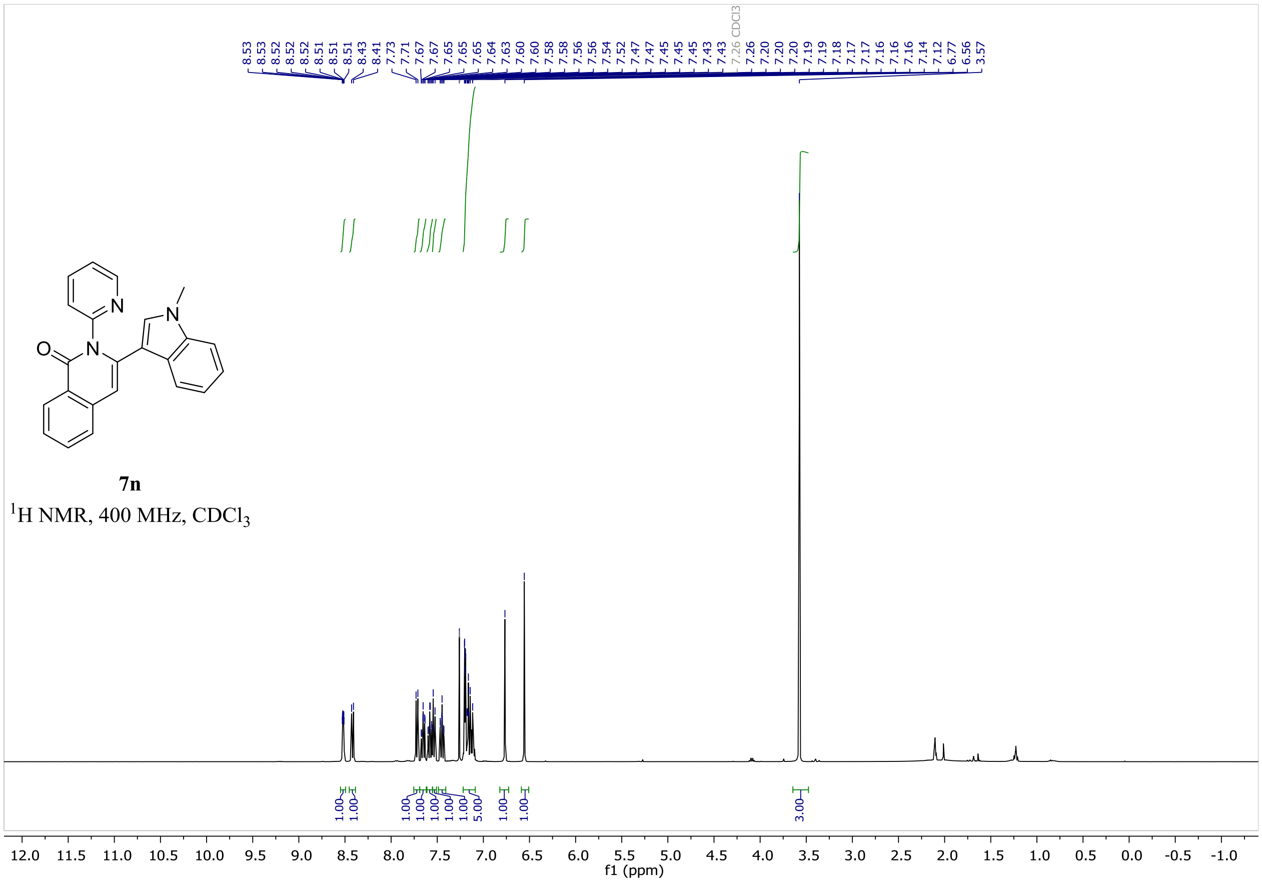
8.53 8.53 8.52 8.52 8.52 8.51 8.51 8.51 8.43 8.41 7.73 7.71 7.67 7.67 7.65 7.65 7.65 7.64 7.63 7.60 7.60 7.58 7.58 7.56 7.56 7.54 7.52 7.47 7.47 7.45 7.45 7.45 7.43 7.43 7.26 CDCl3 7.26 7.20 7.20 7.20 7.20 7.19 7.19 7.18 7.17 7.17 7.16 7.16 7.14 7.12 6.77 6.56 3.57
1.00 1.00 1.00 1.00 1.00 1.00 1.00 5.00 1.00 1.00 3.00
N
O
N
N
7n
1H NMR, 400 MHz, CDCl3
f1 (ppm)

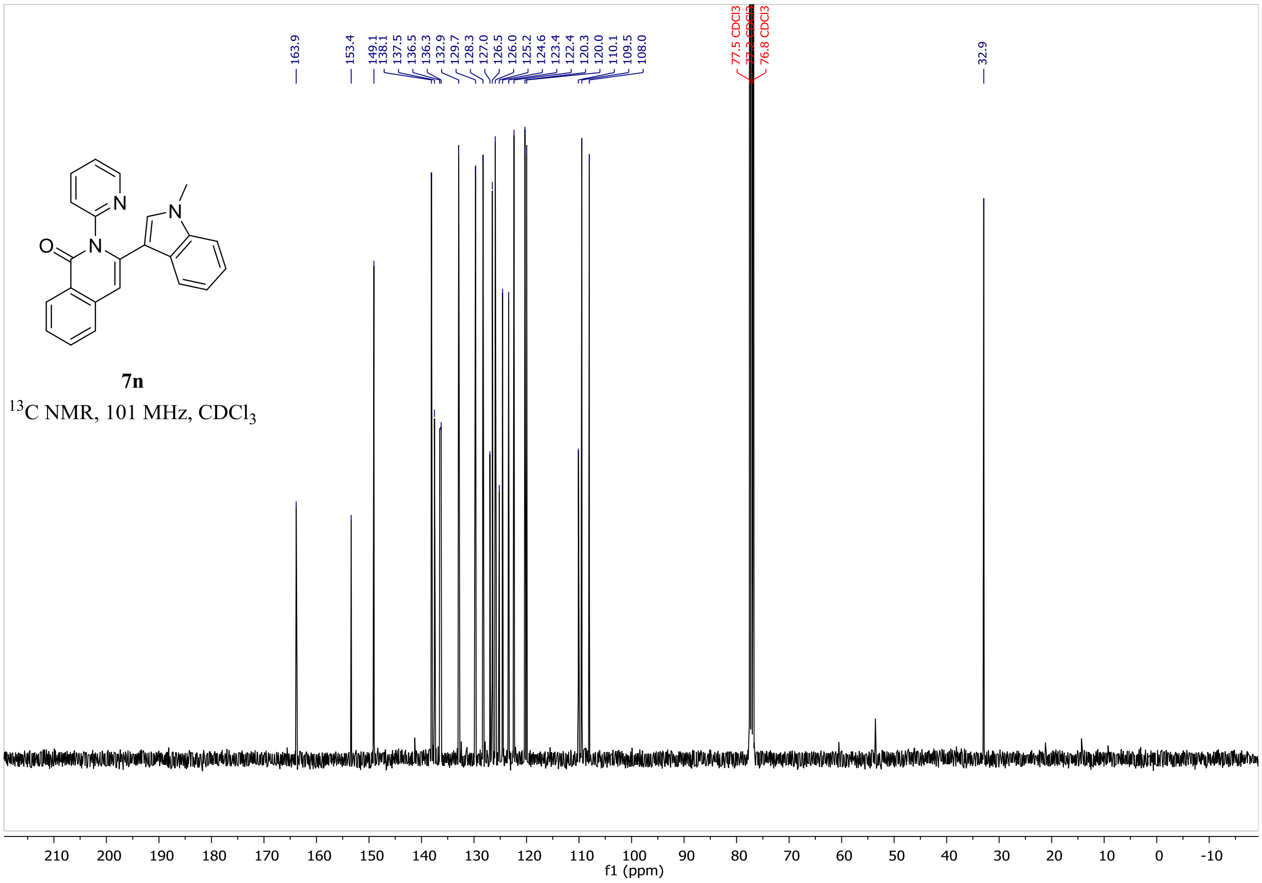
163.9
153.4
149.1 138.1 137.5 136.5 136.3 132.9 129.7 128.3 127.0 126.5 126.0 125.2 124.6 123.4 122.4 120.3 120.0 110.1 109.5 108.0
77.5 CDCl3
76.8 CDCl3
32.9
N
O
N
N
7n
13C NMR, 101 MHz, CDCl3
f1 (ppm)

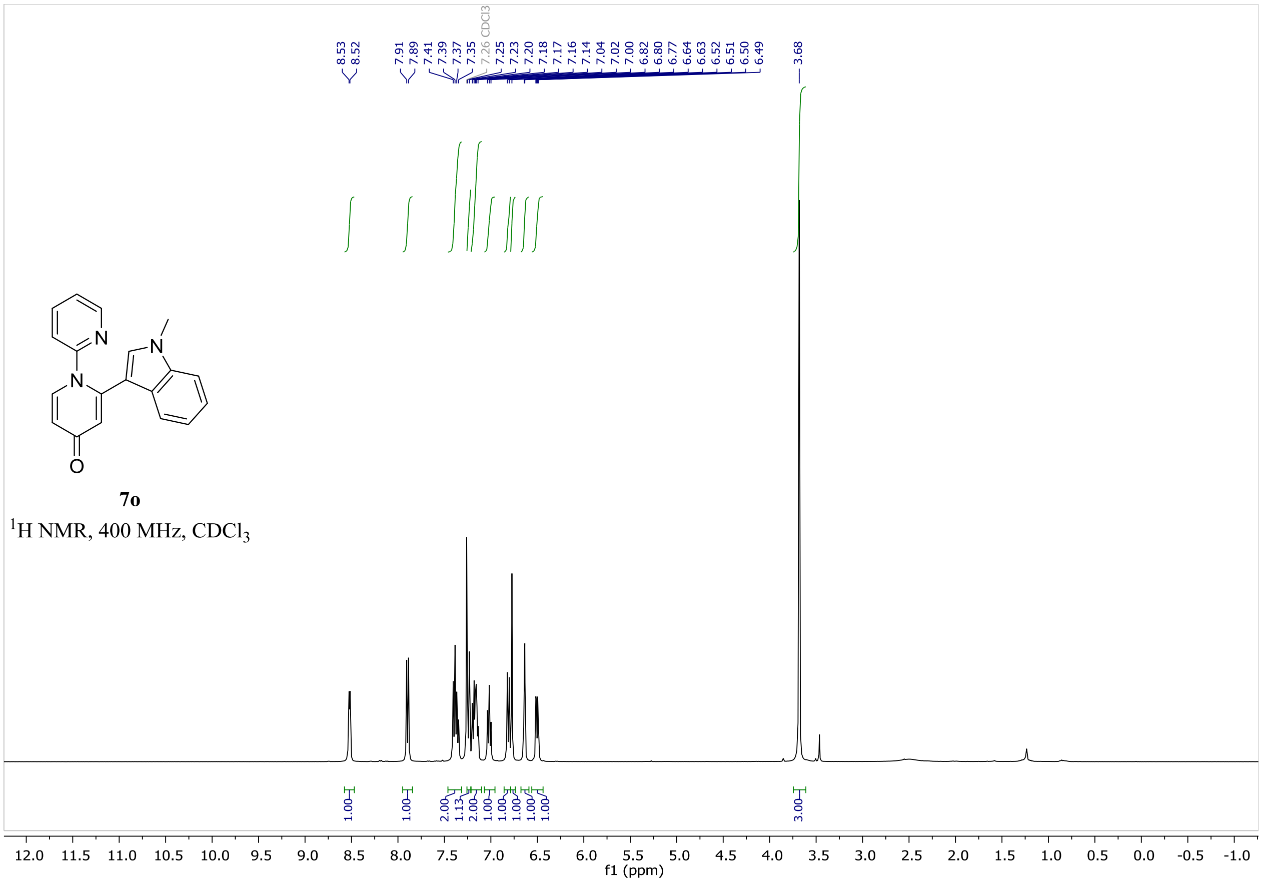

7o
^1H NMR, 400 MHz, $CDCl_3$
f1 (ppm)

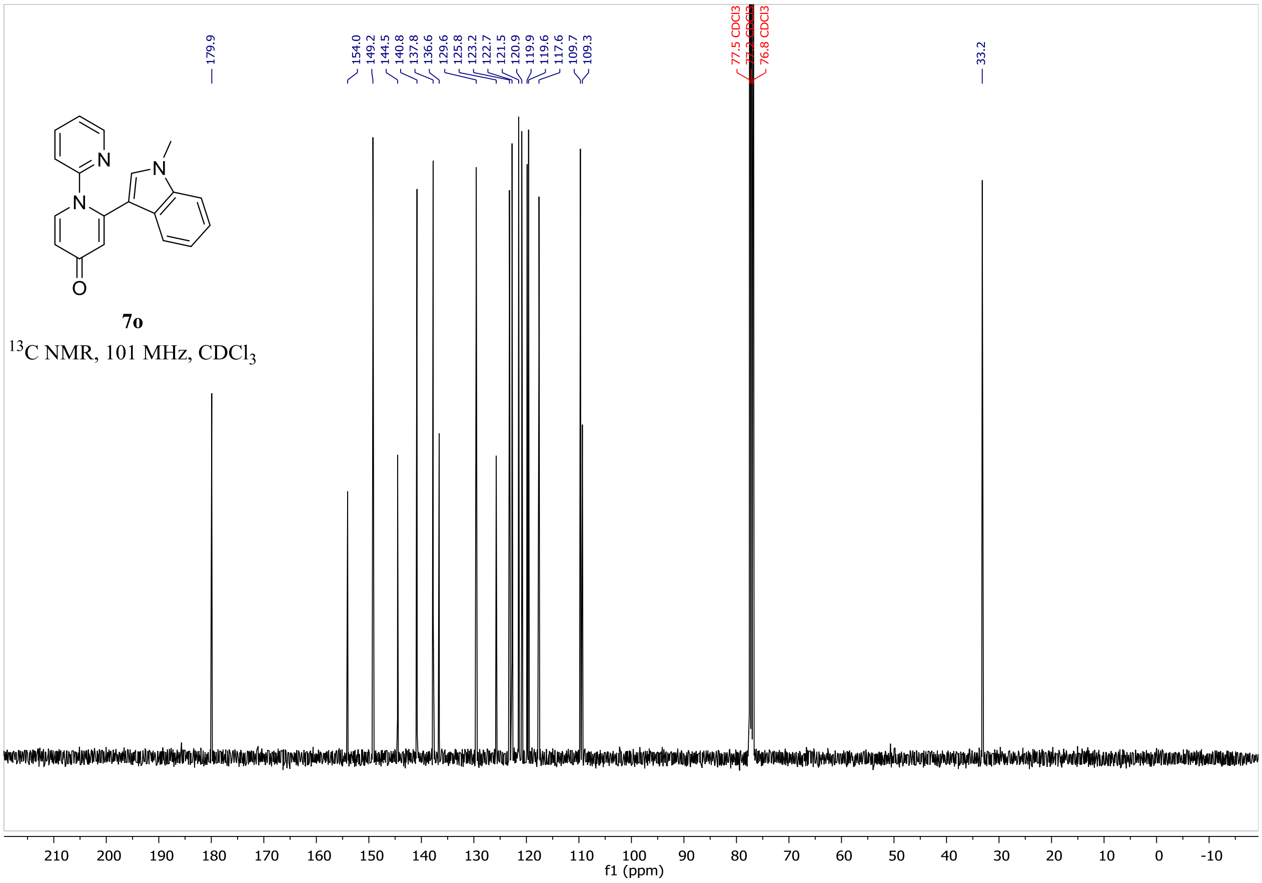

7o
^{13}C NMR, 101 MHz, $CDCl_3$
f1 (ppm)

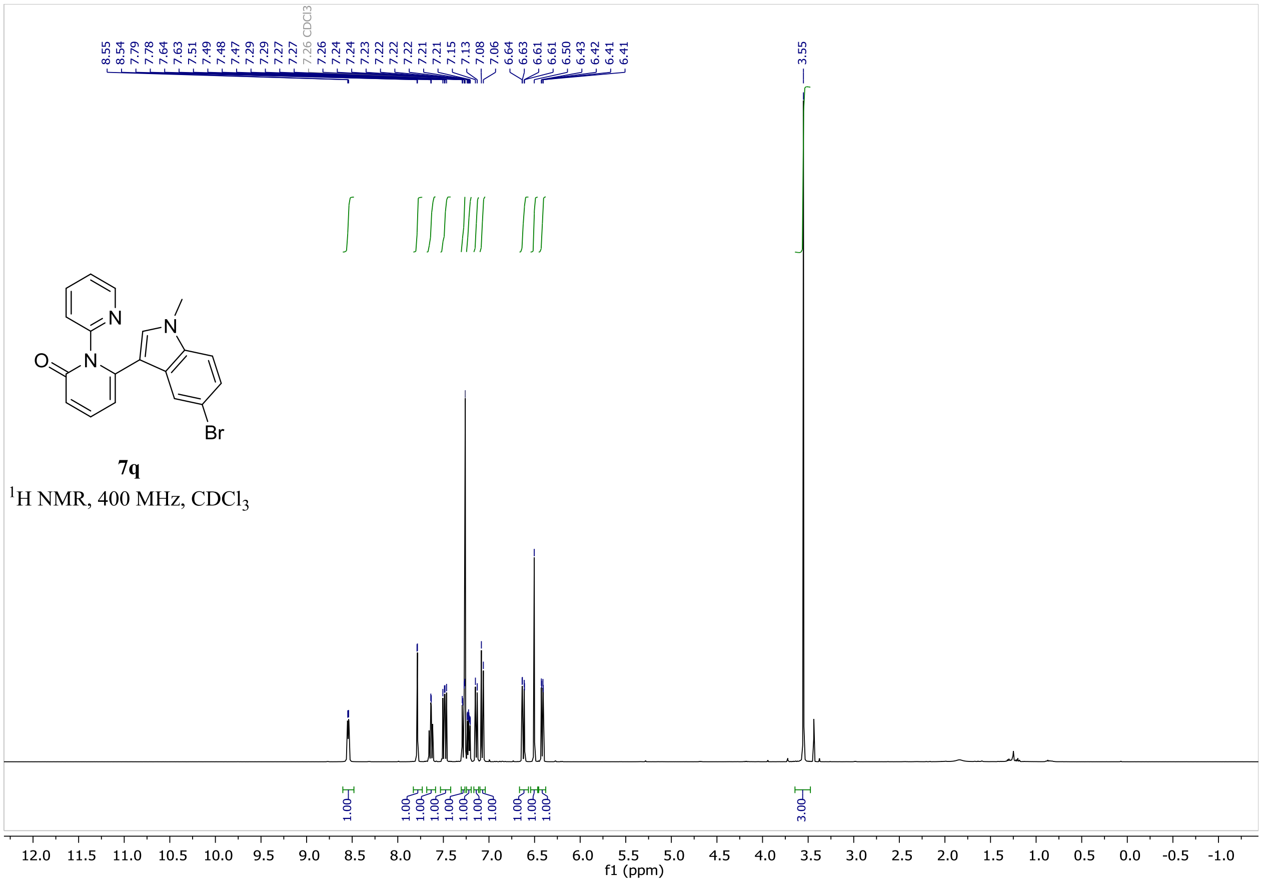

7q

^{1}H NMR, 400 MHz, $CDCl_3$

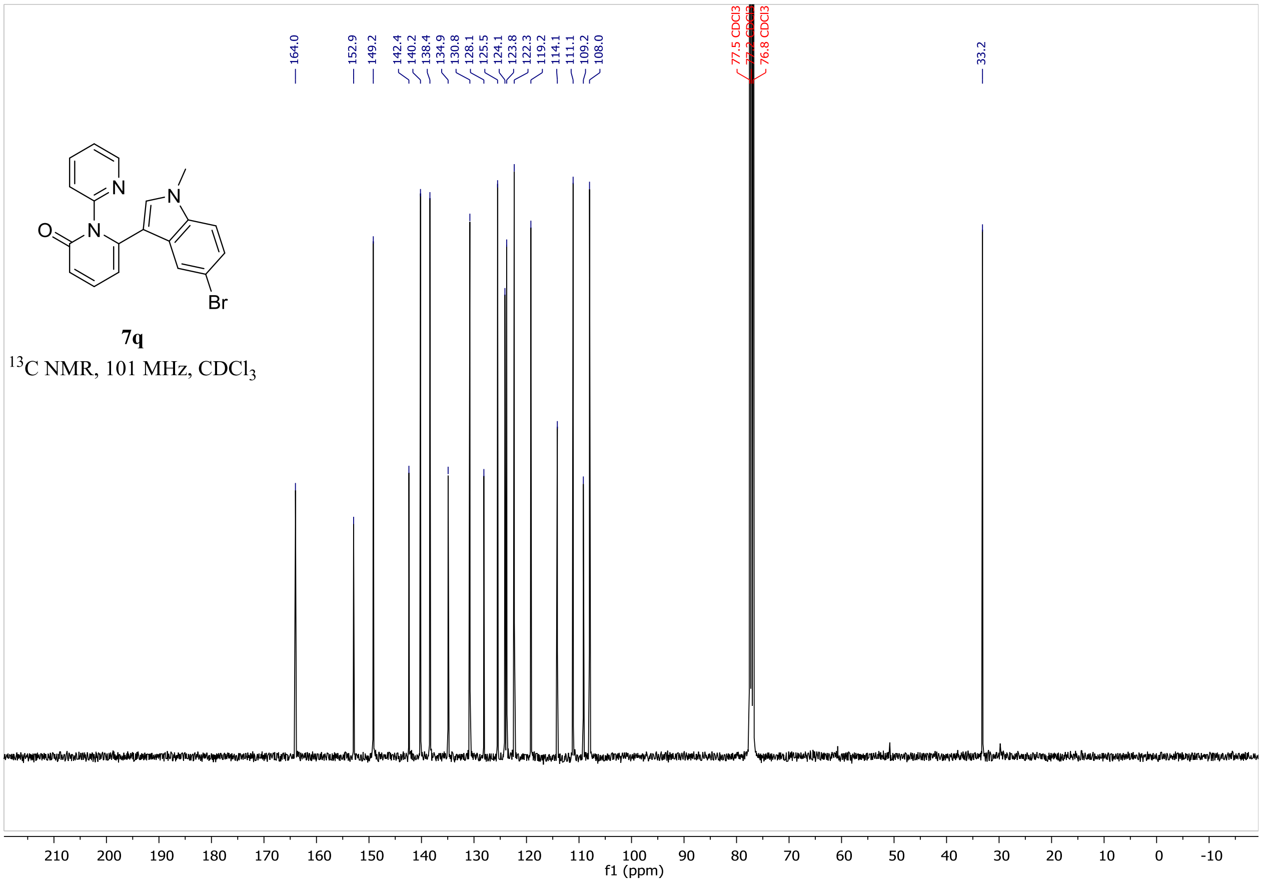

7q

^{13}C NMR, 101 MHz, $CDCl_3$

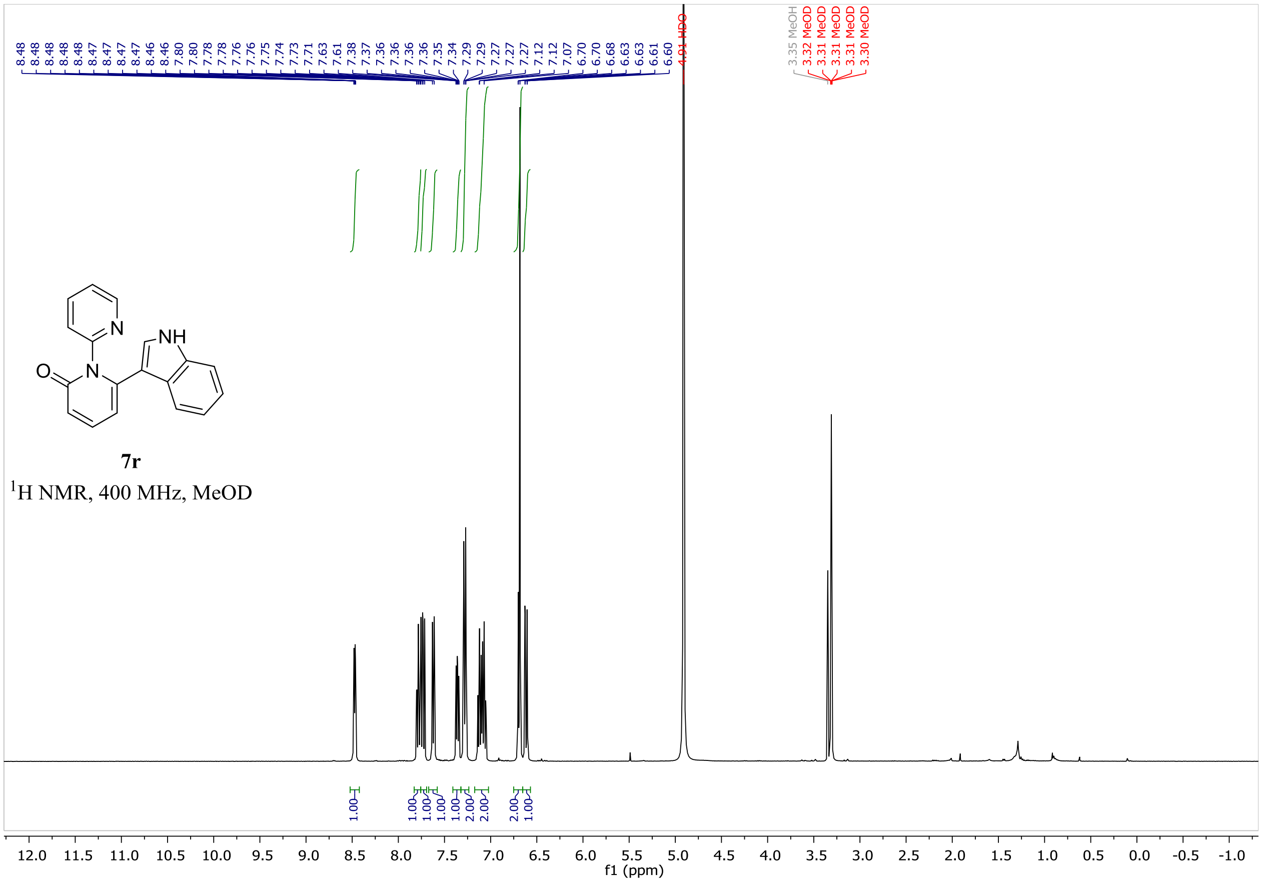
8.48 8.48 8.48 8.48 8.48 8.47 8.47 8.47 8.46 8.46 7.80 7.80 7.78 7.78 7.76 7.76 7.75 7.74 7.73 7.71 7.63 7.61 7.38 7.37 7.36 7.36 7.36 7.36 7.35 7.34 7.29 7.29 7.27 7.27 7.27 7.12 7.12 7.07 6.70 6.70 6.68 6.63 6.63 6.61 6.60
4.91 HDO
3.35 MeOH
3.32 MeOD
3.31 MeOD
3.31 MeOD
3.31 MeOD
3.30 MeOD
NH
N
O
7r
^{1}H NMR, 400 MHz, MeOD
1.00 1.00 1.00 1.00 1.00 2.00 2.00 2.00 1.00
12.0 11.5 11.0 10.5 10.0 9.5 9.0 8.5 8.0 7.5 7.0 6.5 6.0 5.5 5.0 4.5 4.0 3.5 3.0 2.5 2.0 1.5 1.0 0.5 0.0 -0.5 -1.0
f1 (ppm)

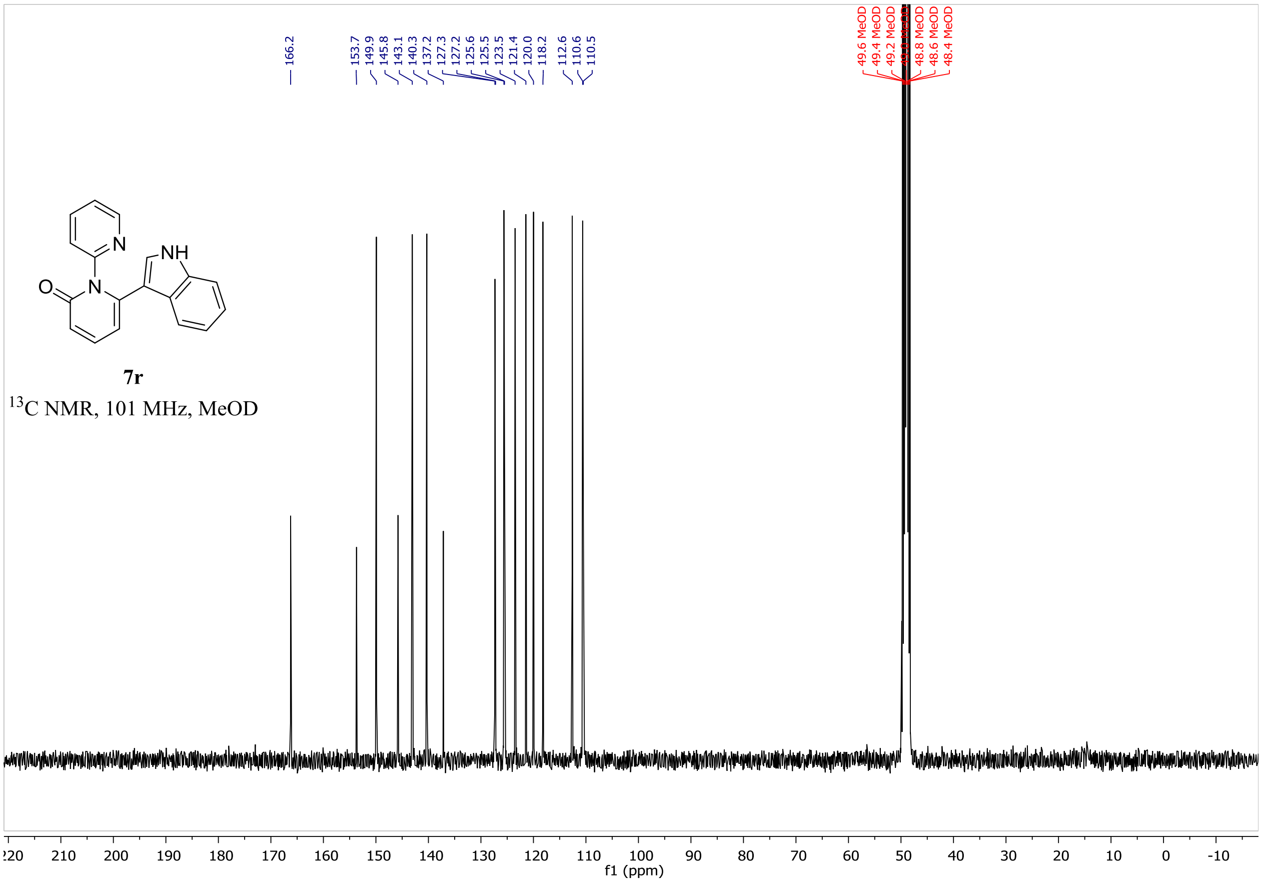
166.2
153.7 149.9 145.8 143.1 140.3 137.2 127.3 127.2 125.6 125.5 123.5 121.4 120.0 118.2 112.6 110.6 110.5
49.6 MeOD
49.4 MeOD
49.2 MeOD
48.8 MeOD
48.6 MeOD
48.4 MeOD
NH
N
O
7r
^{13}C NMR, 101 MHz, MeOD
220 210 200 190 180 170 160 150 140 130 120 110 100 90 80 70 60 50 40 30 20 10 0 -10
f1 (ppm)

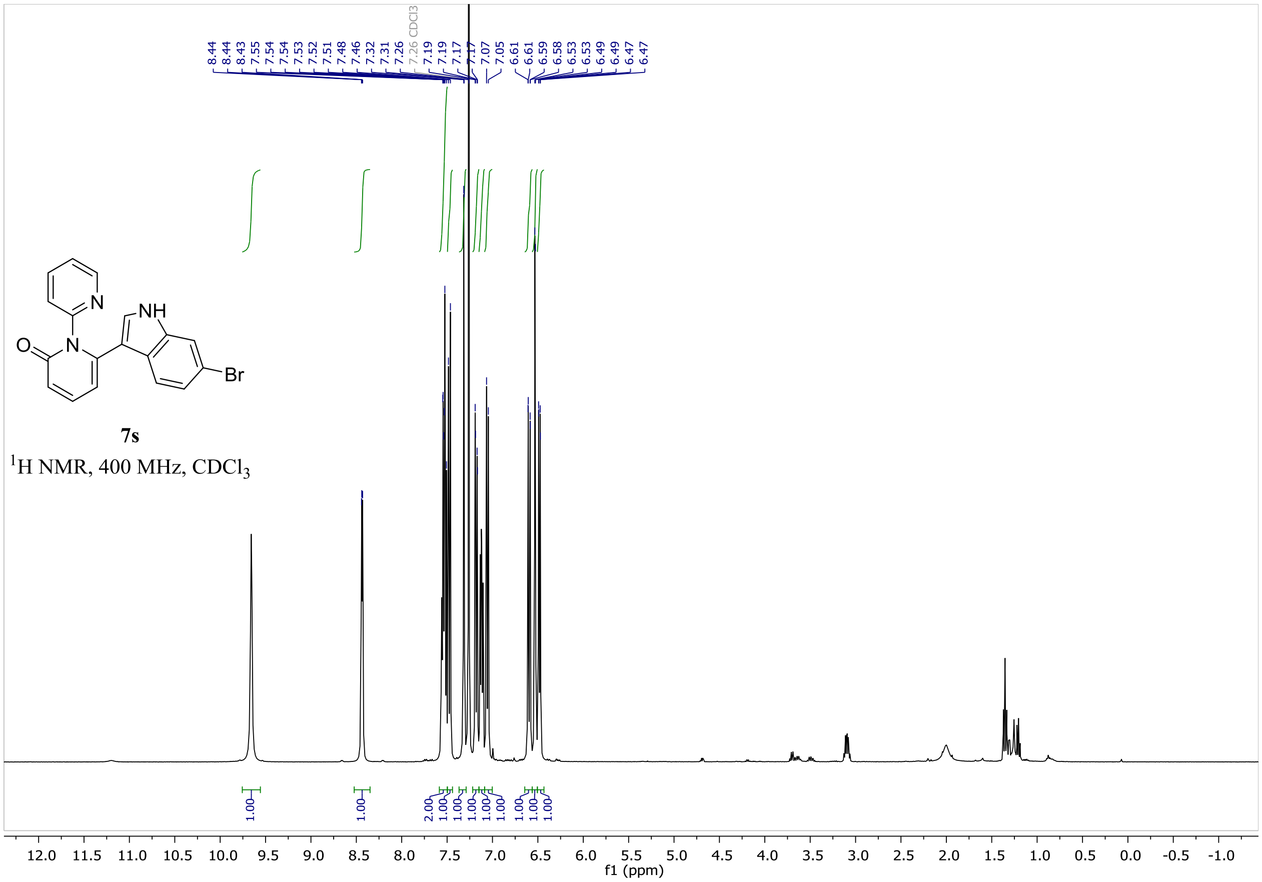

7s

^{1}H NMR, 400 MHz, $CDCl_3$

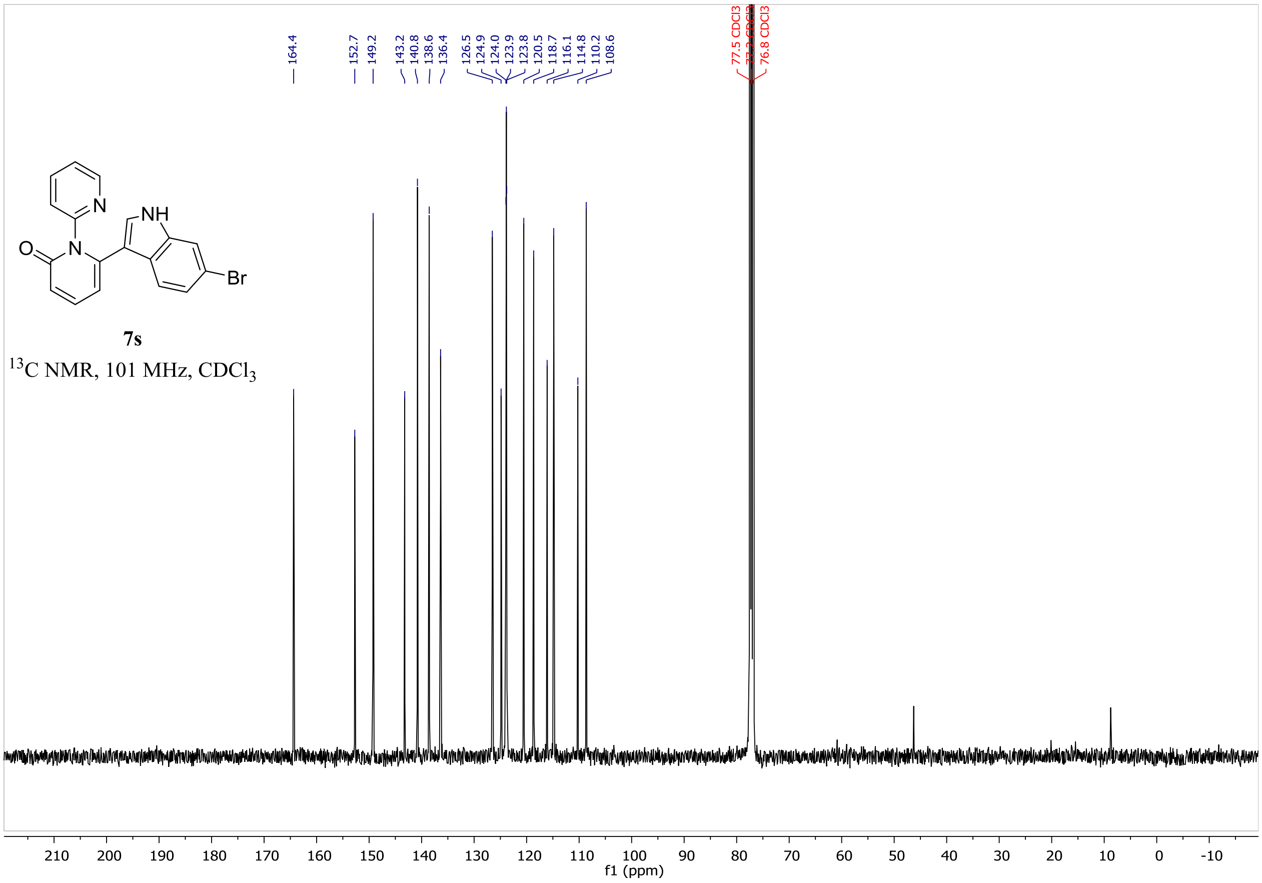

7s

^{13}C NMR, 101 MHz, $CDCl_3$

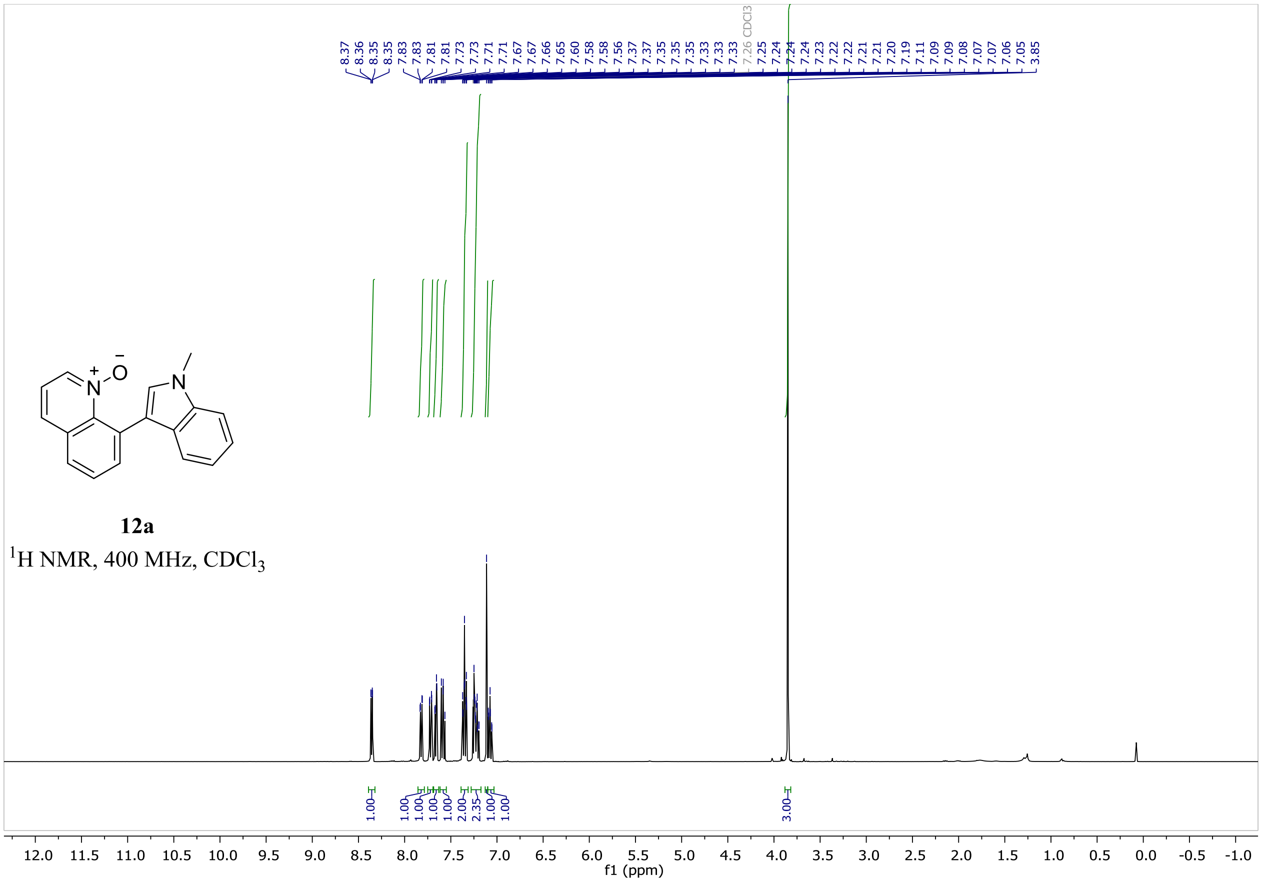

12a

^{1}H NMR, 400 MHz, $CDCl_3$

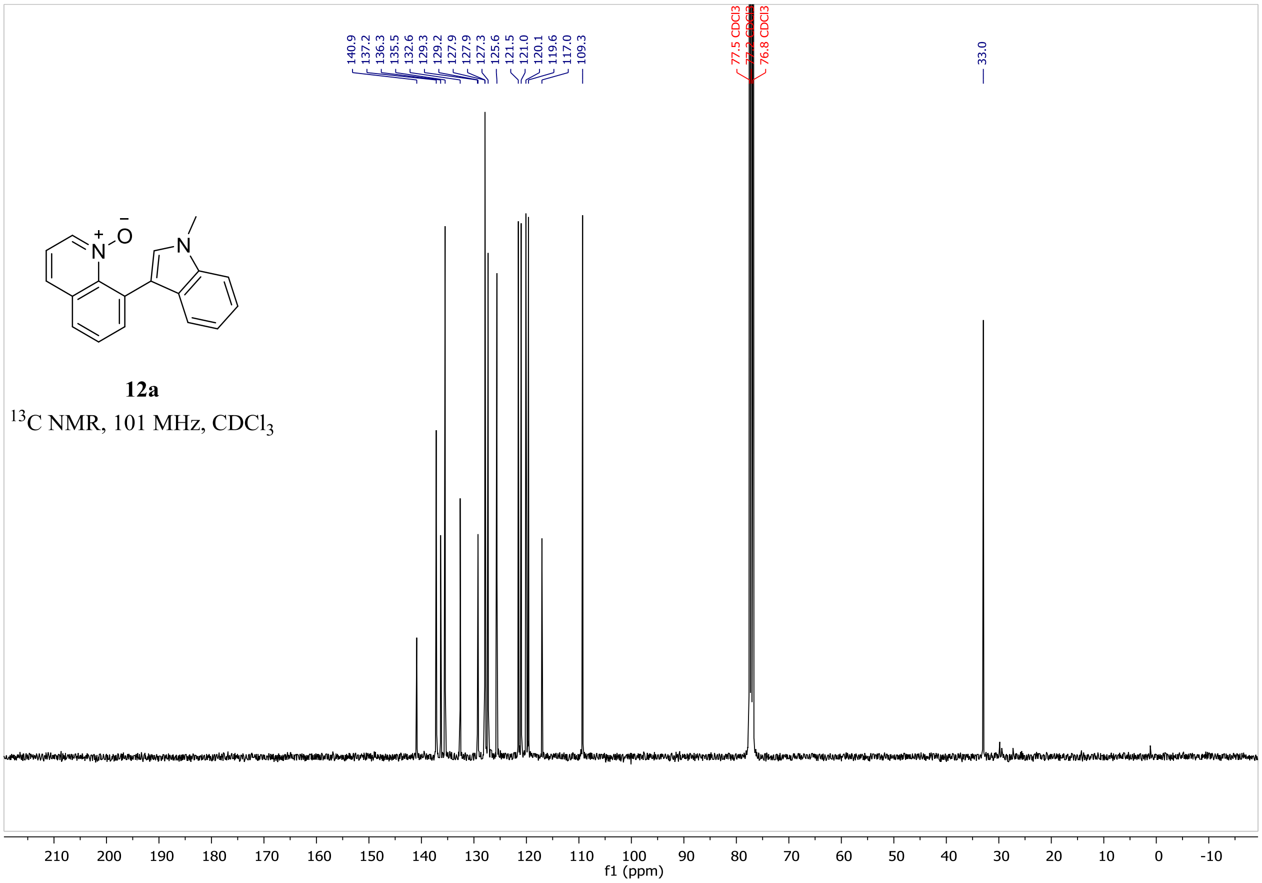

12a

^{13}C NMR, 101 MHz, $CDCl_3$

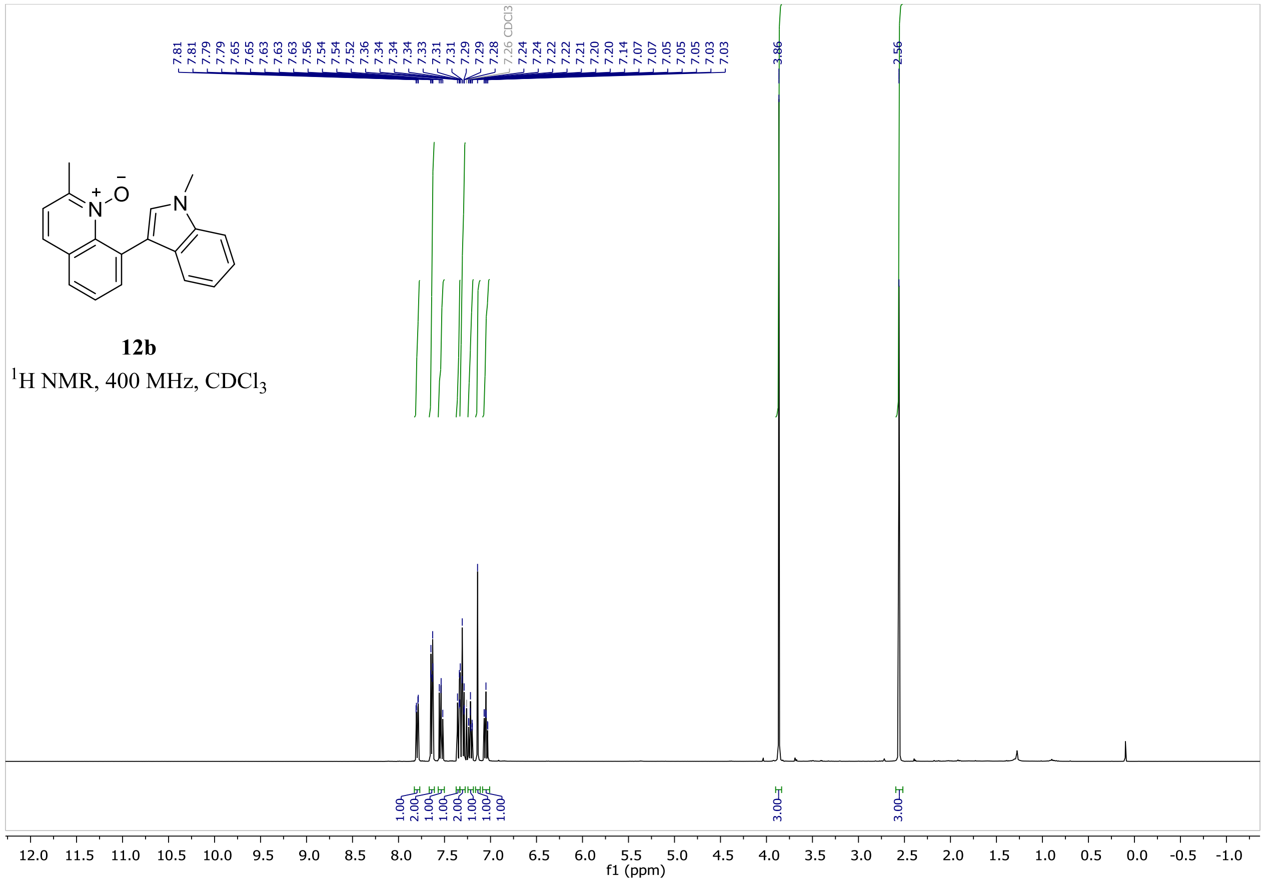
7.81 7.81 7.79 7.79 7.65 7.65 7.63 7.63 7.63 7.56 7.54 7.54 7.52 7.36 7.34 7.34 7.34 7.33 7.31 7.31 7.29 7.29 7.28 7.26 CDCl3 7.24 7.24 7.22 7.22 7.21 7.20 7.20 7.14 7.07 7.07 7.05 7.05 7.03 7.03
3.86
2.56
1.00 2.00 1.00 1.00 2.00 1.00 1.00 1.00 1.00
3.00
3.00
N
O
12b
1H NMR, 400 MHz, CDCl3
f1 (ppm)

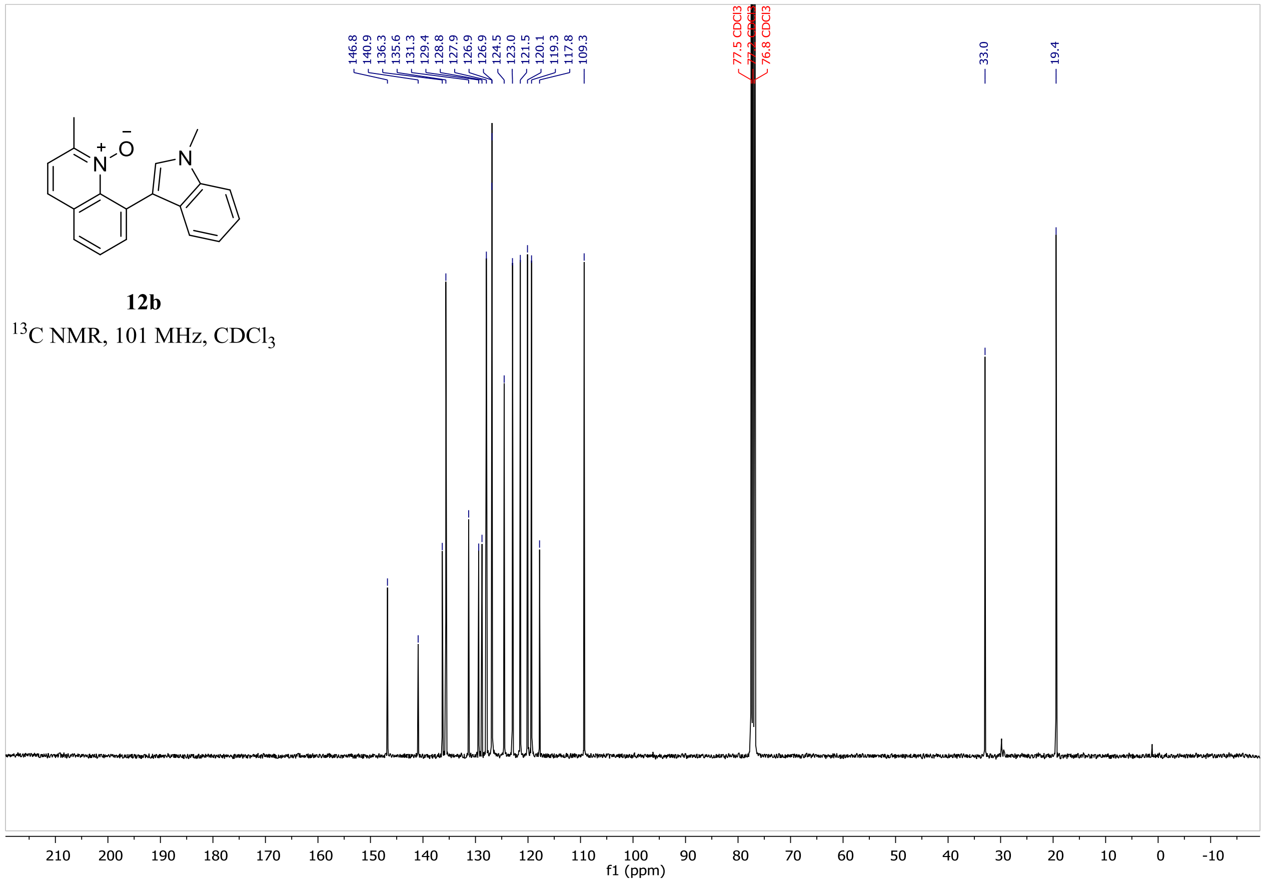
146.8 140.9 136.3 135.6 131.3 129.4 128.8 127.9 126.9 126.9 124.5 123.0 121.5 120.1 119.3 117.8 109.3
77.5 CDCl3 77.2 CDCl3 76.8 CDCl3
33.0
19.4
N
O
12b
13C NMR, 101 MHz, CDCl3
f1 (ppm)

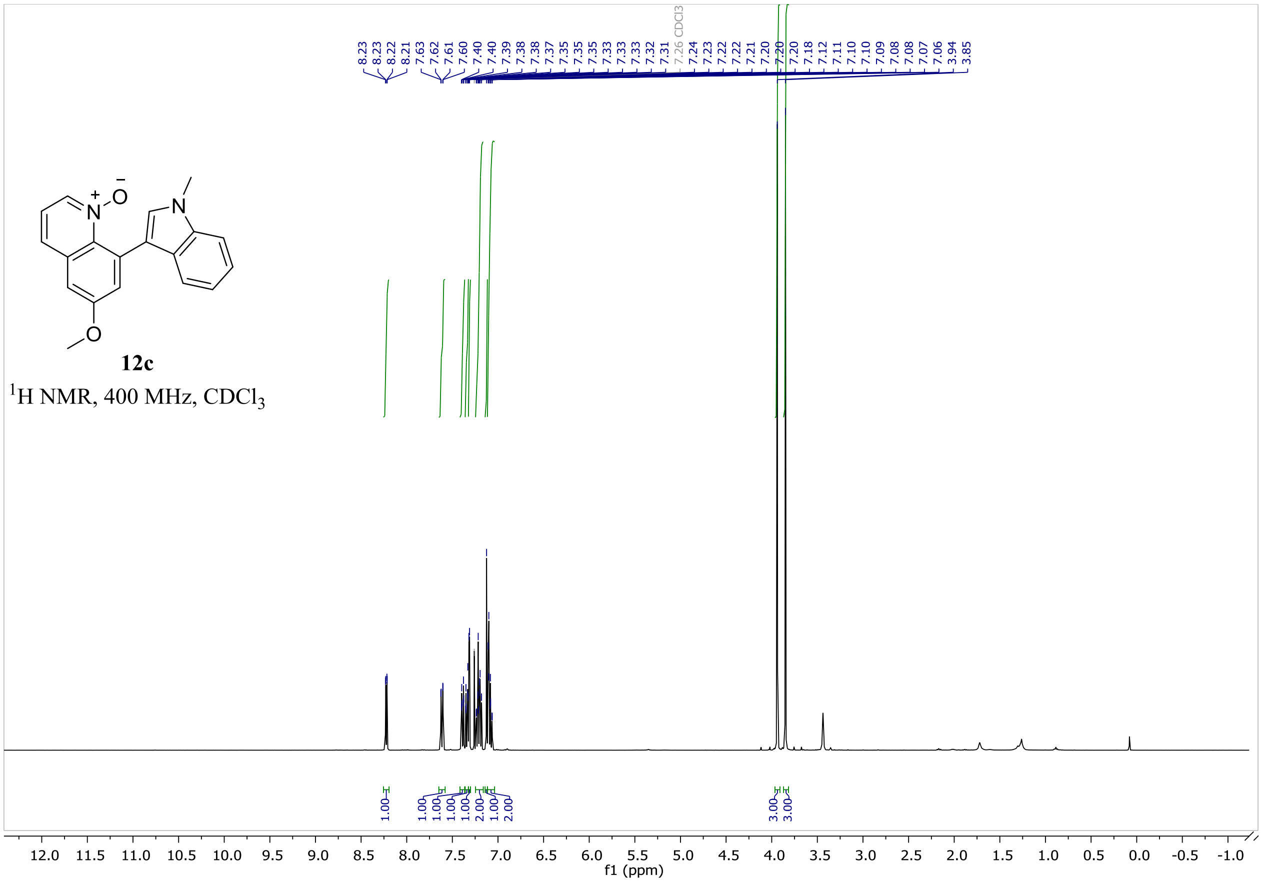

12c

^{1}H NMR, 400 MHz, $CDCl_3$

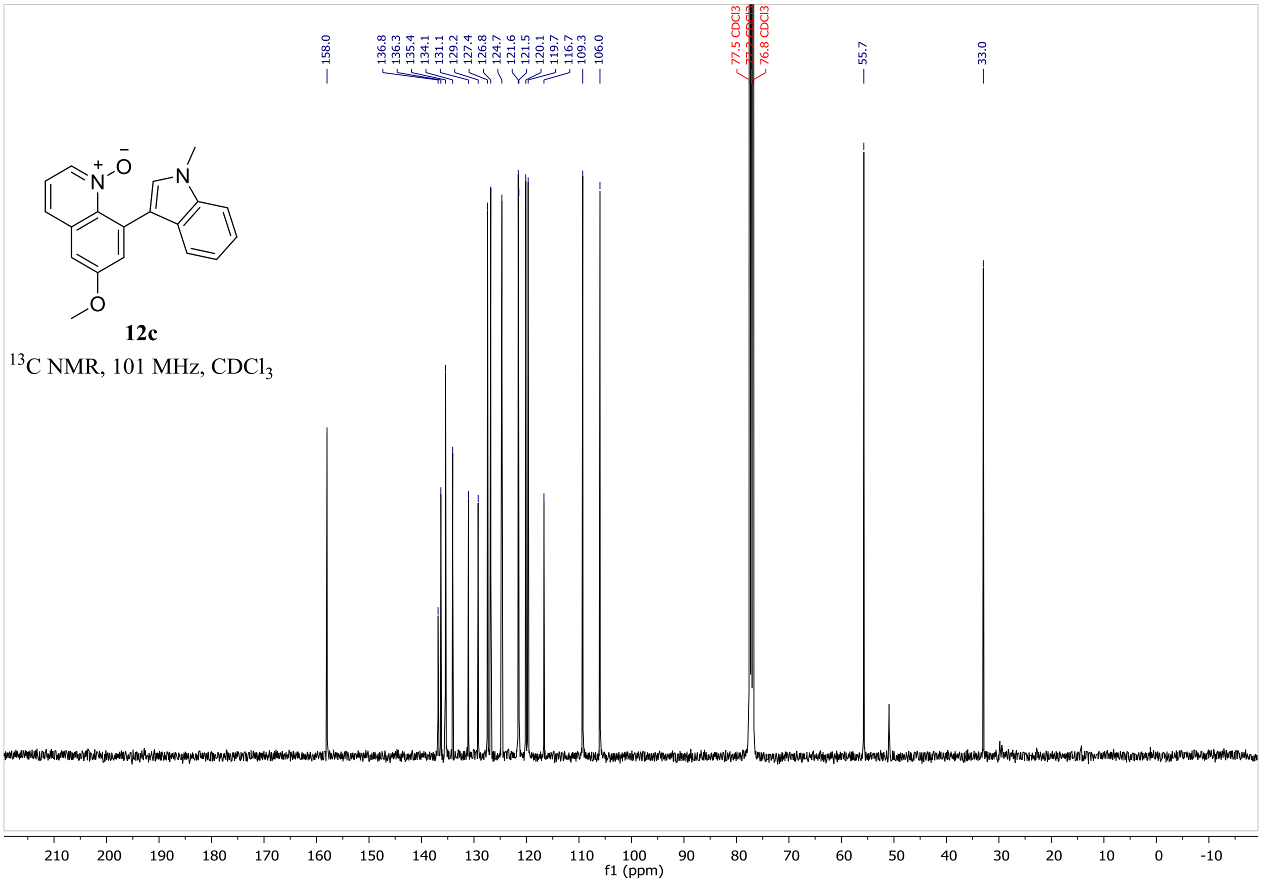

12c

^{13}C NMR, 101 MHz, $CDCl_3$

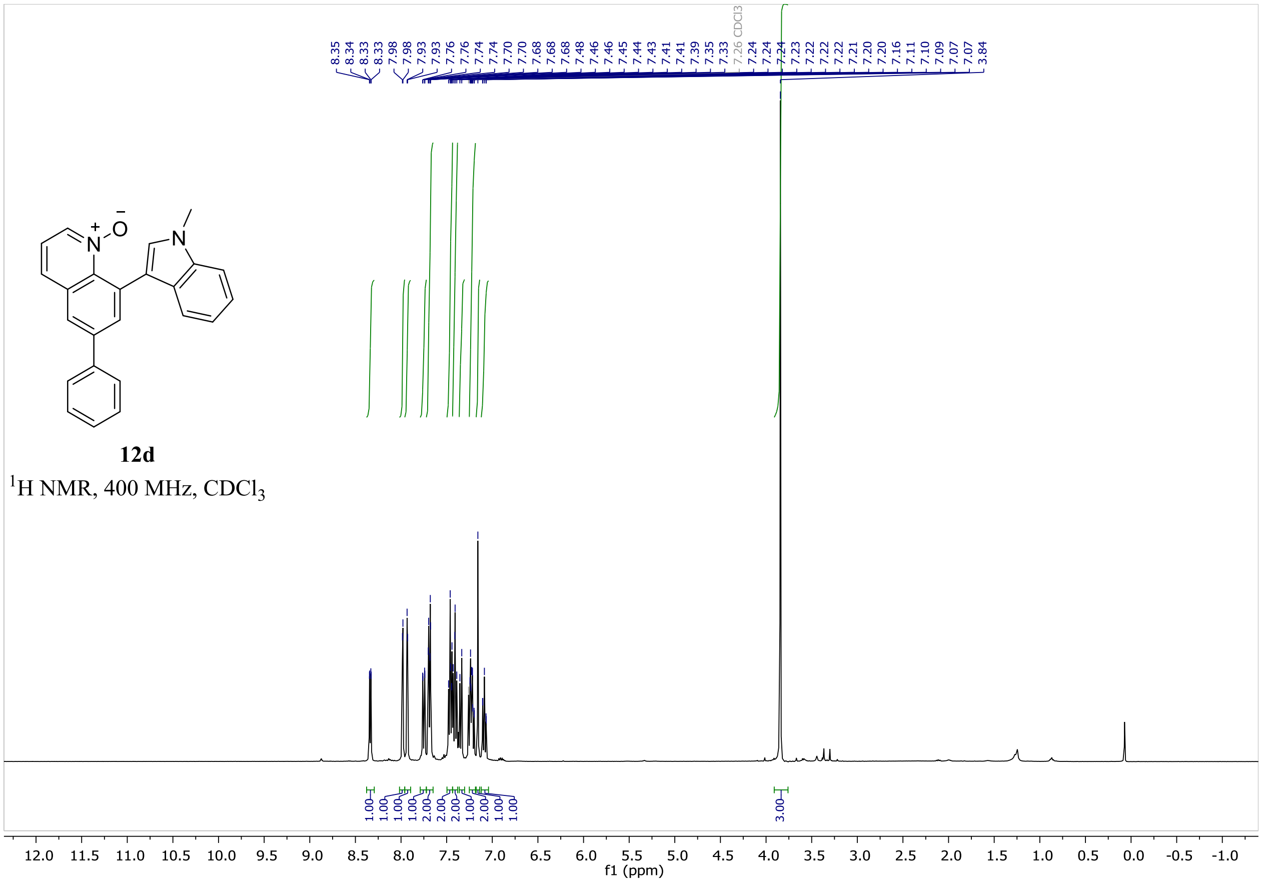

12d

^{1}H NMR, 400 MHz, $CDCl_3$

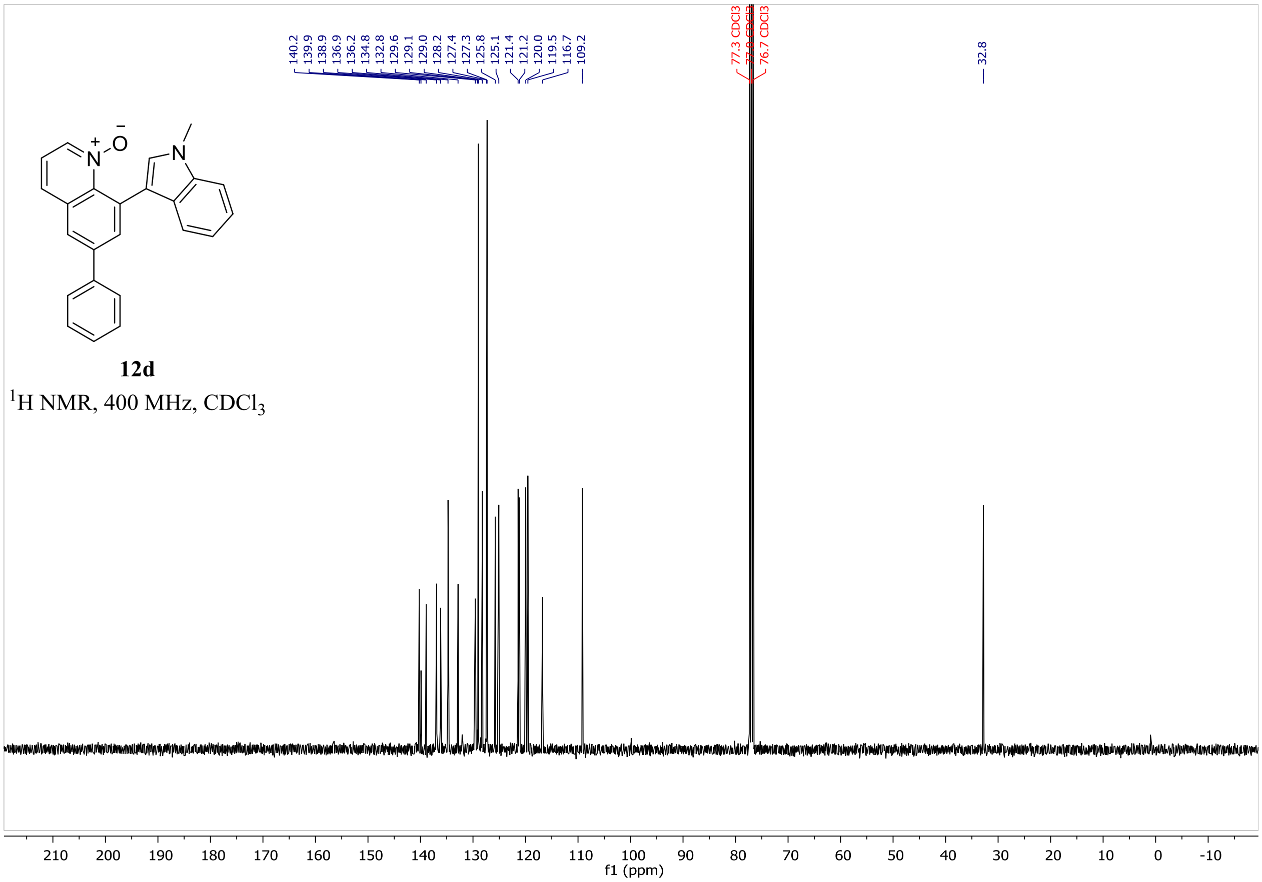

12d

^{1}H NMR, 400 MHz, $CDCl_3$

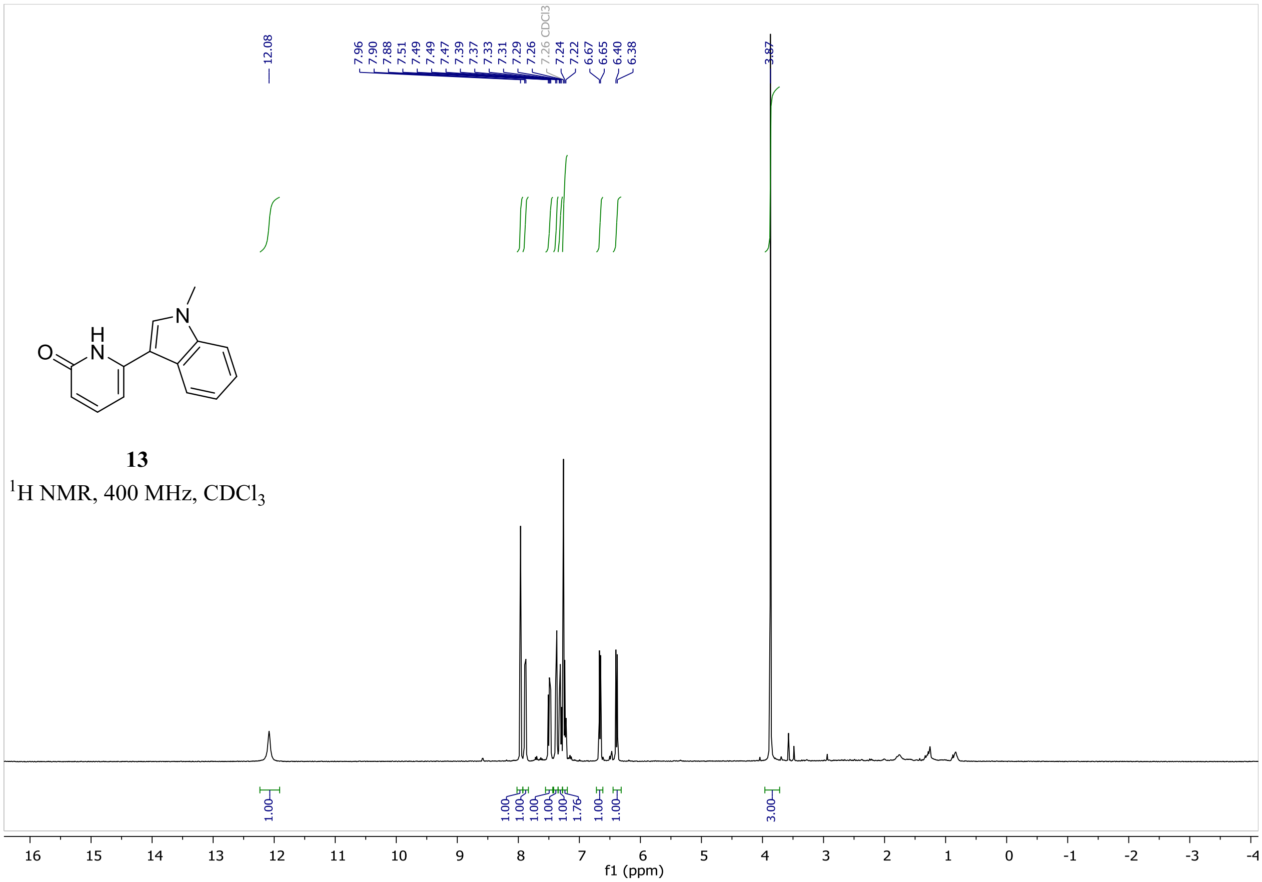
12.08
7.96
7.90
7.88
7.51
7.49
7.49
7.47
7.39
7.37
7.33
7.31
7.29
7.26
7.26 CDCl3
7.24
7.22
6.67
6.65
6.40
6.38
3.87
13
^{1}H NMR, 400 MHz, $CDCl_3$
1.00
1.00
1.00
1.00
1.00
1.00
1.76
1.00
1.00
3.00
f1 (ppm)

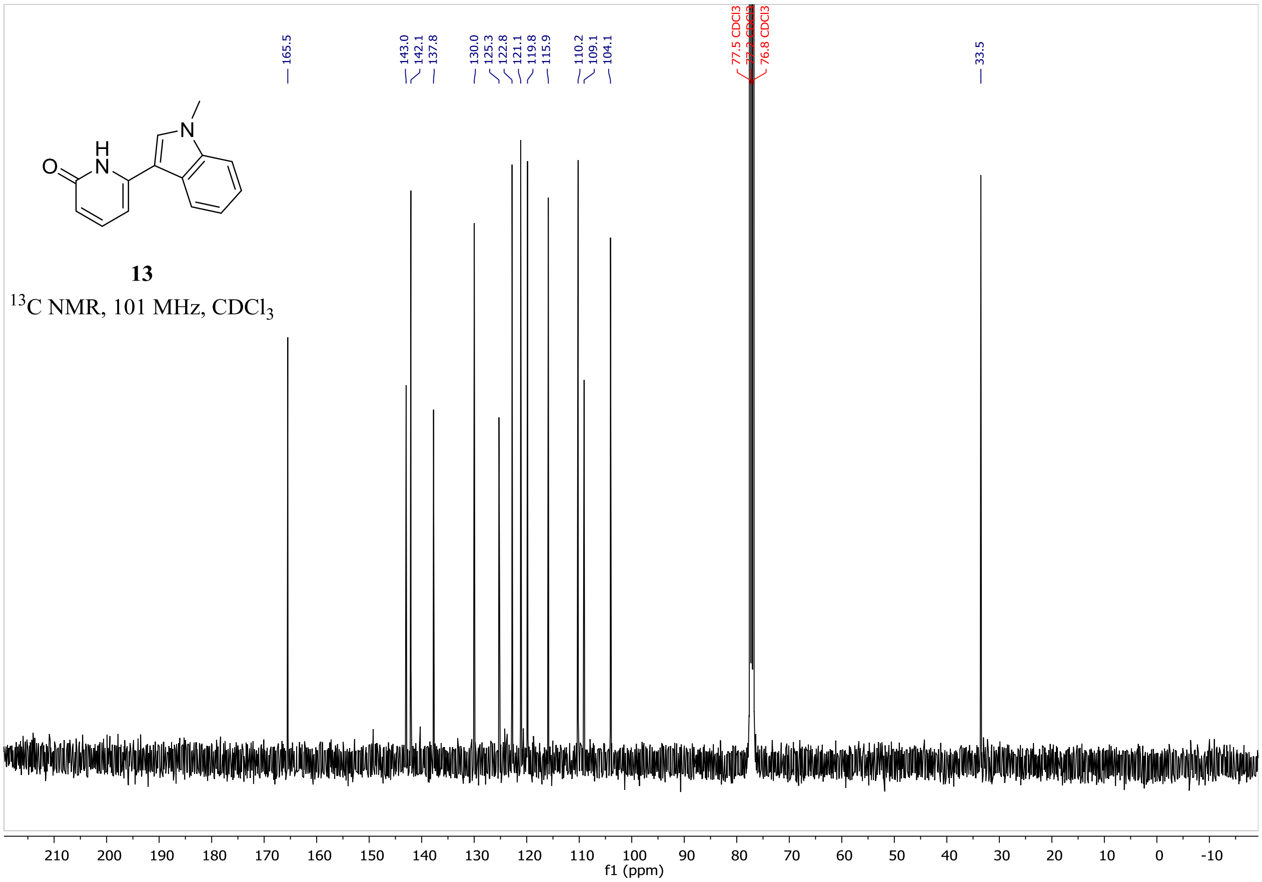
165.5
143.0
142.1
137.8
130.0
125.3
122.8
121.1
119.8
115.9
110.2
109.1
104.1
77.5 CDCl3
76.8 CDCl3
33.5
13
^{13}C NMR, 101 MHz, $CDCl_3$
f1 (ppm)
